# Investigating the Hydrogen Bond-Induced Self-Assembly of Polysulfamides Using Molecular Simulations and Experiments

**DOI:** 10.1021/acs.macromol.3c01093

**Published:** 2023-06-28

**Authors:** Zijie Wu, Jiun Wei Wu, Quentin Michaudel, Arthi Jayaraman

**Affiliations:** † Department of Chemical and Biomolecular Engineering, University of Delaware, 150 Academy St., Newark, Delaware 19716, United States; ‡ Department of Chemistry, Texas A&M University, College Station, Texas 77843, United States; § Department of Materials Science & Engineering, Texas A&M University, College Station, Texas 77843, United States; ∥ Department of Materials Science and Engineering, University of Delaware, 201 DuPont Hall, Newark, Delaware 19716, United States

## Abstract

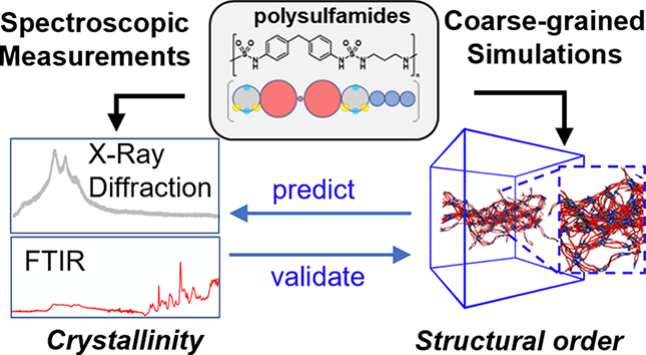

In this paper, we present a synergistic, experimental, and computational study of the self-assembly of *N*,*N*′-disubstituted polysulfamides driven by hydrogen bonds (H-bonds) between the H-bonding donor and acceptor groups present in repeating sulfamides as a function of the structural design of the polysulfamide backbone. We developed a coarse-grained (CG) polysulfamide model that captures the directionality of H-bonds between the sulfamide groups and used this model in molecular dynamics (MD) simulations to study the self-assembly of these polymers in implicit solvent. The CGMD approach was validated by reproducing experimentally observed trends in the extent of crystallinity for three polysulfamides synthesized with aliphatic and/or aromatic repeating units. After validation of our CGMD approach, we computationally predicted the effect of repeat unit bulkiness, length, and uniformity of segment lengths in the polymers on the extent of orientational and positional order among the self-assembled polysulfamide chains, providing key design principles for tuning the extent of crystallinity in polysulfamides in experiments. Those computational predictions were then experimentally tested through the synthesis and characterization of polysulfamide architectures.

## 1 Introduction

Small molecules containing a sulfamide moiety^1−3^ have gained a great amount of attention in medicinal chemistry,^4^ organocatalysis,^5−9^ and molecular assembly.^10,11^ By contrast, macromolecules with repeating sulfamide linkages (i.e., polysulfamides) have been seldomly synthesized,^12−15^ preventing an in-depth exploration of their structure and properties. Michaudel and co-workers recently reported a general approach toward the synthesis of asymmetric sulfamides via sulfur(VI)-fluoride exchange (SuFEx) click chemistry,^16^ which enables access to a wide range of polysulfamide structures via two polymerization processes.^17,18^ The synthesized polysulfamides exhibit high thermal stability, tunable glass transition temperature (*T*_g_), and structure-dependent crystallinity. These synthesized polysulfamides were also shown to be degradable in aqueous conditions. These attractive properties of polysulfamides, the −SO_2_– analogues of polyureas, make polysulfamide a potential replacement for polyurea, a high-commodity plastic.^19−21^

Structural characterization of the synthesized polysulfamides using Fourier-transform infrared (FTIR) spectroscopy and powder X-ray diffraction (PXRD) has suggested a correlation between the crystallization process and intermolecular H-bonding interactions. It is hypothesized that the interchain H-bonds formed between the donor N–H and acceptor −SO_2_– can be affected by chain features (e.g., bulkiness, length, and stiffness of the repeating unit) as well as other weak interactions (e.g., π–π stacking interactions between aromatic rings). Because the assembled polymer structure and extent of crystallinity dictate the macroscopic properties of the soft material, understanding the relationships between the chemical structure of the polymer backbone, interchain H-bonding interactions, and self-assembly will be crucial to effectively tailor the properties of polysulfamides for specific applications. Computational methods, such as molecular modeling and simulation, complement experiments in such fundamental investigations and can accelerate the identification of the next generation of plastics.

Molecular modeling and simulations can elucidate underlying driving forces and assembly mechanisms and enable efficient screening of large sets of molecular design parameters;^22^ however, capturing localized H-bonding interactions alongside macromolecular length and time scales has been a challenge in molecular simulations (see recent viewpoint on this topic^23^). Atomistic simulations retain the chemical details necessary for the directionality of H-bonds, but they are often too computationally intensive for capturing the macromolecular time and length scales needed to study polymer assembly into/disassembly from an ordered morphology. One way to overcome these limitations of atomistic models is to employ coarse-grained (CG) models in which groups of atoms, functional groups, or even monomers are collectively represented as CG “beads”, thereby reducing the computational intensity for simulating the macromolecular system with reduced degrees of freedom. Such CG models with isotropic potentials modeling interactions between CG beads have been developed for DNAs,^24−29^ proteins,^30−35^ polysaccharides,^36−41^ and synthetic polymers such as polyureas,^42−44^ the carbonyl analogue of polysulfamides. One of the major limitations of such lumping of atoms into CG beads that interact isotropically is that the relevant chemical details that induce the directionality of H-bonds can be lost. This in turn can lead to the prediction of incorrect self-assembled structures due to missing “valency” created by the directionality and finite number of H-bonding groups in the polymer chains. To overcome this limitation, some CG models take a more informed approach when distributing the atoms into CG beads and “implicitly” recognize the directional and specific nature of H-bonds by intentionally allocating more computational resources toward simulating chemical groups participating in H-bonds. As a prominent example, MARTINI,^45^ a CG model that groups atoms into categories of CG beads with different levels of polarity as well as propensity to H-bond (e.g., “donor”, “acceptor”, “donor and acceptor”, or “not involved in H-bonds”), can used as a baseline CG modeling scheme that can be optimized further for specific chemistries (e.g., see examples in a recent review on polymer simulations using the MARTINI CG model.^46^) The two major drawbacks of MARTINI CG modeling are the cumbersome additional optimization for the specific chemistry of interest and the more detailed representation of the polymers (e.g., multiple CG beads to represent one monomer) that can limit the time scales of the simulation. As an alternate approach to implicitly model the H-bonding effect, hybrid models that use all-atom resolution for “critical” atoms participating in H-bonds and CG resolution otherwise have also been developed.^47−49^ However, the increased computational cost and difficulty in atom-typing and force-field tuning for such hybrid models with multiple degrees of resolutions are nontrivial and can be significant barriers to using such hybrid atomistic-CG models for predicting design rules for future synthesis.

If the scientific question at hand demands that the anisotropy of H-bonding interaction be *explicitly* addressed in the CG model, one can choose to keep the efficient spherical CG bead representation and isotropic interaction potentials for the non-H-bond forming CG beads but model the interaction between H-bond forming CG beads with the less computationally efficient, anisotropic interaction potentials. Examples of anisotropic interaction potentials include the Gay–Berne potential,^50^ angle-based potential for DNA base pairing,^51−54^ or the angle-specific potential borrowed from the Mercedes-Benz water model.^55,56^ Alternatively, one can also avoid the computationally intensive anisotropic interaction potentials by inducing directionality through optimization of size and placement of *isotropically* interacting H-bonding CG beads (i.e., “sticky” sites or patches) embedded within a larger CG bead. These “sticky” site CG models have the benefit of capturing the isolated effect of H-bonding along with/in contrast to the other isotropic interactions in the system while retaining the computational efficiency of the simulation necessary to capture large time and length scales of macromolecular assembly/disassembly. This type of “sticky” site CG model has been successfully used in simulations by Sciortino and co-workers,^57,58^ Vargas Lara and Starr,^59^ Bochicchio and Pavan,^60,61^ Travesset and co-workers,^62,63^ Jayaraman and co-workers,^64−74^ and others. We choose to extend this type of “sticky” site CG model for polysulfamides to capture the dominant H-bonding interactions between polysulfamide chains that drive assembly and crystallinity in the assembled structure.

In past computational studies, polymer crystallization has been studied using both Monte Carlo (MC) and molecular dynamics (MD) simulations; these studies have focused on the nucleation mechanism,^75−79^ growth mechanism,^80−82^ precursor state prior to nucleation,^75,81,83^ role of chain entanglement,^80,84−86^ branching,^87^ cross-linking,^88^ and crystallization of short polymers chains (20 CH_2_ monomers).^77,89,90^ One of the many challenges in simulating polymer crystallization is the need for a specialized simulation protocol to initiate the nucleation and consistently maintain the growth of crystalline domain. Most of the simulations at least implement a carefully designed annealing or “quenching” that reduces the simulation temperature well below the melting temperature (*T*_m_), a procedure known as “subcooling”; other more complicated techniques have also been applied to initiate the nuclei as a first step, including placing crystal “seeds” or “nuclei template” directly in the initial configuration^82,91^ or implementing self-seeding procedure.^80^ As experimental characterization of polysulfamide samples exhibits semicrystalline domains (rather than 100% crystallinity), instead of going through these complex steps to reproduce crystallization in our simulation, we choose to predict the extent of the orientational and positional order in the self-assembled polysulfamide chains as proxy to qualitatively connect to experimentally observed semicrystallinity.

The remainder of this article is organized as follows. In Section 2, we describe our CG model, the design parameters of polysulfamides that we varied, our MD simulation protocol, analyses methods, and experimental synthesis and characterization procedures. In Section 3, we discuss the CG MD simulation results and compare them to experimental observations. In Section 4, we conclude by summarizing our findings and present several key design rules to promote the orientational and positional order of polysulfamide assembly.

## 2 Approach

### 2.1 Computational Approach

In this study, the development of the coarse-grained (CG) model was based on our hypothesis that H-bonds between sulfamide groups are the main driving force of polysulfamide assembly and that the nature of the repeating units affects the assembled structure by either disfavoring or promoting the H-bonding interactions. We leveraged our extensive past experience in modeling macromolecular assembly driven by H-bonds^64−74^ and constructed a CG model for polysulfamide that focuses on the generic effect of repeating unit properties (length, bulkiness, or stiffness) on the assembled structure of polysulfamide chains driven by H-bonding interactions between the sulfamide groups.

#### 2.1.1 Coarse Grained (CG) Model of Polysulfamide

##### 2.1.1.1 CG Bead Representation and Placement

**Figure 1:**
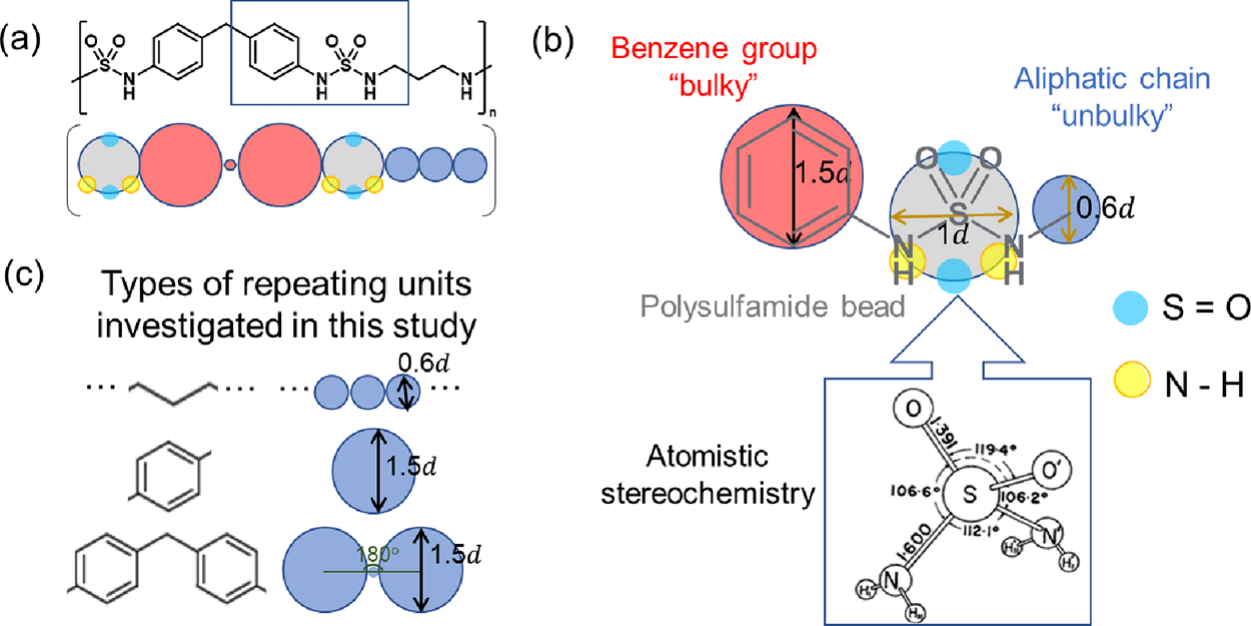
(a) One specific example of a polysulfamide repeating unit (with diphenylmethane and propyl group in the backbone) represented in an atomistic structure along with the coarse-grained (CG) model representation. (b) A detailed schematic of the region highlighted by the rectangle in part (a), with the CG representation of the sulfamide bead and its neighboring beads in repeating units overlaid on the atomistic structure. The schematic for atomistic stereochemistry is adapted from ref (2). (c) A list of different types of repeating units investigated in this study and their respective CG model representations.

The CG model of one specific example of polysulfamide is shown in Figure 1a. A more detailed schematic with the various bead placements for the sulfamide group and its neighboring repeating units is shown in Figure 1b. The CG model of the sulfamide group is made of five CG beads—a sphere of diameter 1*d* (shown in gray in Figure 1a,b), where *d* is approximately equal to 0.15 nm—two H-bond donor beads (shown in yellow in Figure 1a,b), and two H-bond acceptor beads (shown in cyan in Figure 1a,b). All four donor and acceptor beads are of diameter 0.2*d* and partially embedded in the gray sphere. The placement of the donor and acceptor beads is inspired by a proposed atomistic configuration of the sulfamide group,^2^ with the two N–H bonds residing on either side of the O=S=O plane. This CG model is generic, and the donor-sulfamide-donor and oxygen-sulfamide-oxygen apertures do not match the exact aperture angles as shown in the atomistic stereochemical configuration in Figure 1b; we refer the reader to Supporting Information Table S1 for the relative coordinates of the donor and acceptor beads with respect to the center of the parent sulfamide bead (the gray bead in Figure 1a,b) in our CG model. All beads in a sulfamide group—sulfamide bead, two donor beads, and two acceptor beads—are treated as a rigid body to be computationally efficient. The sulfamide gray sphere is connected to the CG beads representing the repeating units that vary in length, bulkiness, and stiffness (shown in red and blue in Figure 1a,b).

The number and size of CG beads in the repeating unit on either side of the sulfamide bead depend on the specific repeating unit chemistry we model. As shown in Figure 1c, we computed three general categories of repeating units for the polysulfamide chains: linear aliphatic chains, *p*-phenylenes, and benzhydryls (diarylated methane). To represent an aliphatic chain of *n* number of CH_2_ groups in the repeating unit, *n* connected CG beads of diameter 0.6*d* are used. To represent a phenylene in the repeating unit, a CG bead of diameter 1.5*d* is used. To represent a benzhydryl in the repeating unit, two CG beads of diameter 1.5*d*, representing the two aryl groups, are connected by a “bridge” bead of diameter 0.2*d*, representing the methylene (−CH_2_−) group connecting the two aryl groups.

##### 2.1.1.2 Bonded Interactions

We impose a set of bond, angle, and dihedral potentials to maintain CG bead connectivity within each polysulfamide chain and restrict unrealistic rotation of the various groups along the chains. Each pair of adjacent CG beads along the backbone of a polysulfamide chain (i.e., repeating unit bead–repeating unit bead or repeating unit bead–sulfamide bead), except the donor and acceptor beads, is connected by a harmonic bond potential represented as
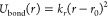
1with *k_r_* = 50 kT/*d*^2^ and *r*_0_ = (σ_i_ + σ_*j*_)/2, where σ_*i*_ and σ_*j*_ are the diameters of the two connected beads in units of *d*.

Except for the donor and acceptor beads, any three consecutive bonded beads along a polysulfamide chain (i.e., repeating unit bead–repeating unit bead–repeating unit bead, repeating unit bead–repeating unit bead–sulfamide bead, repeating unit bead–sulfamide bead– repeating unit bead, or sulfamide bead–repeating unit bead–repeating unit bead) are restricted in rotation by an angle potential represented as
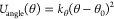
2where *k*_θ_=10 kT/rad^2^ and θ_0_ = 180^°^ (i. e. , π rad). The value of *k*_θ_ can be tuned to tailor the flexibility of the polysulfamide chain.^92^ In this study, we choose *k*_θ_=10 kT/rad^2^ not to mimic any experimentally determined persistence length but rather to provide a baseline constraint while maintaining a low but non-negligible level of stiffness in the chain backbone. We do not choose lower values of *k*_θ_ because, at those values, the flexible chains exhibit reduced positional and orientational order in the assembly of polysulfamide chains; experimental observation of semicrystalline domains informs us that this level of flexibility in the polymer chain is not correct. We note that within a repeating unit derived from benzhydryl (the angle explicitly drawn in Figure 1c), the *k*_θ_ used for the benzene bead–bridging methylene bead–benzene bead is set to 25 kT/rad^2^ to model the more restricted rotation in the case of benzene groups than other aliphatic groups.

We also apply additional constraints, including an angle potential and a dihedral potential, to maintain the proper orientation of the donor and acceptor beads on a sulfamide bead with respect to the backbone. We refer the reader to Supporting Information Section S.I. for the definition of these two additional potentials.

##### 2.1.1.3 Nonbonded Interactions

As done in our previous work for other hydrogen bonding dominant macromolecular systems,^64−74^ we capture an effectively directional H-bonding interaction between the donor and acceptor groups on the sulfamide bead by using an isotropic 12-6 Lennard–Jones potential^93^ between the small (compared to the sulfamide bead) donor and acceptor beads:
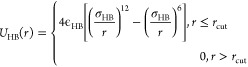
3with σ_HB_ = 0.2*d* (diameter of donor and acceptor beads) and *r*_cut_ = 2.5*d*. The value of ϵ_HB_, the strength of H-bond interaction, is gradually increased from 6.0 to 12.0 *kT* to ensure that the resulting self-assembly of polysulfamide chains is not kinetically trapped. These values of H-bonding strengths map to realistic H-bonding strengths. For example, the H-bond donor character of sulfamide has recently been shown to be slightly lower than those of thiourea and urea.^94^ Further, Hao^95^ calculated using DFT that the H-bonding strength between S=O and the amide donor (like the N–H donor in our work) is about 6.2 kcal/mol, which is ∼10.4 *kT* when *T* = 300 K.

As H-bonding interaction is hypothesized to be the dominant interaction, nonbonded interactions between all other pairs of beads are represented by isotropic, purely repulsive Weeks–Chandler–Andersen (WCA) potential:^96^
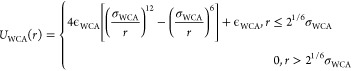
4with ϵ_WCA_ = 1 *kT* and σ_WCA_ = (σ_*i*_ + σ_*j*_)/2, where σ_*i*_ and σ_*j*_ are diameters of the *i* and *j* beads in units of *d*.

#### 2.1.2 Molecular Dynamics (MD) Simulation Protocol

We ran all MD simulations using the LAMMPS^97^ package in the NVT ensemble, with the number of CG beads in the simulation box (N), volume of the simulation box (V), and temperature (T) kept constant through the simulation. We used a reduced temperature T* = 1 set via the Nosé–Hoover thermostat.^98,99^ We prepared the initial configurations by placing 100 polysulfamide chains, with 10 sulfamide beads in each chain, in completely extended configurations in an initial simulation box of size 150*d* × 150*d* × 150*d* where *d* is the reduced unit of length and is equal to the diameter of a CG sulfamide bead. We first set the timestep size to 0.0002τ, where τ is the reduced unit of time; set all pairwise nonbonded interactions to repulsive-only using the WCA potential; and within 12.5 million timesteps slowly compressed the simulation box to 80*d* × 80*d* × 80*d* for simulations in groups I–III (see Section 2.3), and 40*d* × 40*d* × 40*d* for simulations in group IV. The larger simulation box in groups I–III is to accommodate the longer contour length of polymer chains involved in these groups and avoid self-interaction of chains across periodic boundaries of the simulation box. During this compression stage, the polymer chains relax their configurations away from the initial (unphysical) extended configurations as the system reaches the target simulation box size. We then set the timestep size to 0.001τ, set nonbonded pairwise interaction between donor beads and acceptor beads to attractive using the LJ potential, and carried out a simulated annealing process where ϵ_HB_, the interaction strength between donor and acceptor beads, was increased from 6.0 to 12.0 kT in increments of 0.2 kT every 7.5 million timesteps. This gradual increase in the ϵ_HB_ prevents the formation of kinetically trapped assembled polymer structures at high H-bonding strength. Further, keeping a constant T* and increasing the H-bonding strength ϵ_HB_ in simulations to induce assembly are effectively similar to experimentally decreasing the temperature to induce assembly and crystallization.

At each value of ϵ_HB_, the configurations in the last three million timesteps were used for production; we collected three configurations after every one million timesteps. At high ϵ_HB_, we expect the configurations in a single simulation run to be correlated due to the irreversible assembly formation. So, for each polysulfamide structure, we conducted three independent simulation trials to account for statistical fluctuation. We also compared the three independent trials to each other to ensure that the assembled structures resulting at the end of these three simulation trials are consistent to confirm that the structures used for analyses are not kinetically trapped.

#### 2.1.3 Analyses

We quantified the H-bonding propensity of the sulfamide beads and the resulting positional and orientational order within the assembled polysulfamide structures. The hydrogen bonding propensity (*f*_HB_) was calculated as the total number of H-bonds observed in the configuration divided by the total number of donor beads (or equivalently, total number of acceptor beads, as the number of donor beads is the same as the number of acceptor beads) in the simulation box. An H-bond is formed when the distance between the centers of a donor bead and an acceptor bead on two different sulfamide beads is less than 0.35*d.*

The intersegment angle between H-bonded segments (α_HB_) was used to quantify the alignment of the chains within the polysulfamide assembly. It was calculated for each pair of sulfamide beads that have at least one H-bond formed between them. For a sulfamide bead, we defined its corresponding sulfamide “segment” as the vector connecting the centers of that sulfamide bead and its adjacent repeating unit bead on the right. The angle between two such sulfamide segments, α_HB_, was calculated (see Supporting Information Figure S2 for details about the calculation). α_HB_ can take on values from 0 to 90°, and the head–tail orientation of the segment is irrelevant for this calculation. In the results section, we report the normalized distribution of α_HB_ ranging from 0 to 90°, with the normalizing factor being the total number of sulfamide beads in the simulation box squared (1000^2^ = 10^6^). These normalized distributions of α_HB_ quantify the orientational order in the system as we describe in the results section.

The radial distribution function [*g*(*r*)] was used to quantify the local positional order within polysulfamide assembly and is defined as
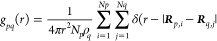
5where *p* and *q* are types of CG beads, *N_p_* is the number of CG beads of type *p*, ρ_*q*_ is the number density of *q*, and ***R***_*p*, *i*_ is the coordinate of the *i*th CG bead of type *p*. In this article, we focus on interchain sulfamide bead pair correlation, so sulfamide beads from the same chain are excluded from that *g*(*r*) calculation.

### 2.2 Experimental Approach

#### 2.2.1 Synthesis and Characterization of Polysulfamides

A series of polysulfamides were synthesized following our A_2_B_2_ polycondensation process with bis(sulfamoyl fluoride)s and bis(amine)s.^17^ All synthesized polymers were characterized via NMR and IR spectroscopies, as well as size-exclusion chromatography (SEC). The temperature of decomposition (*T*_d_, 5% weight loss) of each polysulfamide was measured by thermogravimetric analysis (TGA), and the phase transitions were investigated via differential scanning calorimetry (DSC). To experimentally validate the computationally predicted assembly of polysulfamides, several structural characterization methods were considered. Although numerous techniques including X-ray diffraction (XRD) or scattering, density measurement, DSC, FTIR, and solid-state NMR (ssNMR) can distinguish between amorphous and crystalline materials, the precise determination of the degree of crystallinity within polymeric materials requires established calibrations with samples of known crystallinity.^100^ For example, powder X-ray diffraction (PXRD) would require the synthesis and processing of both 0% crystalline (i.e., 100% amorphous) and 100% crystalline polysulfamides using for example the Ruland–Vonk method.^101,102^ These calibrations have yet to be established for polysulfamides because of the lack of reported data for this virtually unknown family of polymers. DSC is another standard method to quantify the degree of crystallinity in soft materials, but it too relies on the availability of polymeric standards with 100% crystallinity for the determination of the enthalpy or heat of fusion. Furthermore, we did not observe melting and crystallization temperatures by DSC for polysulfamides synthesized through SuFEx chemistry even for polymers exhibiting some crystallinity via PXRD.^17^ This might be the result of a melting transition taking place at temperatures above the scanning window of DSC, which is limited by the degradation temperature (*T*_d_) of the studied polymers. The samples studied by DSC might have partially crystallized upon quenching of the polymerization or during the workup rather than during the DSC cycle.

To circumvent the limitations for the quantitative measurement of crystallinity caused by the scarcity of characterization data on polysulfamides, we relied on FTIR to estimate the degree of crystallinity in combination with qualitative analysis via PXRD.^103,104^ FTIR has been previously employed to assess the crystallinity of polymers including poly(vinyl alcohol)^103^ and polyhydroxybuterate samples,^105^ but these studies remain limited compared to other characterization methods despite the high sensitivity of IR spectroscopy. Inspired by in-depth spectroscopical studies of the sulfamide molecule (H_2_NSO_2_NH_2_) coupled with single-crystal XRD previously reported in the literature,^106−108^ we used the relative intensity of the symmetric S=O stretch (∼1100 cm^–1^) and asymmetric S=O stretch (∼1300 cm^–1^) of the repeating sulfamide units as an indicator of the degree of crystallinity of several polysulfamides synthesized in this study that can be compared to theoretical predictions.

All experimental procedures and complete characterization data are described in Supporting Information Section S.III.

### 2.3 Polymer Design Space

**Figure 2:**
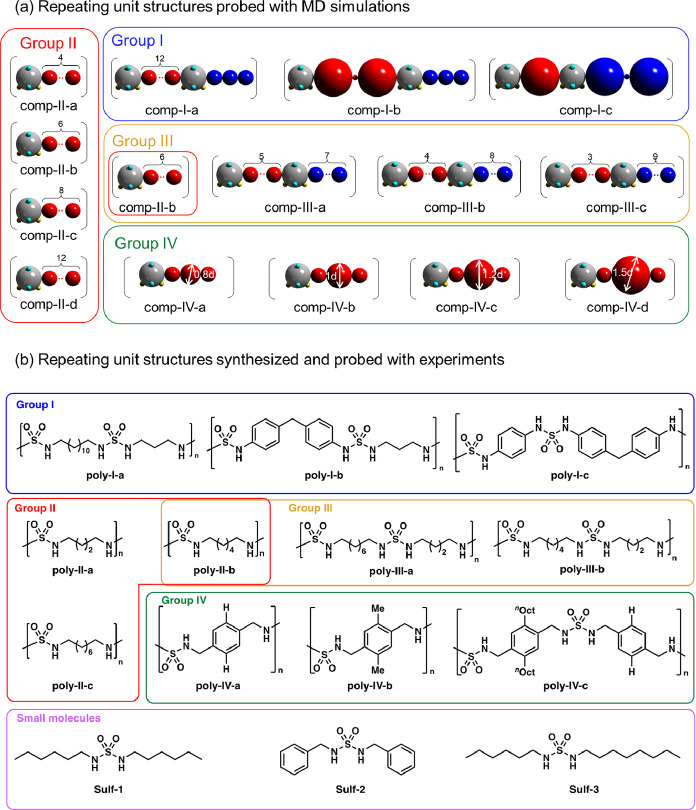
Polysulfamides and *N*,*N*′-disubstituted sulfamides studied in this work (a) using MD simulations and (b) in experiments. For improved visualization of the segments in simulation configurations (later in the manuscript), we use in part (a) red and blue colors to represent backbone segments between sulfamide beads when the polysulfamide backbone consists of two alternating chemical structures (those in **comp-I** and **comp-III**) and use only red color to represent backbone spacers when the backbone consists of only one chemical structure (**comp-II** and **comp-IV**). A guide for the readers who are comparing the CG model representation of the molecules in (a) with their analogous chemical structures in (b): for aliphatic segments between sulfamide groups in group I and II polymers, the number of CG beads is equal to the number of CH_2_ groups in the repeating unit; for example, in group II polymers, **comp-II-a** has four red CG beads consistent with the four CH_2_ in **poly-II-a**.

In Figure 2, we list all the different polysulfamides probed in this work using CGMD simulations and in experiments.

The polysulfamides modeled and studied with MD simulations (Figure 2a) are combinations of the three types of repeating units shown in Figure 1c and are categorized into four groups. Polysulfamides in group **comp-I** (i.e., polymers **comp-I-a** to **comp-I-c**) have been previously synthesized and characterized,^17^ and the simulation results are compared to experimental results as a validation of our computational approach. Groups **comp-II** and **comp-III** were designed to study the influence of generic structural features: length of aliphatic repeating unit (group **comp-II**) and uniformity in subunit length (group **comp-III**). Of note, **comp-II-b** belongs to both **comp-II** and **comp-III** groups. Group **comp-IV** allowed us to investigate the role of bulkiness and contains polymers with aromatic groups of varying sizes separated by a small methylene group.

A variety of polysulfamides were synthesized (Figure 2b) to provide an experimental comparison to the polymers modeled in groups **comp-I–IV**. The corresponding experimental groups are referred to as **poly-I** to **poly-IV**. The labeling of synthesized polymers matches the labeling of the computational polymers to show the reader the correspondence between synthesized polymer groups and design variations in computational groups. Polymers in group **poly-I** have been reported previously,^17^ whereas polymers in groups **poly-II**, -**III**, and -**IV** have been synthesized and characterized specifically for this study. Polysulfamides in group **poly-II** are homopolymers with aliphatic repeating units of different lengths. Polysulfamides in group **poly-III** contain two alternating aliphatic subunits with different numbers of methylenes (e.g., different length); polymers in group **poly-IV** are based on three α,α’-paraxylyl monomers with increasing bulkiness through substitution of the aryl groups with methyl or *n-*octyl side chains.

## 3 Results and Discussion

### 3.1 Experimental Characterization of Crystallization and H-Bonding in Polysulfamides

**Figure 3:**
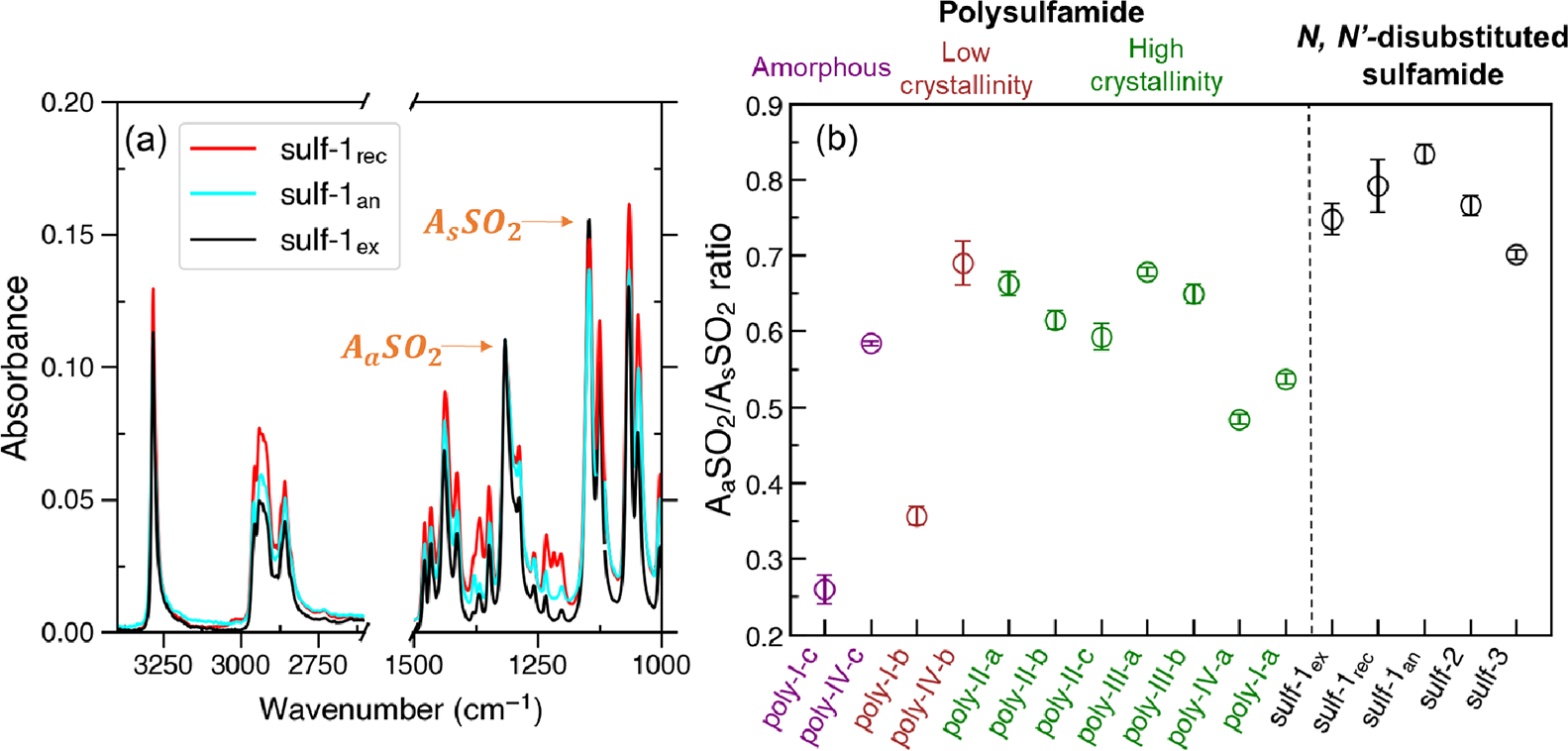
(a) IR spectra for *N*,*N*′-dihexylsulfamide after extraction (**sulf-1_ex_**), after recrystallization (**sulf-1_rec_**), and after annealing (**sulf-1_an_**). Spectra were normalized at the absorbance of the S=O asymmetric stretch (A_a_SO_2_) to facilitate visual comparison. (b) A_a_SO_2_/A_s_SO_2_ ratio determined from the FTIR analysis of all synthesized polysulfamides and *N*,*N*′-disubstituted sulfamides.

We applied FTIR and PXRD in a complementary manner to determine structural features that can be compared to computational analysis. Our primary analytical method for the determination of the crystallinity of polysulfamide is PXRD. We also applied FTIR as a complementary method to compare the extent of order within the assembled structure of polysulfamide as the lack of polysulfamide standards precluded quantitative determination of crystallinity via PXRD. Inspired by the FTIR analysis of *N*,*N′*-disubstituted sulfamides in powder form or thin films by Lucazeau and co-workers,^107,108^ we surmised that FTIR could provide a practical method to investigate the degree of self-assembly of polysulfamides. Indeed, Lucazeau and co-workers found that an increase in molecular organization of the sulfamides through hydrogen bonding led to a stark decrease of the intensity of the S=O symmetric stretch (A_s_SO_2_) at ∼1145 cm^–1^, whereas the absorption intensity of the S=O asymmetric stretch (A_a_SO_2_) at ∼1315 cm^–1^ remained unaffected.^106,107^ To test our hypothesis, we synthesized *N*,*N*′-dihexylsulfamide (**sulf-1**) and collected FTIR spectra of the product after extraction (**sulf-1_ex_**), after purification through recrystallization (**sulf-1_rec_**), and after annealing (**sulf-1_an_**) (Figure 3a). Comparison of the three spectra revealed an increase of the A_a_SO_2_/A_s_SO_2_ ratio after recrystallization and after annealing, consistent with Lucazeau and co-workers’ report. This observation prompted us to determine the A_a_SO_2_/A_s_SO_2_ ratio for all the synthesized polysulfamides (groups **poly-I** to **poly-IV**), as well as for two additional *N*,*N*′-disubstituted sulfamide derivatives (**sulf-2** and **sulf-3**). Figure 3b shows the A_a_SO_2_ ratio for all polysulfamides grouped by qualitative extent of crystallinity determined using PXRD and for sulfamides **sulf-1–3** grouped separately. When analyzed via PXRD, polymeric samples with higher crystallinity exhibit mostly sharp diffraction peaks, whereas amorphous samples only showcase one broad peak, often referred to as the amorphous halo. Intermediate samples display an amorphous halo combined with more or less sharp features (Figure S4).

We observed the following key trends: (i) *N,N′*-disubstituted sulfamides (**sulf-1** to **sulf-3**) that are known to be highly crystalline^2,3,109^ have the highest A_a_SO_2_/A_s_SO_2_ ratios. (ii) In general, as the crystallinity of polysulfamide decreases, the values of A_a_SO_2_/A_s_SO_2_ ratio also decrease; lower crystallinity polysulfamide have lower values of A_a_SO_2_/A_s_SO_2_ ratio (<0.4) as compared to their more crystalline counterparts (>0.5). These observations seem to suggest that the crystallinity qualitatively seen in PXRD and the value of A_a_SO_2_/A_s_SO_2_ ratio can together provide experimental insights into the degree of crystallinity. We note that there are indeed outliers to these trends as seen with **poly-IV-b**, **poly-IV-c**, and to a lower degree **poly-IV-a**. These discrepancies with the otherwise observed trend could result from larger structural differences due to the presence or absence of side chains complicating the IR analysis for these polymers. The crystallinity of polysulfamides in **group IV** could also be driven by other weak interactions including π–π interactions in addition to or in place of the H-bonding interactions putatively captured by the A_a_SO_2_/A_s_SO_2_ ratio. As we compare computational predictions and experimental observations for each group of polymers, we discuss in more detail these trends in Sections 3.3–3.5.

### 3.2 Validation of the Computational Approach through Comparison of Simulations and Experimental Data for Polysulfamides of Group I

**Figure 4:**
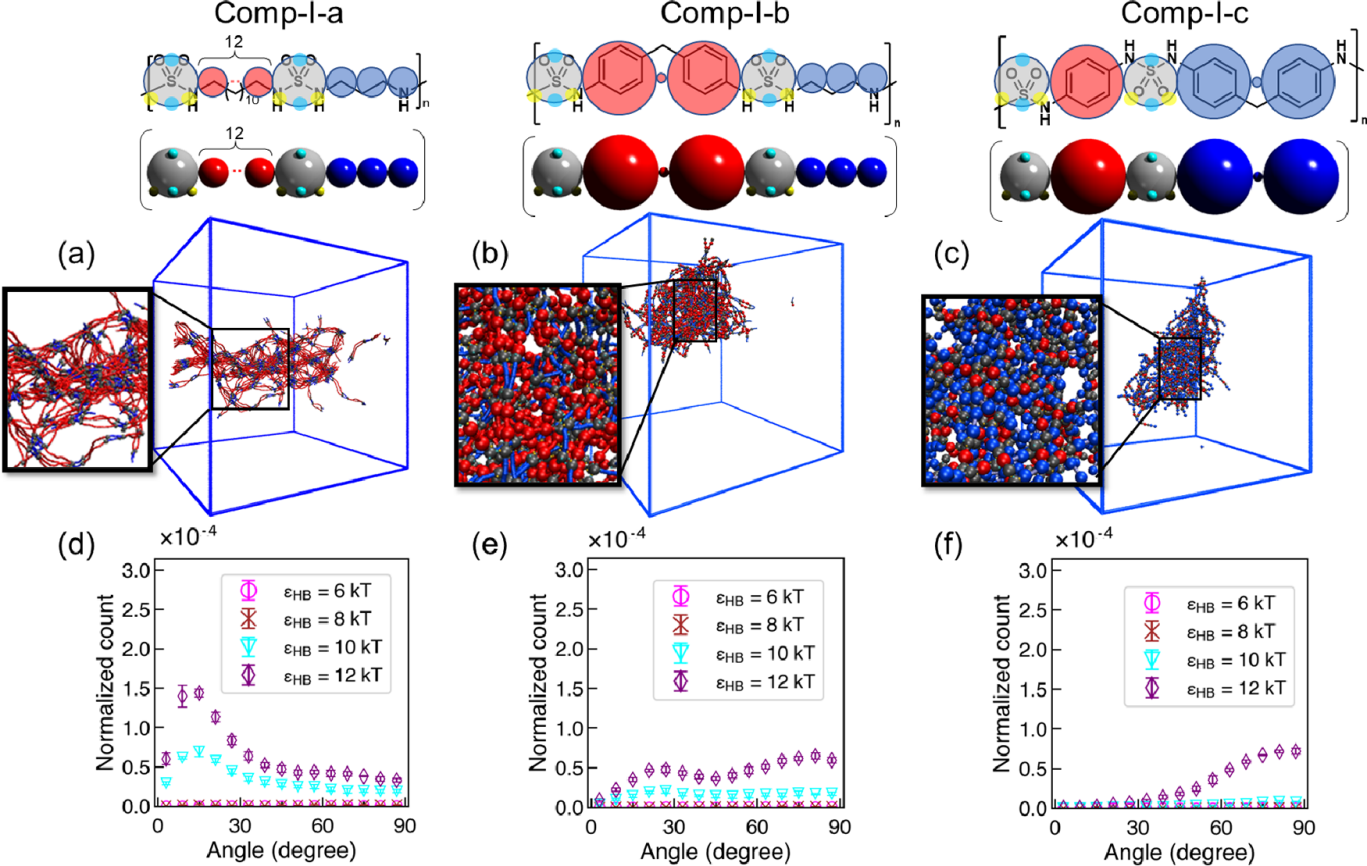
Results from CG MD simulations for the repeating units shown in group **comp-I** in Figure 2. (a–c) Visualizations of CG MD simulation configurations at ϵ_HB_ = 12 kT rendered using Visual Molecular Dynamics (VMD).^110^ Sulfamide beads in the polymer are represented in gray, and repeating units are in red and blue. (d–f) Distribution of intersegment angle between H-bonded segments (α_HB_) at ϵ_HB_ = 6, 8, 10, and 12 kT. Error bars indicate standard deviation between nine configurations from three independent trials; when the error bars are too small, they are not visible.

We first validate our computational approach by comparing computational to experimental results for previously synthesized^106^ polysulfamides (group **comp-I** and group **poly-I** in Figure 2). PXRD analysis revealed a range of crystallinity for **poly-I-a** to **poly–I-c**(17) from semicrystalline (**poly-I-a**) to amorphous (**poly-I-b** and **poly-I-c**, Figure S4). The visualizations of simulated configurations of analogous polymers (**comp-I-a** to **comp-I-c** in Figure 2a) at the highest H-bonding strength (ϵ_HB_= 12 kT) and intersegment angle distributions between H-bonded segments (α_HB_) for those three polymers are shown in Figure 4. From the visualizations, we can see that, in agreement with the experimental trend, assembly of **comp-I-a** (Figure 4a) exhibits the highest orientational order at ϵ_HB_= 12 kT among the three polymers. **Comp-I-a** chains assemble into fibrils with locally aligned polysulfamide chains; in contrast, no local orientational order can be identified in assemblies of **comp-I-b** and **comp-I-c** (Figure 4b,c). These observations are confirmed by the interchain angle (α_HB_) distribution calculation for **comp-I-a** to **comp-I-c**, as shown in Figure 4d–f. At ϵ_HB_ > 8 kT, we see peaks at low α_HB_ in the α_HB_ distribution for **comp-I-a**, indicating well-aligned neighboring chains and the emergence of orientational order; for **comp-I-b** and **comp-I-c**, the segments exhibit a broad range of α_HB_.

To compare their local positional order, we present the radial distribution function [*g*(*r*)] (Figure S5) between the sulfamide beads in the polymers at ϵ_HB_ = 12 kT for these three different backbones (**comp-I-a** to **comp-I-c**) in Figure 2a. In agreement with the experimental PXRD results (Figure S4) and computational results of orientational order (Figure 4), we see a significant *g*(*r*) peak at *r* < 4*d* for **comp-I-a** with diminishing values of *g*(*r*) at higher length scales; this indicates short-range positional order corresponding to the interconnected, locally aligned fibrils present in Figure 4a. In contrast, for **comp-I-b** and **comp-I-c**, the peak values of *g*(*r*) are much lower, indicating low positional order compared to chemistry **comp-I-a**.

All these results suggest that, in accordance with experimentally observed semicrystallinity seen only for **comp-I-a** and mostly amorphous structures seen for **comp-I-b** and **comp-I-c**, the CG MD simulations also exhibit significantly lower orientational and positional order in simulations for **comp-I-b** and **comp-I-c** compared to **comp-I-a**.

**Figure 5:**
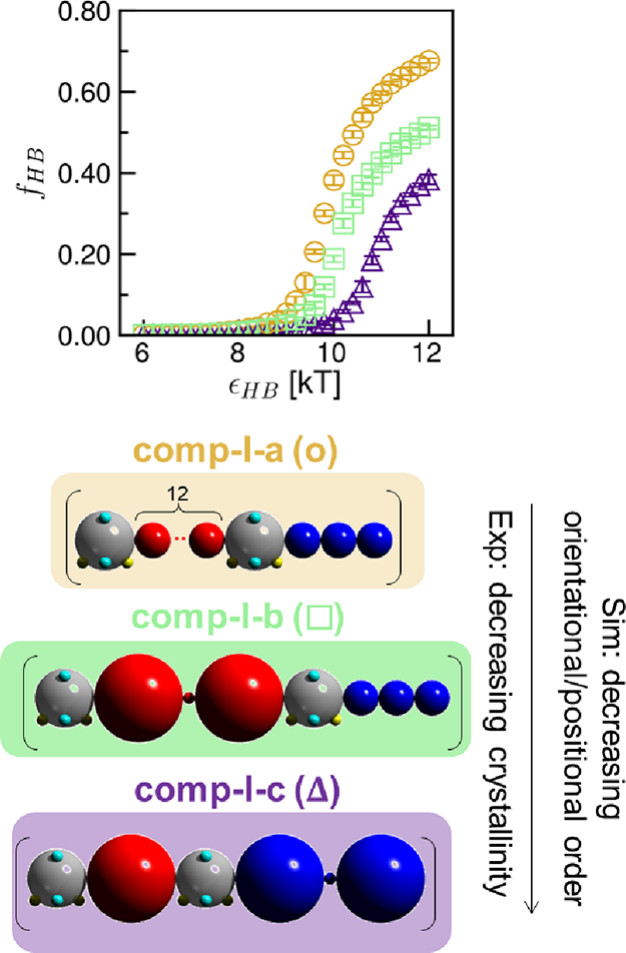
Hydrogen bonding propensity (*f*_HB_) for group **comp-I** in Figure 2 at ϵ_HB_ = 6–12 kT. Error bars indicate standard deviation between nine configurations from the three independent simulation trials.

We show the H-bonding propensity (*f*_HB_) between polysulfamide chains for these **comp-I** polymers in Figure 5. We find that the polymer with higher *f*_HB_ in simulations in most cases exhibits higher semicrystallinity (experimentally) and orientational/positional order (computationally) in the assembled structures formed. The results suggest that the bulkiness of the repeating units in the polymers likely hinders the formation of directional H-bonding interactions between sulfamide beads and reduces orientational and positional order. For example, in both **comp-I-b** and **comp-I-c**, at least one of the two repeating units contains a bulky group (i.e., benzene ring) next to the sulfamide bead, and these bulky groups hinder H-bonding interactions. In contrast, in **comp-I-a**, there is no bulky group in the repeating units, and H-bonding donor and acceptor beads in the sulfamide groups face no hindrance in forming H-bonds.

Thus far, CG MD simulations with **comp-I-a** to **comp-I-c** polymers qualitatively reproduce experimental trend in semicrystallinity in synthesized polysulfamides **poly-I-a** to **poly-I-c**. These CG MD simulation results also suggest that the repeating unit bulkiness could hinder H-bond formation between sulfamide beads, lowering the orientational/positional order of assembly for such polymers.

The results presented in this section serve as a *qualitative* validation of the CG MD computational approach to predict orientational and positional order in polysulfamides as a function of the design of the main chain prior to their syntheses.

### 3.3 Varying Length of the Aliphatic Repeating Units

Next, focusing on the effect of the contour lengths of substituting aliphatic chains on the orientational and positional order in the assembled polysulfamide chain, we discuss the structural predictions for the **comp-II-a** to **comp-II-d** polymers (Figure 2a). For these **comp-II-a** to **comp-II-d** polymers, we used small CG beads to mimic the alkyl groups. The repeating units in **comp-II-a** to **comp-II-d** are analogous to butyl (C4-C4), hexyl (C6-C6), octyl (C8-C8,) and dodecyl (C12-C12) repeating units, respectively.

**Figure 6:**
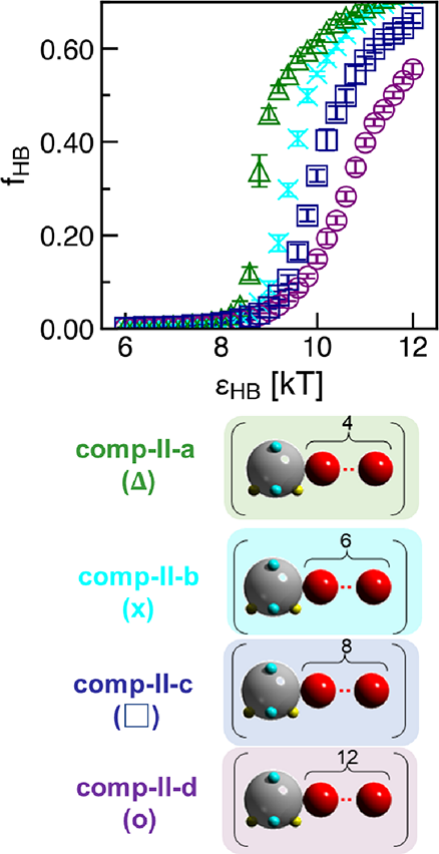
Hydrogen bonding propensity (*f*_HB_) for structures in computational polymers **comp-II-a** to **comp-II-d** at ϵ_HB_ = 6–12 kT. Error bars indicate standard deviations between nine configurations from three independent simulation trials.

We present the H-bonding propensities (*f*_HB_’s) for each polymer system at different H-bonding strengths (ϵ_HB_) in Figure 6. We see that the *f*_HB_’s of all four polymers, **comp-II-a** to **comp-II-d** (Figure 6), are significantly higher than those of **comp-I-b** and **comp-I-c** (Figure 5). Further, the *f*_HB_’s at all ϵ_HB_ values increase as the contour length of the repeating units in **comp-II** polymers decreases. **Comp-II-a** (analogous to **poly-II-a**), with the shortest contour length of substituting aliphatic chain, has the highest *f*_HB_, and **comp-II-d**, with the longest contour length of the aliphatic chain, has the lowest *f*_HB_.

**Figure 7:**
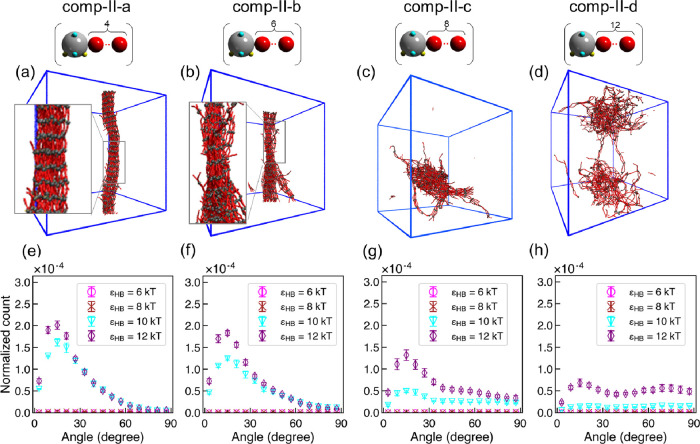
Key results from CG MD simulations of polysulfamides **comp-II-a** to **comp-II-d** shown in Figure 2. (a–d) Visualization of CG MD simulation configurations at ϵ_HB_ = 12 kT. Sulfamide beads are represented in gray, and repeating units are in red and blue. Configurations rendered using Visual Molecular Dynamic (VMD).^110^ (e–h) Distribution of intersegment angle between H-bonded segments (α_HB_) at ϵ_HB_ = 6, 8, 10, and 12 kT. Error bars represent standard deviations between nine configurations from three independent simulation trials.

On the basis of our identified correlation between H-bonding propensity and orientational/positional order in Section 3.2, going from **comp-II-a** to **comp-II-d,** we expect to see decreasing orientational and positional order. This is confirmed in Figure 7 where we show the visualizations of our simulated configurations at the highest H-bonding strength (ϵ_HB_= 12 kT) and intersegment angle distributions between H-bonded segments (α_HB_) for **comp-II-a** to **comp-II-d**. Indeed, Figure 7a shows a fibrillar assembly for **comp-II-a** (analogous to **poly-II-a**) exhibiting high orientational order, with the inset showing the polysulfamide beads (gray) aggregating into distinct H-bonding planes exhibiting high positional order. This high orientational order is quantitatively proven in Figure 7e, where a substantial majority of the H-bonded polysulfamide segments exhibit alignment, sampling intersegment angle (α_HB_) values that are less than 30°. Likewise, the high positional order is quantitatively proven by the radial distribution function [*g*(*r*)] in Figure S6e, with multiple consecutive pair correlation peaks at high peak values. Those quantitative signatures of high orientational and positional order gradually disappear with increasing contour lengths of the repeating units in **comp-II-a** to **comp-II-d** polymers. For **comp-II-d**, the assembled structure appears to be amorphous in the visualization (Figure 7d), with low α_HB_ angles no longer preferred (Figure 7h) and secondary and further peaks in the *g*(*r*) disappearing (Figure S6h), thereby confirming reduced orientational and positional order in the assembly.

The results for **comp-II-a** to **comp-II-d** shown in Figure 7 and Figure S6 confirm our expectation that increasing alkyl chain lengths in the repeating units leads to decreased H-bonding propensity and reduced orientational and positional order in the assembled structures. We explain this trend as follows: As the alkyl chain length in the repeating units increases, we expect that the conformational entropy loss upon formation of H-bonds between sulfamide groups should also increase, resulting in ordered configurations being less energetically favorable. This reasoning is analogous to findings from recent studies by Cooper *et al.*(111) that flexible polymer chains with “stickers” distributed along the chain transition from an ordered nanofiber structure to an amorphous structure as the length between “stickers” increases. We note that the orientational order and positional order of **comp-II-d**, the least ordered structure among the **comp-II** polymers, are still significantly higher than those of **comp-I-b** and **comp-I-c** (Figure 4 and Figure S5), and similarly, *f*_HB_ of **comp-II-d** is also significantly higher than those of **comp-I-b** and **comp-I-c** (Figure 5). These results further strengthen the argument that orientational and positional orders of the assembly of polysulfamides are correlated with *f*_HB_ in our CG MD simulation results.

The CG MD simulations of **comp-II** polysulfamides suggest that longer aliphatic subunits lead to less orientational and positional order in the assembled structure, which translates to lower crystallinity in experiments. However, the effect of aliphatic chain length should be less significant than the bulkiness of the subunits. In other words, for the above repeating unit length to alter the extent of orientational and positional order in the assembled structure, the repeating units should not be bulky. To test the above computational predictions, we synthesized and characterized the polysulfamides in experimental group **poly-II** (**poly-II-a** to **poly-II-c**) via NMR and IR spectroscopies, as well as SEC, PXRD, TGA, and DSC (Figure 2b and the Supporting Information).

**Figure 8:**
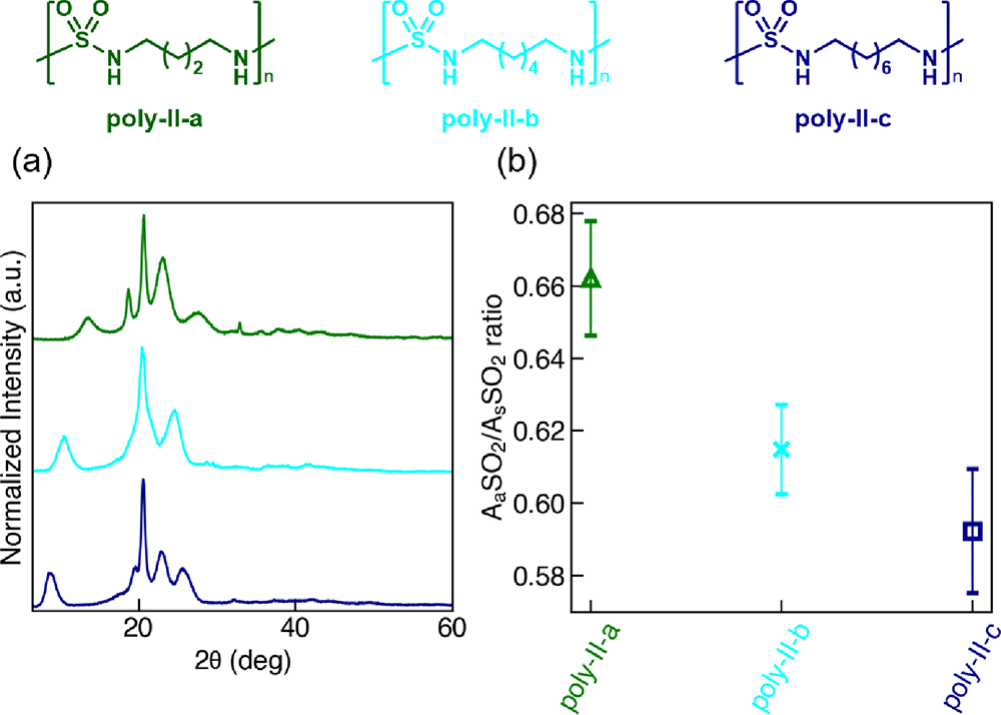
The experimental characterization results of the polysulfamide self-assembly in experimental group **poly-II**. (a) PXRD patterns and (b) A_a_SO_2_/A_s_SO_2_ ratio from FTIR analysis of **poly-II-a, poly-II-b,** and **poly-II-c**. Error bars in (b) indicate standard deviations from four repeated FTIR measurements between two batches of synthesized polysulfamides.

The three polysulfamides **poly-II-a** to **poly-II-c** have increasing length of the alkyl repeating unit (butyl, hexyl, and octyl) that mirrors **comp-II-a** to **comp-II-c**. Because all three polysulfamides exhibit a semicrystalline behavior from PXRD (Figure 8a), we refer to the A_a_SO_2_/A_s_SO_2_ ratio of the S=O bond measured using FTIR (Figure 8b) for crystallinity. As discussed in Section 3.1., in most cases (without bulky groups), a decrease in crystallinity corresponds to a decrease in A_a_SO_2_/A_s_SO_2_ ratio. In Figure 8b, we observe decreasing A_a_SO_2_/A_s_SO_2_ ratio with increasing length of alkyl side chains, which suggests that the propensity of polysulfamide chains to assemble decreases with increasing length of aliphatic repeating units, in agreement with the CG MD simulation predictions.

To summarize this section, both experiments and simulations show that polysulfamides with only aliphatic chains as repeating units form semicrystalline assembled structures, with shorter aliphatic repeating units exhibiting quantitatively higher orientational and positional order than those with longer repeating units.

### 3.4 Varying (Non)Uniformity in Lengths of Repeating Units on either Side of the Sulfamide Group

In all the polymers used in **comp-III**, we kept the total number of alkyl carbons in a repeating unit equal to 12 but varied the alkyl chain lengths of the repeating units on either side of the sulfamide group to study the effect of length variation between the two repeating units on the orientational and positional order of the polymer assembly. We note that **comp-II-b** serves the purpose of uniform “base case” for the series **comp-III-a** to **comp-III-c** polymers that exhibit increasing nonuniformity.

**Figure 9:**
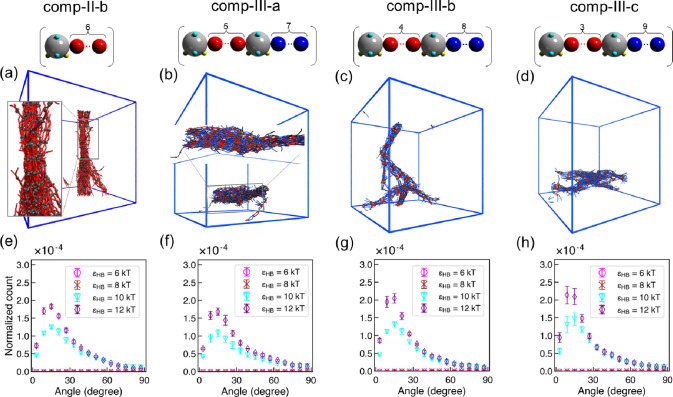
Key results from CG MD simulations of various polysulfamides in group **comp-III**. (a–d) Visualization of CG MD simulation configurations at ϵ_HB_ = 12 kT. Sulfamide beads in the polymers are represented in gray, and repeating units are in red and blue. Configurations rendered using Visual Molecular Dynamics (VMD).^110^ The choice of red and blue colors for the segments that are otherwise chemically similar is purely for clearer visualization of the short and long segments. (e–h) Distribution of intersegment angle between H-bonded segments (α_HB_) at ϵ_HB_ = 6, 8, 10, and 12 kT. Error bars represent standard deviations between nine configurations from three independent simulation trials.

In Figure S7, we see that *f*_HB_ values are similar for the four cases (**comp-II-b** and **comp-III-a** to **comp-III-c**) across all H-bonding strengths. In Figure 9, we show the representative visualizations of our simulated configurations at the highest H-bonding strength (ϵ_HB_= 12 kT) and the intersegment angle (α_HB_) distributions between H-bonded segments for those four computed polysulfamides at various H-bonding interaction strengths. The visualizations and the α_HB_ distributions are similar for the four polymers, confirming similar orientational order in the assembled structure. Figure S8 shows the radial distribution function [*g*(*r*)] of the four computed polysulfamides, where repeating units with increased length variation between the two types of repeating units group exhibit a more prominent primary peak (at *r* ≈ 1*d*) but less prominent tertiary peak (at *r* ≈ 5*d*), indicating an increase in short-range but decrease in long-range positional order with increasing nonuniformity. The explanation for this trend is as follows: when the repeating unit alkyl chains have a high variation in length, for maximizing enthalpically favorable H-bonding interactions between two neighboring chains, shorter repeating units have to be aligned with the neighboring shorter repeating units (e.g., for **comp-III-c**, C3 with C3) and longer repeating units with the neighboring longer repeating units (e.g., for **comp-III-c**, C9 with C9). In contrast, when the repeating units are uniform in length, such a configurational restraint is not necessary. Having configurational restraints leads to entropic losses (due to a smaller number of configurations that satisfy that restraint). Thus, with increasing variation in repeating unit lengths on either side of the sulfamide group, because of higher entropic losses, the system should have a (slightly) larger free energy barrier for attaining positional order as compared to the uniform repeating units on either side of the sulfamide group.

In Figure S9, we show the results of an extended study related to group **comp-III**, in which we further increase the degree of nonuniformity beyond **comp-III-c** and test a “**comp-III-d**” with C2 and C10 on either side of the sulfamide bead. Figure S9a shows that compared to **comp-II-b** that has uniform aliphatic repeating units, for **comp-III-d**, we observe hydrogen bonds forming at lower ϵ_HB_. At the highest ϵ_HB_, however, the hydrogen bonding propensity is similar for **comp-II-b** and **comp-III-d**. Following a similar trend, Figure S9b shows that although **comp-III-d** exhibits assembly at a lower ϵ_HB_ than **comp-II-b**, when the ϵ_HB_ (at 12 kT) is strong enough, assembly of **comp-III-d** exhibits similar ordering as **comp-II-b**. Quantitatively, there is no significant difference between the orientational order (Figure S9c) and positional order (Figure S9d) of **comp-II-b** and **comp-III-d**. These results suggest that at high nonuniformity (like **comp-III-d**) in repeating units, the hydrogen bonding strength needed to assemble the chains is reduced. This reduced energetic gain needed to drive assembly could be due to the lower entropic loss for the shorter repeating units (e.g., the C2 segment in **comp-III-d**) to align. Such a difference becomes insignificant, however, when the ϵ_HB_ is high enough that the energetic gain dominates over the entropic loss and (non)uniformity of repeating unit length does not have a noticeable effect on morphology of assembly.

In short, our simulations show minimal effects of repeating unit length variation on the orientational order of the assembly and slightly improved short-range positional order with increasing nonuniformity in segment lengths on either side of sulfamide. Next, we see whether the experiments capture similar/different effects in crystallinity with varying uniformity in repeating unit groups.

**Figure 10:**
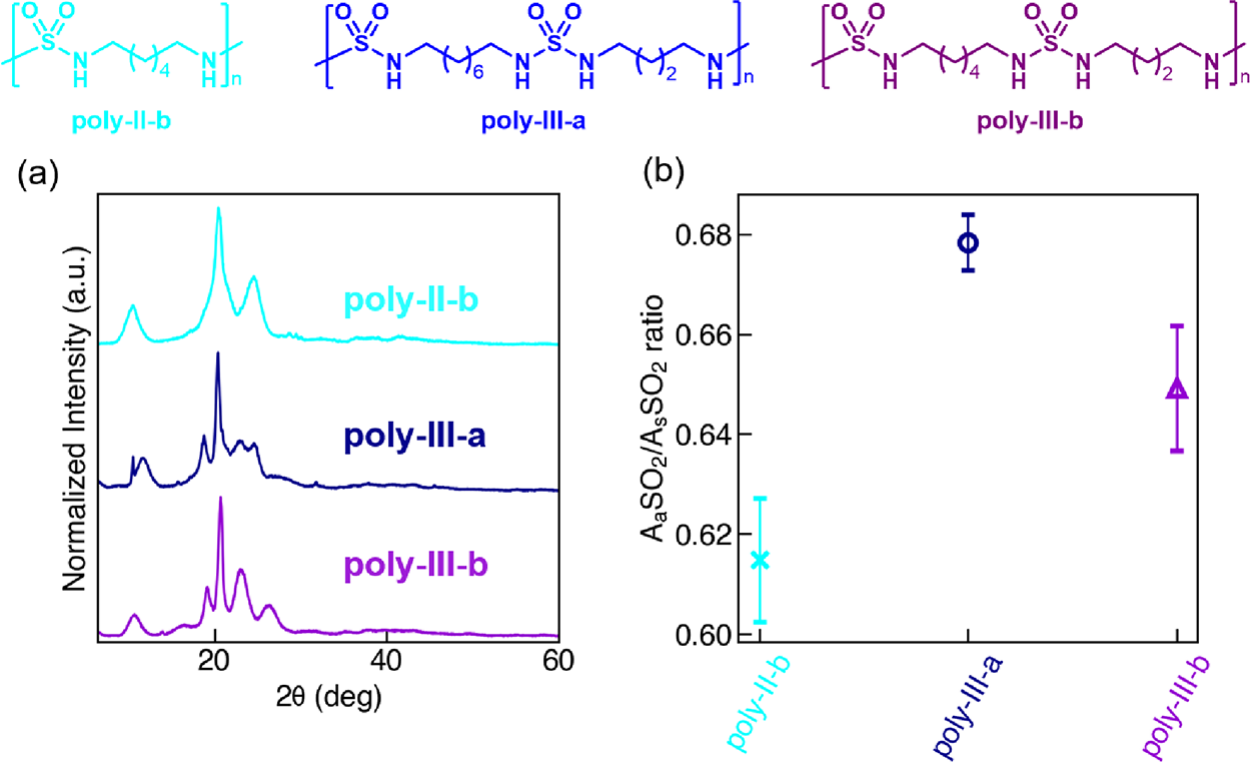
The experimental characterization results of the polysulfamide self-assembly in experimental group III. (a) PXRD patterns and (b) A_a_SO_2_/A_s_SO_2_ ratio from FTIR analysis of **poly-II-b**, **poly-III-a**, and **poly-III-b**. Error bars in (b) indicate standard deviations from four repeated FTIR measurements between two batches of synthesized polysulfamides.

We synthesized and characterized **poly-II-b**, **poly-III-a**, and **poly-III-b** (Figure 2) to study the effect of nonuniformity in the length of aliphatic repeating units on polysulfamide self-assembly. These three aliphatic polysulfamides are also found to be all semicrystalline based on PXRD (Figure 10a), so we refer to the FTIR analysis for quantitative comparison. The A_a_SO_2_/A_s_SO_2_ ratio of the S=O bond was calculated for each polymer using FTIR (Figure 10b). Both butyl/octyl alternating **poly-III-a** (corresponding to **comp-III-b**) and butyl/hexyl alternating **poly-III-b** were found with a slightly larger A_a_SO_2_/A_s_SO_2_ ratio than hexyl homopolymer **poly-II-b** (corresponding to **comp-II-b**), with the A_a_SO_2_/A_s_SO_2_ ratio of both **poly-III-a** and **poly-III-b** roughly on par with **poly-II-a** (Figures 3 and 8). The FTIR results seem to suggest that the two copolymers containing nonuniform aliphatic linkers lead to slightly higher crystallinity, which agrees with improved short-range positional order in simulations; however, it seemingly does not agree with the orientation order trends seen in CG MD simulation results (Figure 9). This disagreement in orientational order trends could be because the CG MD simulations only capture orientational ordering of chains but do not cross the energy barrier to nucleate crystalline domains in the semicrystalline morphology observed in experiments. Further, the CG model is “coarse” (i.e., loses atomistic resolution) and has not been optimized to capture the correct flexibility of alkyl chains as a function of number of carbons, which in turn could also contribute to differences between simulations and experiments. Despite this difference, the simulations and experiments do agree that all four polysulfamides in **group III**, regardless of the uniformity/nonuniformity in length, show higher orientational and positional order compared to repeating units with bulky groups in repeating units (e.g., **comp/poly-I-b** and **comp/poly-I-c**).

### 3.5 Effect of Side Chain Bulkiness on the Polysulfamide Assembly

In this section, we demonstrate the effect of additional alkyl side chains (methyl for **poly-IV-b** and octyl for **poly-IV-c**) in the α,α’-paraxylyl groups as the repeating units on the assembled structure; corresponding **poly-IV** chemical structures are shown in Figure 2b. The additional alkyl side chains on the α,α’-paraxylyl group are postulated to increase the bulkiness of the CG beads as shown in **comp-IV-a** to **comp-IV-d**.

**Figure 11:**
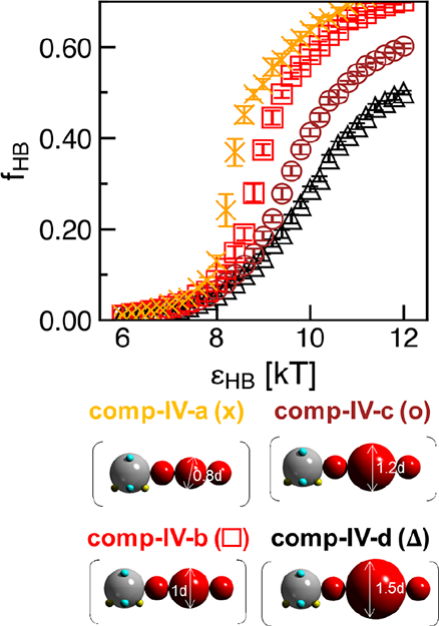
Hydrogen bonding propensity (*f*_HB_) for polysulfamides in computational polymers **comp-IV-a** to **comp-IV-d** at ϵ_HB_ = 6–12 kT. Corresponding group **poly-IV** chemical structures are in Figure 2b. Error bars indicate standard deviation between nine configurations from three independent simulation trials.

**Figure 12:**
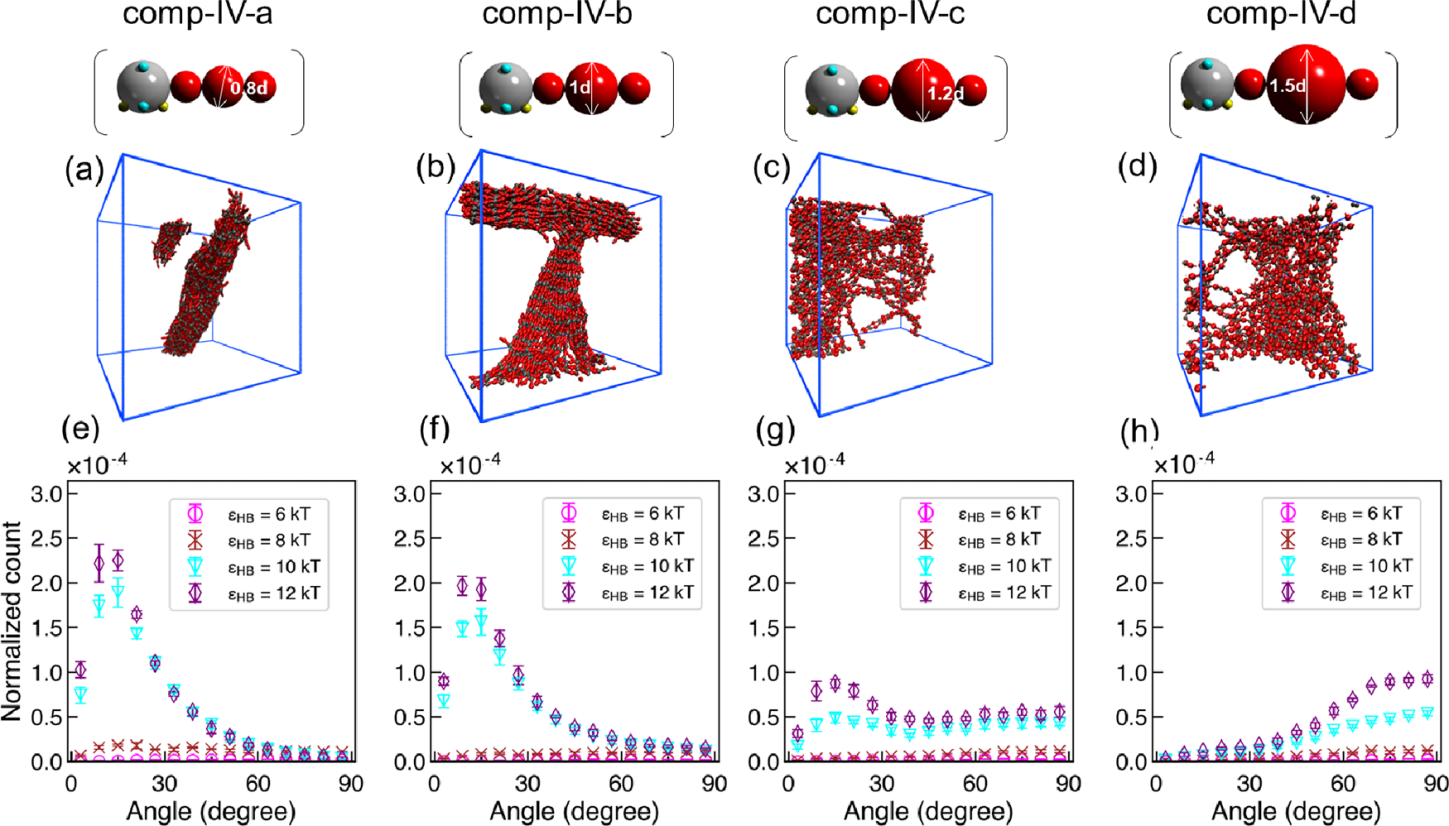
Key results from CG MD simulations of polysulfamides **comp-IV-a** to **comp-IV-d**. (a–d) Visualization of CG MD simulation configurations at ϵ_HB_ = 12 kT. Sulfamide beads are represented in gray, and repeating units are in red and blue. Configurations rendered using Visual Molecular Dynamics (VMD).^110^ (e–h) Distribution of intersegment angle between H-bonded segments (α_HB_) at ϵ_HB_ = 6, 8, 10, and 12 kT. Error bars represent standard deviations between nine configurations from three independent simulation trials.

We have shown in Section 3.2 that the bulkiness of repeating units in general hinders H-bonding formation and results in a mostly amorphous polymer assembly. With the α,α’-paraxylyl group in the repeating unit, the effect of bulkiness is further complicated by the presence of a methylene between the bulky aromatic core and the sulfamides. We model this repeating unit with one bulky bead between two small beads representing the CH_2_ groups (**comp-IV-a** to **comp-IV-d**). We vary the diameters of the bulky group bead with respect to the small beads modeling the −CH_2_– in the repeating unit from 0.8*d* to 1.5*d*. Figure 11 shows the resulting H-bonding propensity (*f*_HB_) as a result of varying this bulky CG bead size. As the size of the middle bulky bead in the repeating unit increases, *f*_HB_ at all H-bonding strength decreases. In Figure 12, as the middle bulky bead in the repeating unit becomes larger, there is a clear decrease in orientational order both in the visualizations with the emergence of aligned bundles (Figure 12a–d) and in α_HB_ distribution with the shift of peaks from low to high angles (Figure 12e–h). Likewise, in Figure S10, as the size of the middle bulky bead in the repeating unit increases, the value of the primary contact peak of the *g*(*r*) clearly decreases, and secondary and further peaks disappear, indicating lower positional order with larger middle bulky bead. On the basis of these trends, we expect that polysulfamides with additional alkyl side chains on the α,α’-paraxylyl groups should assemble into structures of less orientational and positional order, or lower crystallinity in experiments, than its counterparts without additional alkyl side chains.

**Figure 13:**
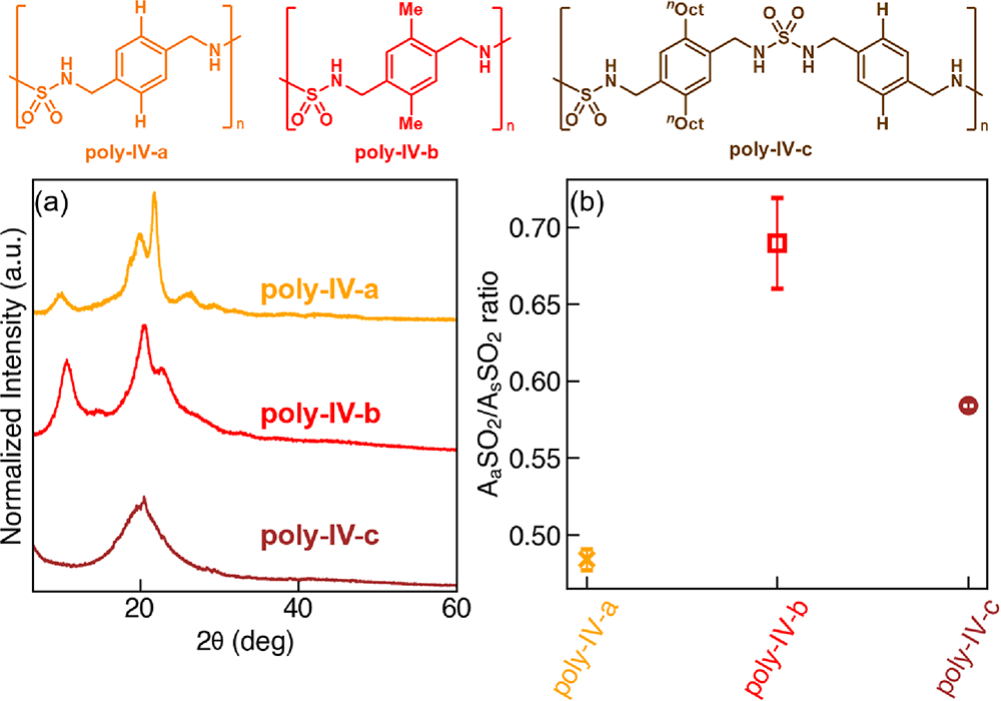
The experimental characterization results of the polysulfamide self-assembly in experimental group IV. (a) PXRD patterns and (b) A_a_SO_2_/A_s_SO_2_ ratio from FTIR analysis of **poly-IV-a** to **poly-IV-c**. Error bars in (b) indicate standard deviations from four repeated FTIR measurements between two batches of synthesized polysulfamides.

To test the computational predictions, we synthesized **poly-IV-a** to **poly-IV-c** and characterized their crystallinity through PXRD. Of note, **poly-IV-c** was designed with a backbone alternating between α,α’-paraxylyl and dioctyl-substituted α,α’-paraxylyl groups because the dioctyl-substituted α,α’-paraxylyl homopolymer was found to have a waxy appearance that precluded PXRD characterization. We thus prepared **poly-IV-c** instead, with alternating dioctyl/hydrogen α,α’-paraxylyl groups. From the PXRD measurement alone (Figure 13), we see a clear trend that the polymer with longer side chains exhibits lower crystallinity in the assembled structure. **Poly-IV-c** with octyl chains on one side is amorphous, whereas **poly-IV-b** with methyl chains and **poly-IV-a** without side chains are both semicrystalline; the Bragg’s peak of **poly-IV-b** is broader than that of **poly-IV-a** and contains a larger amorphous halo, indicating a less ordered polymer chain alignment.

We note that although the FTIR results for **poly-IV-a** to **poly-IV-c** (Figure 3) show a significantly higher A_a_SO_2_/A_s_SO_2_ ratio for **poly-IV-b** and **poly-IV-c** than **poly-IV-a**, this result should not be used as contradicting evidence that suggests that **poly-IV-b** and **poly-IV-c** samples have higher extent of crystallinity than **poly-IV-a**. In Sections 3.3 and 3.4, the repeating units of polysulfamide are similar (consisting only of aliphatic chains of various lengths) and result in presumably similar IR bands. We were thus able to attribute the change in IR intensity to only the change in crystallinity. In this section, the side chains significantly alter the chemical structure of the repeating units, which potentially leads to different combinations of modes of vibration and complicates the IR analysis. Further studies are needed to refine the IR predictions of crystallinity for polysulfamides with more substantial structural variations. Although qualitative without available standards, PXRD is a method that allows one to directly probe the crystallinity of polymeric samples. For polysulfamides in **group IV**, PXRD alone unambiguously shows that **poly-IV-a** is more crystalline than **poly-IV-b** and **poly-IV-c**. Therefore, we do not need to use FTIR as a supplementary measurement to differentiate the crystallinity. The collected IR data nonetheless suggest that our simple IR analysis is currently limited in its ability to capture the change in crystallinity convoluted with differences in molecular-level interactions. This method is thus far more suitable for the comparison of polymers bearing high structural similarity and will require further refinement in future studies.

In summary, in this section, the experimental characterization of the polymers in the **poly-IV** group supports the prediction by our CG MD simulation and suggests that the less bulky polymer backbone resulting from shorter alkyl side chains on the benzene enhances the positional and orientational order in the polysulfamide self-assembly.

## 4 Conclusions

We developed a coarse-grained (CG) model to investigate the relationship between the design of polysulfamides (bulkiness, length, and uniformity in length in repeating units) and their orientational and positional order upon assembly driven by H-bonding. Upon validation of our CG model and MD simulation protocol through the reproduction of structural trends across three previously synthesized polysulfamides, we applied our computational approach to predict how structural modifications of the backbone affect the orientational and positional order. These predictions were then tested by experiments with synthesis and characterization of new polysulfamides. Through simulations and experiments, we arrived at the following design rules:

1. Increasing the bulkiness of the subunits on either side of the sulfamide group (e.g., by using aromatic groups) hinders H-bond formation and results in amorphous aggregates (in simulations) and lower crystallinity (in experiments).

2. For less bulky repeating units (e.g., with aliphatic segments), decreasing the contour length of the repeating units increases H-bond formation and improves the orientational and positional order within the chain aggregates in simulations; this is confirmed with increasing crystallinity in experiments.

3. Increasing the nonuniformity in lengths of repeating units does not alter the orientational order of the assembly of polysulfamide chains but increases the positional order at short range in simulations.

We note that the design rules provided here are not exhaustive and that there are other factors that could further influence the measured crystallinity and the computed orientational and positional order of the assembly. To name a few, we have not tested the effect of stiffness of the repeating units, which can correspond to the difference between, e.g., saturated vs unsaturated alkyl chains with similar contour length. Another factor that the CG model does not yet capture is the aromatic groups in the repeating units forming interchain contacts via directional π–π interactions serving as an additional driving force for polysulfamide assembly besides H-bonding interactions between sulfamide groups. These features will be the focus of a future investigation and will be reported in due time.

Lastly, the simulation protocol used in this study is not meant to mimic the polymer crystallization process even if the polymer has the propensity to crystallize. So, structures that exhibit a lack of order in simulations could be either low in crystallinity in experiments or also amorphous in experiments. Hence, we focused mainly on comparing qualitative trends seen in simulations and experiments throughout the paper. One of the future studies after this manuscript is to simulate with sophisticated (nontrivial) simulation protocols the process of polysulfamide crystallization. Similarly, in experiments, complementary methods to measure the semicrystallinity of polysulfamides will also be investigated soon to further refine our current IR analysis relying solely on the symmetric and asymmetric S=O stretches of the sulfamide group.

## Data Availability

The data presented in the results section are available on the open-access data repository Zenodo with DOI: 10.1021/acs.macromol.3c01093/zenodo. Additional computational and experimental details about the current study are available from the corresponding authors upon reasonable request.

## Supporting Information Available

Additional details on the CG model; additional details explaining the analyses of interchain angle distribution; additional experimental details on the synthesis and characterization of polysulfamides; and additional results from experiments and MD simulations (PDF)
