# Supplementary material for: Investigating the Hydrogen Bond-Induced Self-Assembly of Polysulfamides Using Molecular Simulations and Experiments
Source: Macromolecules. 2023 Jun 28;56(13):5033–49. doi: 10.1021/acs.macromol.3c01093 (PMC10865372; doi:10.1021/acs.macromol.3c01093)
Supplement: Supplementary file 1 — ma3c01093_si_001.pdf [file ma3c01093_si_001.pdf]

Submitted to JACS Au

Supporting Information

Investigating hydrogen bond-induced self-assembly of polysulfamides using molecular simulations and experiments

Zijie Wu[1], Jiun-Wei Wu[2], Quentin Michaudel[2*], and Arthi Jayaraman[1,3*]

[1]Department of Chemical and Biomolecular Engineering, University of Delaware, 150 Academy St., Newark, DE 19716

[2]Department of Chemistry, 580 Ross St., Texas A&M University, College Station, TX 77843

[3]Department of Materials Science and Engineering, University of Delaware, 201 DuPont Hall, Newark, DE 19716

* Corresponding authors

(A.J.) arthij@udel.edu,

(Q.M.) quentin.michaudel@chem.tamu.edu

Section S.I. Additional Coarse Grained (CG) Model Details

Table S1. Coordinates of coarse-grained (CG) beads in the CG model of sulfamide group

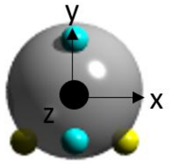

CG bead identity	Coordinate of bead center (in d)
**Sulfamide bead**	(0,0,0)
**Donor bead 1**	(0.37,-0.37,0)
**Donor bead 2**	(-0.37,-0.37,0)
Acceptor bead 1	(0,0.37,0.37)
Acceptor bead 2	(0,-0.37,0.37)

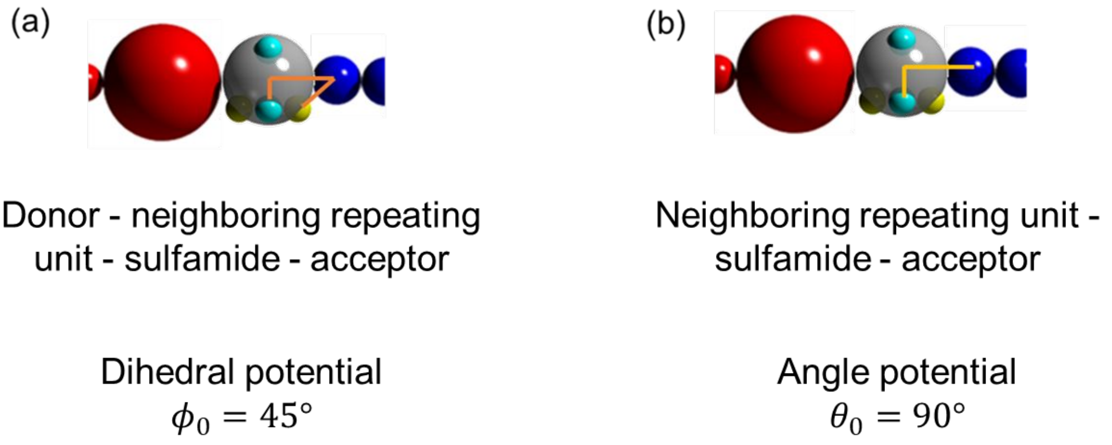

***Figure S1**. Additional angle and dihedral potentials restricting rotation of sulfamide bead with respect to neighboring repeating units.*

We apply additional angle and dihedral constraints to restrict the rotation of sulfamide bead around the bonds on either side of the sulfamide bead. These constraints also ensure that the donor and acceptor beads are at realistic orientation with respect to the neighboring beads in the repeating units. These constraints include a dihedral potential between the donor bead – sulfamide bead – one of the two neighboring repeating unit bead – acceptor bead (as shown in **Figure S1a**), represented as

$$U_{dihedral}(\phi) = k_{\phi}(\phi - \phi_0)^2$$

where $\phi_0 = 45°$ ($i.e., \frac{\pi}{4} rad$) and $k_\phi = 100\ kT/rad^2$, and an angle potential between one of the two neighboring repeating unit bead – sulfamide bead – acceptor bead (as shown in **Figure S1b**), represented as

$$U_{angle}(\theta) = k_\theta(\theta - \theta_0)^2$$

where $\theta_0 = 90°$ ($i.e., \frac{\pi}{2} rad$) and $k_\theta = 100\ kT/rad^2$.

Section S.II. Additional Information on Analyses of Inter-Segment Angle Between Hydrogen Bonded Segments

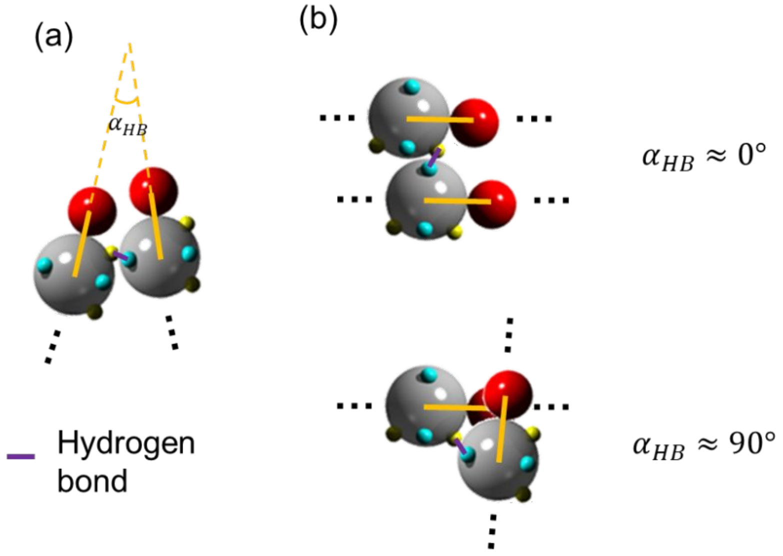

Figure S2. *(a) A schematic showing the definition of α_{HB} between two hydrogen-bonded segments. The yellow solid lines indicate sulfamide segments, and the cyan dotted line indicates hydrogen bond formed. (b) two example schematics showing cases of $\alpha_{HB} \approx 0°$ and 90°.*

Section S.III. Additional Experimental Procedures

General reagent information

All reactions were carried out under ambient atmosphere unless otherwise stated. Reagents including all bis(amine)s **S1**–**S4** were purchased at the highest commercial quality and used without further purification, unless otherwise stated. Yields refer to chromatographically (^{1}H NMR) homogeneous material, unless otherwise stated. Reactions were monitored by thin layer chromatography (TLC) carried out on 250 µm SiliCycle SiliaPlate™ silica plates (F254), using UV light as the visualizing agent, and an acidic solution of *p*-anisaldehyde with heat, ceric ammonium molybdate with heat, $KMnO_4$ with heat, or ninhydrin with heat, as developing agents. Flash silica gel chromatography was performed using SiliCycle SiliaFlash® Irregular Silica Gel (60 Å, particle size 40–063 µm). Polymers were isolated after precipitation using an Eppendorf Model 5804 centrifuge, and dried using a Shel lab Model 1410 vacuum oven.

General analytical information

Size-exclusion chromatography (SEC) of polymer samples was performed by EcoSEC Elite® HLC–8420 GPC with RI and either column TSKgel® SuperH3000/SuperH2500 columns sequence at a flow rate of 0.2 mL/min. *N*,*N*-Dimethylacetamide (DMAc) with 0.5% LiCl was used as the eluent. Number-average molecular weight (M_n), and dispersities (*Đ)* were calculated from refractive index chromatograms against poly(methyl methacrylate) standards.

Nuclear magnetic resonance (NMR): ^{1}H spectra were recorded on two Bruker Avance NEO 400 MHz and a Bruker Avance 500 MHz; ^{13}C spectra were recorded on a Bruker Avance 500 MHz; ^{19}F spectra were recorded using a Varian Inova 500 MHz instrument and calibrated by a solution of $CFCl_3$ in d_3-MeCN/$CDCl_3$ (@ 0 ppm ^{19}F NMR). All ^{1}H and ^{13}C spectra were calibrated using residual deuterated solvent as an internal reference ($CDCl_3$ @ 7.26 ppm ^{1}H NMR, 77.16 ppm ^{13}C NMR; d_3-MeCN @ 1.94 ppm ^{1}H NMR, 118.26 ppm ^{13}C NMR; d_6-DMSO @ 2.50 ppm ^{1}H NMR, 39.52 ppm ^{13}C NMR). The following abbreviations were used to explain NMR peak multiplicities: s = singlet, d = doublet, t = triplet, q = quartet, m = multiplet, br = broad.

Fourier transform–infrared (FT-IR) spectra were acquired using an Agilent Cary 630 FT-IR in the ATR-transmittance mode with a resolution of 2 cm^{-1} by averaging 16 scans in the range of

4000–500 cm^{-1}. The baseline passed through maxima in the transmittance spectrum that were located at the edges of the examined spectral range.

High-resolution mass spectra (HRMS) were recorded on an Agilent LC/MSD TOF mass spectrometer by electrospray ionization time-of-flight (ESI-TOF) reflection experiments unless otherwise told.

Powder X-Ray diffractions (PXRD) patterns were collected using Bruker D8 Endeavor diffractometer at room temperature (Cu-Kα radiation, fixed divergence slit 0.4 mm, sample to anti-air scatter slit distance of 2 mm, LynxEye XE-T detector and PSD opening of 4°) in the two theta range 3-70° with a step size and time of 0.03^{o} and 0.5 s respectively. Basic analysis were carried out using Bruker Diffrac.EVA software.

Melting points were recorded on a Fisher-Johns 13-144 melting point apparatus and are uncorrected.

Thermogravimetric analysis (TGA) was performed on a TA Instruments TGA 5500 Thermogravimetric Analyzer or an Instruments Q500 Analyzer. Typically, samples were heated at 10 °C/min to 600 °C under nitrogen. Data were processed using Universal Analysis 2000 for windows software.

Differential scanning calorimetry (DSC) was performed using a TA instruments DSC 2500. Samples were prepared in aluminum pans and were analyzed using the following heating program: −80 °C to 5 ºC below decomposition temperature at 10 °C/min, cooling to −80 °C at 10 °C/min, and heating again from −80 °C to 50 ºC below decomposition temperature at 10 °C/min. The data were processed using TA Instruments TRIOS for Windows software. All reported T_g were taken from the second heating cycle.

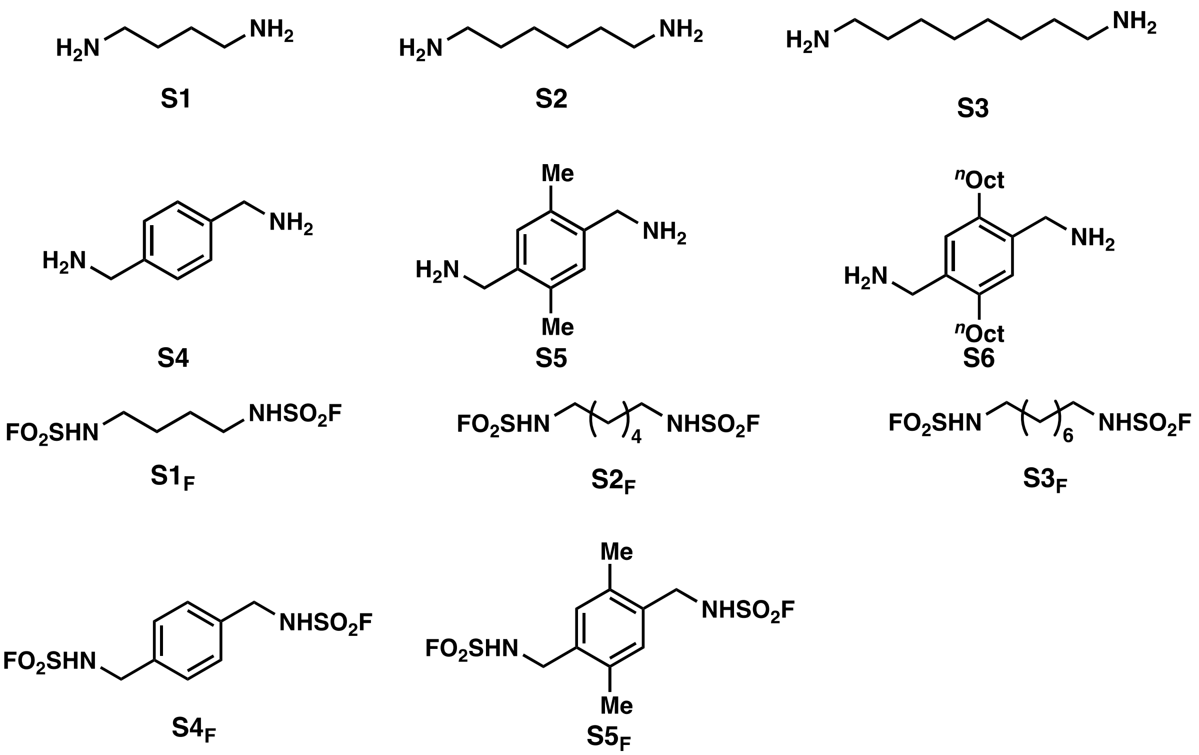

Figure S3. *Bis(amine) and bis(sulfamoyl fluoride) monomers used in this study.*

S.III.A Synthesis of bis(amine) S5 and S6:

S7 and **S8** were synthesized following a protocol from the literature (*J. Am. Chem. Soc.* **2020**, *142*, 11983–11987).

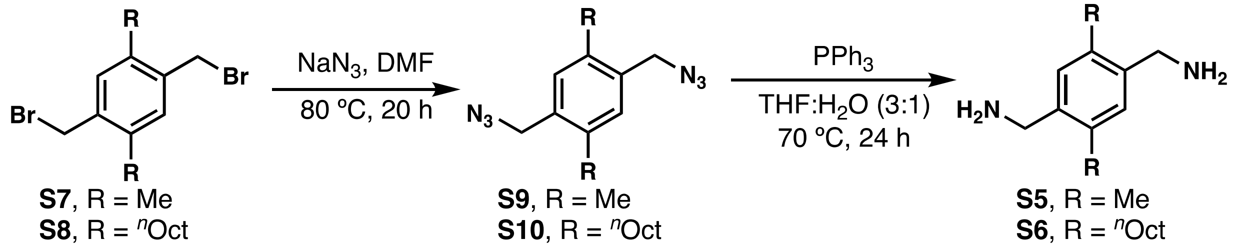

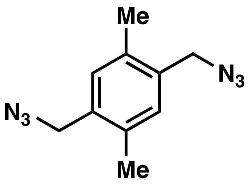

S9

1,4-Bis(azidomethyl)-2,5-dimethylbenzene S9. A mixture of dibromo xylene **S7** (2.92 g, 1.0 equiv, 10 mmol) and sodium azide (1.63 g, 2.5 equiv, 25 mmol) in DMF (25 mL) was heated to 80 ºC and stirred for 20 h. The crude mixture was cooled to room temperature, diluted with water (100 mL) and extracted with EtO_2 (3 × 200 mL). The organic layers were combined, washed with water (3 × 100 mL), dried over $MgSO_4$, and concentrated *in vacuo.* Column chromatography (SiO_2, 100% Hexane) afforded **S9** as a white solid (2.13 g, 98%).

^{1}H NMR ($CDCl_3$, 500 MHz) δ: 7.12 (s, 2 H), 4.32 (s, 4 H), 2.34 (s, 6 H) ppm.
^{13}C NMR ($CDCl_3$, 126 MHz) δ: 134.6, 133.8, 131.7, 52.8, 18.6 ppm.
HRMS-ESI: calc'd. for $C_{10}H_{13}N_4$ $[M–N_2+H]^+$ 189.1135, found 189.1133.
m.p.: 64–65 ºC.

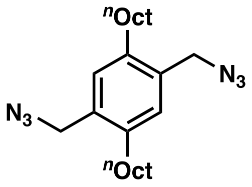

S10

1,4-Bis(azidomethyl)-2,5-dioctylbenzene S10. A mixture of dibromo xylene **S8** (195 mg, 1.0 equiv, 0.4 mmol) and sodium azide (65 mg, 2.5 equiv, 1.0 mmol) in DMF (1 mL) was heated to 80 ºC and stirred for 20 h. The crude mixture was cooled to room temperature, diluted with water (10 mL) and extracted with EtO_2 (3 × 10 mL). The organic layers were combined, washed with water (3 × 10 mL), dried over $MgSO_4$, and concentrated *in vacuo.* Column chromatography (SiO_2, 100% Hexane) afforded **S10** as a white solid (153 mg, 87%).

^{1}H NMR ($CDCl_3$, 500 MHz) δ: 7.12 (s, 2 H), 4.34 (s, 4 H), 2.61 (t, 4 H, J = 8.2 Hz), 1.56 (m, 4 H), 1.39–1.24 (m, 20 H), 0.88 (t, 6 H, J = 6.8 Hz) ppm.

^{13}C NMR ($CDCl_3$, 126 MHz) δ: 139.4, 133.2, 131.1, 52.4, 32.3, 32.0, 31.3, 29.8, 29.6, 29.4, 22.8, 14.2 ppm.
HRMS-ESI: calc'd. for $C_{24}H_{41}N_4$ $[M–N_2+H]^+$ 385.3326, found 385.3325.
m.p.: 34–35 ºC.

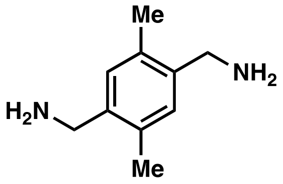
S5

1,4-Bis(aminomethyl)-2,5-dimethylbenzene S5 was prepared using a modified procedure from the literature (*Org. Biomol. Chem.* **2019**, *17*, 3285–3291). Triphenylphosphine (1.05 g, 4.0 equiv, 4.0 mmol) was added in portions into a solution of xylene bis(azide) **S9** (214 mg, 1.0 equiv, 1.0 mmol) in a mixture of THF:H_2O (3:1) at room temperature. The reaction mixture was then stirred at 70 ºC for 24 h. The solution was cooled to room temperature, diluted with EtOAc (10 mL), and washed with water (3 × 10 mL). The organic phase was dried over $MgSO_4$ and concentrated in vacuo. Column chromatography (SiO_2, 10% MeOH in DCM with 1% TEA) afforded **S5** as a pale white solid (132 mg, 80%).
^1H NMR ($CDCl_3$, 500 MHz) δ: 7.10 (s, 2 H), 3.82 (s, 4 H), 2.31 (s, 6 H), 1.49 (br, 4 H) ppm.
^{13}C NMR ($CDCl_3$, 126 MHz) δ: 139.7, 133.2, 129.6, 44.0, 18.5 ppm.
HRMS-ESI: calc'd. for $C_{10}H_{17}N_2$ $[M+H]^+$ 165.1386, found 165.1386.
m.p.: decomposed before melting.

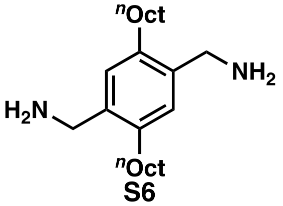
S6

1,4-Bis(aminomethyl)-2,5-dioctylbenzene S6 was prepared using a modified procedure from the literature (*Org. Biomol. Chem.* **2019**, *17*, 3285–3291). Triphenylphosphine (525 mg, 4.0 equiv, 2.0 mmol) was added in portions into a solution of xylene diazide **S10** (220 mg, 0.5 mmol) in a mixture of THF:H_2O (3:1) at the room temperature. The reaction mixture was then stirred at 70 ºC for 24 h. The solution was cooled to room temperature, diluted with EtOAc (10 mL) and wash with water (3 × 10 mL). The organic phase was collected, dried over $MgSO_4$, and concentrated in vacuo. The crude was purified by column chromatography (SiO_2, 10% MeOH in DCM with 1% TEA) afforded **S6** as a pale white solid (159 mg, 87%).

^1H NMR ($CDCl_3$, 500 MHz) δ: 7.11 (s, 2 H), 3.84 (s, 4 H), 2.60 (t, 4 H, J = 8.2 Hz), 1.75 (br, 4 H), 1.57 (m, 4 H), 1.40–1.23 (m, 20 H), 0.88 (m, 6 H) ppm.

^{13}C NMR ($CDCl_3$, 126 MHz) δ: 139.3, 138.2, 128.9, 43.4, 32.4, 32.0, 31.7, 30.0, 29.7, 29.4, 22.8, 14.2 ppm.
HRMS-ESI: calc'd. for $C_{24}H_{45}N_2$ $[M+H]^+$ 361.3577, found 361.3567.
m.p.: 65–66 ºC.

S.III.B. General procedure for the synthesis of bis(sulfamoyl fluoride) monomers (using compound $S2_F$ as an example):

Fluorosulfamoylation reagent **S11** and bis(sulfamoyl fluoride) were prepared using procedures reported in our previous study (*Chem. Sci.* **2020**, *11*, 7807–7812). Previously unknown bis(sulfamoyl fluoride) monomers were prepared following the same general procedure described below with the appropriate bis(amine) starting materials.

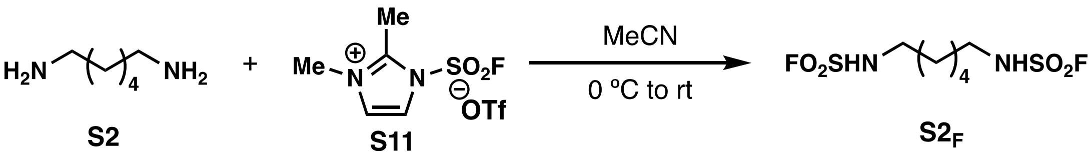

S11 (1.31 g, 4.0 mmol, 2.0 equiv) was added to a solution of bis(amine) **S2** (232 mg, 2.0 mmol, 1.0 equiv) in MeCN (8 mL) at 0 °C (for some substrates DCM was used instead of MeCN to improve the solubility of the reactants). The mixture was warmed to room temperature and stirred until the starting material was fully consumed (2–4 h, monitored by TLC). The mixture was then diluted with ethyl acetate (15 mL) and washed with aq. HCl (C = 0.1 M, 2 × 15 mL) and brine (15 mL). The organic phase was subsequently dried over $MgSO_4$ and concentrated *in vacuo.* Column chromatography was used for compounds presenting minor impurities as specified below.

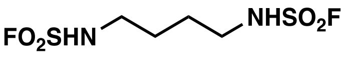

$S1_F$

Bis(sulfamoyl fluoride) $S1_F$ was obtained using bis(amine) **S1** (176 mg, 0.2 mL, 2.0 mmol, 1.0 equiv) and MeCN as the solvent. Column chromatography (SiO_2, 20:90 EtOAc:hexanes) provided **$S1_F$** as a yellowish oil (275 mg, 55%).

^{1}H NMR ($CDCl_3$, 500 MHz) δ: 5.01 (br, 2 H, NH), 3.36 (m, 4 H), 1.74 (m, 4 H) ppm.
^{13}C NMR ($CDCl_3$, 126 MHz) δ: 44.1, 26.4 ppm.
^{19}F NMR ($CDCl_3$, 470 MHz) δ: 51.2 ppm.
HRMS-ESI: calc'd. for $C_4H_9N_2S_2O_4F_2$ $[M–H]^–$ 250.9966, found 250.9975.

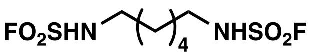

$S2_F$

Bis(sulfamoyl fluoride) $S2_F$ was obtained using bis(amine) **S2** (345 mg, 3.0 mmol) and MeCN as the solvent. Column chromatography (SiO_2, 10:90 to 20:80 EtOAc:hexanes) provided **$S2_F$** as a yellow oil product (576 mg, 68%). Note: **S2d** solidified when storage at 4 ºC overnight.

^{1}H NMR ($CDCl_3$, 400 MHz) δ: 4.97 (br, 2 H, NH), 3.31 (m, 4 H), 1.65 (m, 4 H), 1.41 (m, 4 H) ppm.

^{13}C NMR (d_6-DMSO, 126 MHz) δ: 44.5, 29.3, 25.7 ppm.

^{19}F NMR (d_6-DMSO, 470 MHz) δ: 50.8 ppm.

HRMS-ESI: calc'd. for $C_6H_{13}N_2S_2O_4F_2$ $[M–H]^-$ 279.0279, found 279.0290.

m.p.: 44–45 ºC.

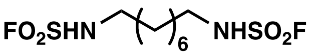

$S3_F$

Bis(sulfamoyl fluoride) $S3_F$ was obtained using bis(amine) **S3** (289 mg, 2.0 mmol) and DCM as the solvent. Column chromatography (SiO_2, 5:95 to 10:90 EtOAc:hexanes) provided **$S3_F$** as a white solid (300 mg, 49%).

^{1}H NMR ($CDCl_3$, 400 MHz) δ: 4.87 (br, 2 H, NH), 3.30 (m, 4 H), 1.63(m, 4 H), 1.35 (m, 8 H) ppm.

^{13}C NMR ($CDCl_3$, 126 MHz) δ: 44.8, 29.4, 28.9, 26.2 ppm.

^{19}F NMR ($CDCl_3$, 470 MHz) δ: 51.0 ppm.

HRMS-ESI: calc'd. for $C_8H_{17}N_2S_2O_4F_2$ $[M–H]^-$ 307.0592, found 307.0603.

m.p.: 47–48 ºC.

Bis(sulfamoyl fluoride) $S4_F$ were synthesized and characterized in our previous study: *Chem. Sci.* **2020**, *11*, 7807–7812.

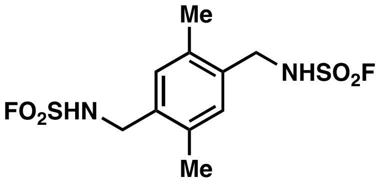

$S5_F$

Bis(sulfamoyl fluoride) $S5_F$ was obtained as a pale-yellow solid using bis(amine) **S5** (33 mg, 0.2 mmol) and DCM as the solvent. Column chromatography (SiO_2, 20:80 EtOAc:hexanes) provided **$S5_F$** as a white solid (31 mg, 46%).

^{1}H NMR (d_3-MeCN, 500 MHz) δ: 7.17 (s, 2 H), 6.92 (br, 2 H), 4.38 (d, 4 H, J = 3.6 Hz), 2.31 (s, 6 H) ppm.

^{13}C NMR (d_3-MeCN, 126 MHz) δ: 135.4, 134.7, 132.2, 46.3, 18.5 ppm.

^{19}F NMR (d_3-MeCN, 470 MHz) δ: 48.7 ppm.

HRMS-ESI: calc'd. for $C_{10}H_{13}F_2N_2O_4S_2$ $[M–H]^-$ 327.0279, found 327.0290.

m.p.: decomposed before melting.

S.III.C. Synthetic procedures of preparing *N*,*N*'-disubstituted sulfamides (Sulf 1–3)

Compound **Sulf-1$_{ex}$** and **Sulf-2** were prepared using a modified procedure from the literature (*Polym. Adv. Technol.* **2011**, *22*, 1529–1538). Recrystallization of **Sulf-1$_{ex}$** was performed via vapor diffusion of hexanes through a solution of **Sulf-1$_{ex}$** in EtOAc and afforded **sulf-1$_{rec}$**. Annealing was performed by heating a vial containing **Sulf-1$_{ex}$** at 130 ºC using an oil bath followed by then slow cooling to 25 ºC over 2 h and afforded **Sulf-1$_{an}$**.

Compound **Sulf-3** was prepared through a two-step synthesis based on SuFEx click chemistry.

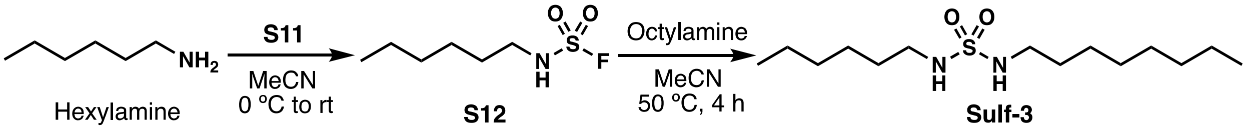

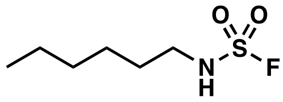

S12

A mixture of hexylamine (0.26 mL, 1.0 equiv, 2.0 mmol) and MeCN (8 mL) was cooled to 0 °C using an ice bath. At 0 ºC, reagent **S11** (657 mg, 1 equiv, 2.0 mmol) was quickly transferred to the mixture. The reaction mixture was then warmed to room temperature and stirred for 2 h until the starting material was fully reacted as monitored by TLC. The crude mixture was diluted with EtOAc (15 mL) and washed with aq. HCl (C = 0.1 M, 2 × 15 mL) and brine (15 mL). The organic layer was dried over $MgSO_4$ and concentrated *in vacuo*. Column chromatography (SiO_2, 10:90 to 15:85 EtOAc:Hexanes) yielded **S2** as a colorless oil (340 mg, 93%).

^{1}H NMR (500 MHz, $CDCl_3$) δ: 4.88 (br, 1 H, NN), 3.30 (tdd, 2 H, *J* = 7.5, 5.9, 2.0 Hz), 1.62 (q, 2 H, *J* = 7.4 Hz), 1.40–1.27 (m, 6 H), 0.90 (t, 3 H, *J* = 6.9 Hz) ppm.

^{13}C NMR ($CDCl_3$, 126 MHz) δ: 44.9, 31.3, 29.4, 26.1, 22.6, 14.0 ppm.

^{19}F NMR ($CDCl_3$, 470 MHz) δ: 50.6 ppm.

HRMS-ESI: calc'd. for $C_6H_{13}NSO_2F$ $[M–H]^-$ 182.0646, found 182.0647.

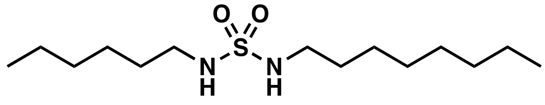

Sulf-3

Octylamine (0.11 mL, 1.0 equiv) and DBU (0.1 mL, 1.0 equiv) were added to a solution of sulfamoyl fluoride **S12** (124 mg, 1.0 equiv) in acetonitrile (0.7 mL). The reaction mixture was heated to 50 ºC and stirred until full consumption of sulfamoyl

fluoride **S12** (about 4 h). The reaction was carefully quenched after cooling to room temperature with aq. HCl (C = 0.1 M, 10 mL). The mixture was extracted with ethyl acetate (3 × 5 mL). The organic fractions were combined, dried over $MgSO_4$, and concentrated *in vacuo* to yield **Sulf-3** as a white crystalline solid (128 mg, 91%).

^{1}H NMR ($CDCl_3$, 500 MHz,) δ: 4.36 (br, 2 H, NH), 3.02 (t, 4 H, *J* = 7.3 Hz), 1.54 (p, 4 H, *J* = 7.3 Hz), 1.37–1.26 (m, 16 H), 0.88 (m ,6 H) ppm.

^{13}C NMR ($CDCl_3$, 126 MHz) δ: 43.4, 43.4, 31.9, 31.5, 29.7, 29.7, 29.3, 29.3, 26.9, 26.5, 22.8, 22.7, 14.2, 14.1 ppm.

HRMS-ESI: calc'd. for $C_{14}H_{32}SO_2N_2$ $[M+H]^+$ 293.2257, found 293.2254.

m.p.: 94–96 ºC.

S.III.D General procedure for the synthesis of polysulfamides (using poly-II-b as an example)

Poly-I-a–c were synthesized and characterized in our previous study: *Chem. Sci.* **2020**, *11*, 7807–7812. All other polysulfamides were prepared following the general procedure below.

Polysulfamides were synthesized from commercial bis(amine)s and bis(sulfamoyl) fluorides following a previously reported procedure (*Chem. Sci.* **2020**, *11*, 7807–7812) with slight modifications.

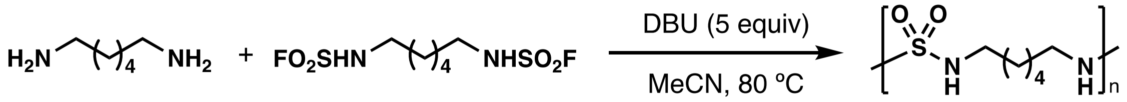

S2 **S2$_F$** **poly-II-b**

To a solution of bis(sulfamoyl fluoride) **S2$_F$** (112 mg, 0.4 mmol, 1.0 equiv) in MeCN (0.8 mL, C = 0.5 M), DBU (0.3 mL, 2.0 mmol, 5.0 equiv) and bis(amine) **S2** (47 mg, 0.4 mmol, 1.0 equiv) were added. The mixture was stirred at 80 ºC for 90 min. The resulting mixture was dissolved in DMF (1 mL), then precipitated in a centrifuge tube through the addition of Et_2O, until a volume of 50 mL was reached. After centrifuging and removing the supernatant liquid, the resulting dark orange solid was re-dissolved in DMF (1 mL) and re-precipitated through the addition of saturated NH_4Cl solution until a volume of 50 mL was reached. The solid was then washed with saturated $NaHCO_3$ (50 mL) and distilled water (2 × 50 mL). The polymer was dried *in vacuo* (< 1 mmHg) at 100 ºC for 18 h to obtain the final polymer as a white solid. Additional washes with MeCN were used in a few instances to remove minor impurities (see below).

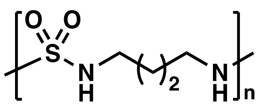

poly-II-a

Polysulfamide poly-II-a was obtained as a white solid starting from **S1** (41µL, 0.4 mmol) and **S1$_F$** (111 mg, 0.4 mmol). In order to remove some remaining minor impurities, the solid was washed with MeCN (3 × 50 mL) and dried under vacuum.

^{1}H NMR (d_6-DMSO, 500 MHz) δ: 6.71 (t, 2 H, J = 5.8 Hz), 2.78 (m, 4 H), 1.46 (m, 4 H) ppm.
^{13}C NMR (d_6-DMSO, 126 MHz) δ: 42.4, 26.9 ppm.
FTIR $\tilde{\upsilon}$: 3274, 2945, 2872, 1435, 1316, 1143, 1064, 937, 660, 561cm^{-1}.

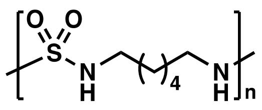

poly-II-b

Polysulfamide poly-II-b was obtained as a white solid starting from compound **S2** (47 mg, 0.4 mmol) and **S2$_F$** (112 mg, 0.4 mmol). In order to remove some remaining minor impurities, the solid was washed with MeCN (3 × 50 mL) and dried under vacuum.

^{1}H NMR (d_6-DMSO, 500 MHz) δ: 6.67 (t, 2 H, J = 5.8 Hz), 2.77 (q, 4 H, J = 6.8 Hz), 1.42 (m, 4 H), 1.27 (m, 4 H) ppm.
^{13}C NMR (d_6-DMSO, 126 MHz) δ: 42.1, 28.9, 26.0 ppm.
FTIR $\tilde{\upsilon}$: 3278, 2940, 2860, 1437, 1305, 1142, 1065, 1028, 924, 671, 544 cm^{-1}.

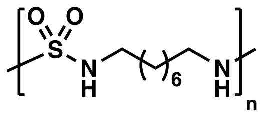

poly-II-c

Polysulfamide poly-II-c was obtained as a white solid starting from compound **S3** (144 mg, 1.0 mmol) and **S3$_F$** (308 mg, 1.0 mmol. In order to remove some remaining minor impurities, the solid was washed with MeCN (3 × 50 mL) and dried under vacuum.

^{1}H NMR (d_6-DMSO, 500 MHz) δ: 6.66 (br, 2 H, NH), 2.77 (m, 4 H), 1.43 (m, 4 H), 1.24 (m, 8 H) ppm.
^{13}C NMR (d_6-DMSO, 126 MHz) δ: 42.1, 28.9, 28.6, 26.3 ppm.
FTIR $\tilde{\upsilon}$: 3281, 2926, 2853, 1439, 1316, 1144, 1066, 1000, 930, 678, 565, 536, 491 cm^{-1}.

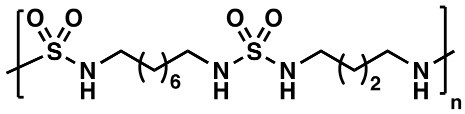

poly-III-a

Polysulfamide poly-III-a was obtained as a white solid starting from compound **S1** (50 µL, 0.5 mmol) and **S3$_F$** (154 mg, 0.5 mmol). In order to remove some remaining minor impurities, the solid was washed with MeCN (3 × 50 mL) and dried under vacuum.

^{1}H NMR (d_6-DMSO, 500 MHz) δ: 6.67 (br, 2 H, NH), 2.79 (m, 4 H), 1.46 (m, 4 H), 1.26 (m, 4 H) ppm.

^{13}C NMR (d_6-DMSO, 126 MHz) δ: 42.7, 42.4, 29.5, 29.1, 26.9, 26.8 ppm.

FTIR $\tilde{\nu}$: 3279, 2936, 2854, 1439, 1316, 1145, 1068, 1000, 934, 672, 565, 531 cm^{-1}.

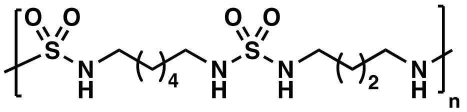

poly-III-b

Polysulfamide poly-III-b was obtained as a white solid starting from compound **S1** (60 µL, 1.0 equiv, 0.6 mmol) and **S2$_F$** (168 mg, 0.6 mmol). In order to remove some remaining minor impurities, the solid was washed with MeCN (3 x 50 mL) and dried under vacuum.

^{1}H NMR (d_6-DMSO, 500 MHz) δ: 6.68 (br, 2 H, NH), 2.77 (m, 4 H), 1.46 (m, 4H), 1.26 (m, 2 H) ppm.

^{13}C NMR (d_6-DMSO, 126 MHz) δ: 42.1, 41.9, 28.9, 26.4, 26.0 ppm.

FTIR $\tilde{\nu}$: 3276, 2942, 2863, 1436, 1306, 1143, 1065, 931, 662, 559, 536 cm^{-1}.

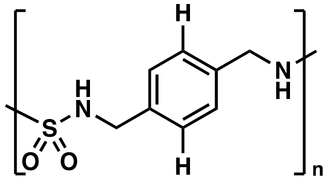

poly-IV-a

Polysulfamide poly-IV-a was obtained as a white solid starting from compound **S4** (68 mg, 0.5 mmol) and **S4$_F$** (150 mg, 0.5 mmol).

The spectroscopic data for this compound were identical to those reported in the literature (*Chem. Sci.* **2020**, *11*, 7807–7812).

^{1}H NMR (d_6-DMSO, 500 MHz) δ: 7.44 (4 H), 7.29 (4 H), 4.01 (4 H) ppm.

^{13}C NMR (d_6-DMSO, 126 MHz) δ: 137.7, 128.1, 46.0 ppm.

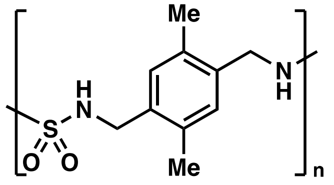

poly-IV-b

Polysulfamide poly-IV-b was obtained as a yellow solid starting from bis(amine) **S5** (38 mg, 0.23 mmol) and compound **S5$_F$** (76 mg, 0.23 mmol).

^{1}H NMR (d_6-DMSO, 500 MHz) δ: 7.26 (2 H), 7.12 (2 H), 3.96 (4 H), 2.24 (6 H) ppm.

^{13}C NMR (d_6-DMSO, 126 MHz) δ: 134.7, 132.8, 130.1, 43.5, 18.2 ppm.

FTIR $\tilde{\nu}$: 3264, 2923, 1653, 1506, 1419, 1303, 1146, 1034, 900, 564 cm^{-1}.

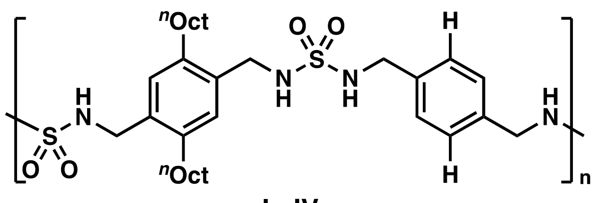

poly-IV-c

Polysulfamide poly-IV-c was obtained as a yellow solid starting from compound **S6** (72 mg, 0.2 mmol) and **S4$_F$** (60 mg, 0.2 mmol).

^{1}H NMR (d_6-DMSO, 500 MHz) δ: 7.43 (2 H), 7.31 (4 H), 7.23 (2 H), 7.14 (2 H), 4.05–3.97 (8 H), 2.56 (4 H), 1.51 94 H), 1.52–1.24 (20 H), 0.84 (6 H) ppm.

^{13}C NMR (d_6-DMSO, 126 MHz) δ: 137.6, 137.2, 134.0, 129.5, 127.5, 45.1, 43.1, 31.4, 31.3, 30.7, 29.2, 28.9, 28.7, 22.0, 13.9 ppm.

FTIR $\tilde{\upsilon}$: 3269, 2962, 2852, 1617, 1424, 1316, 1146, 1053, 898, 560 cm^{-1}.

Section S.IV. Additional Results

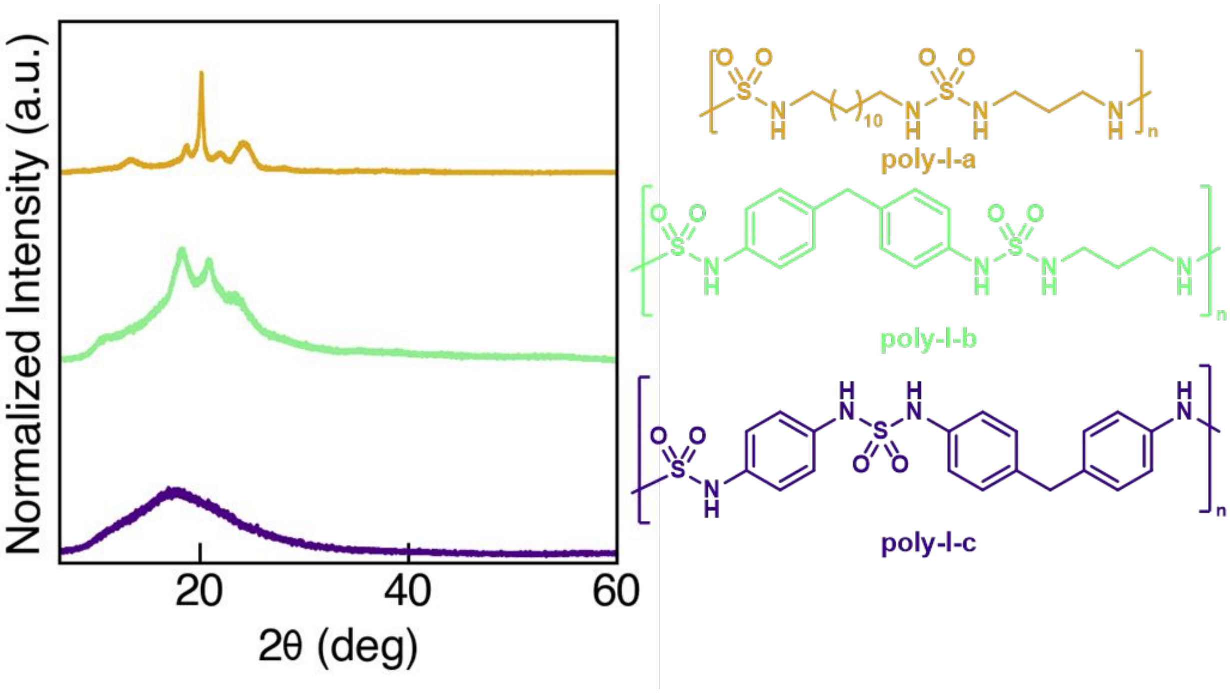

Figure S4. *PXRD patterns of previously synthesized* ***poly-I-a–poly-I-c*** *(experimental Group poly-I). Figures adapted from ref.*[1]

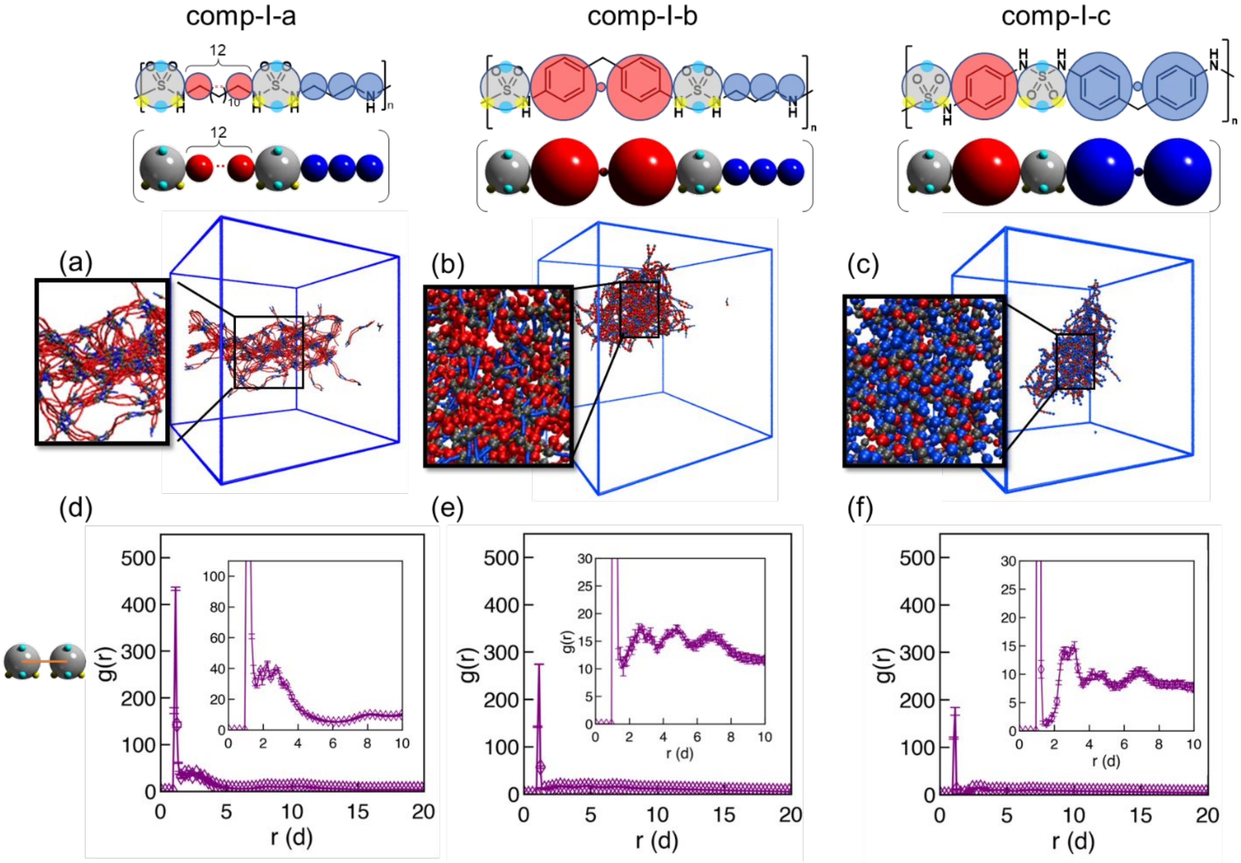

Figure S5. *Additional results for chemistries in group comp-I. (a-c) CG simulation configurations at ϵ_{HB} = 12 kT. Sulfamide beads are represented in grey and repeating units in red and blue. (d-f) sulfamide bead center - sulfamide bead center radial distribution function at ϵ_{HB} = 12 kT. Error bars indicate standard deviation between 9 configurations from three independent simulation trials.*

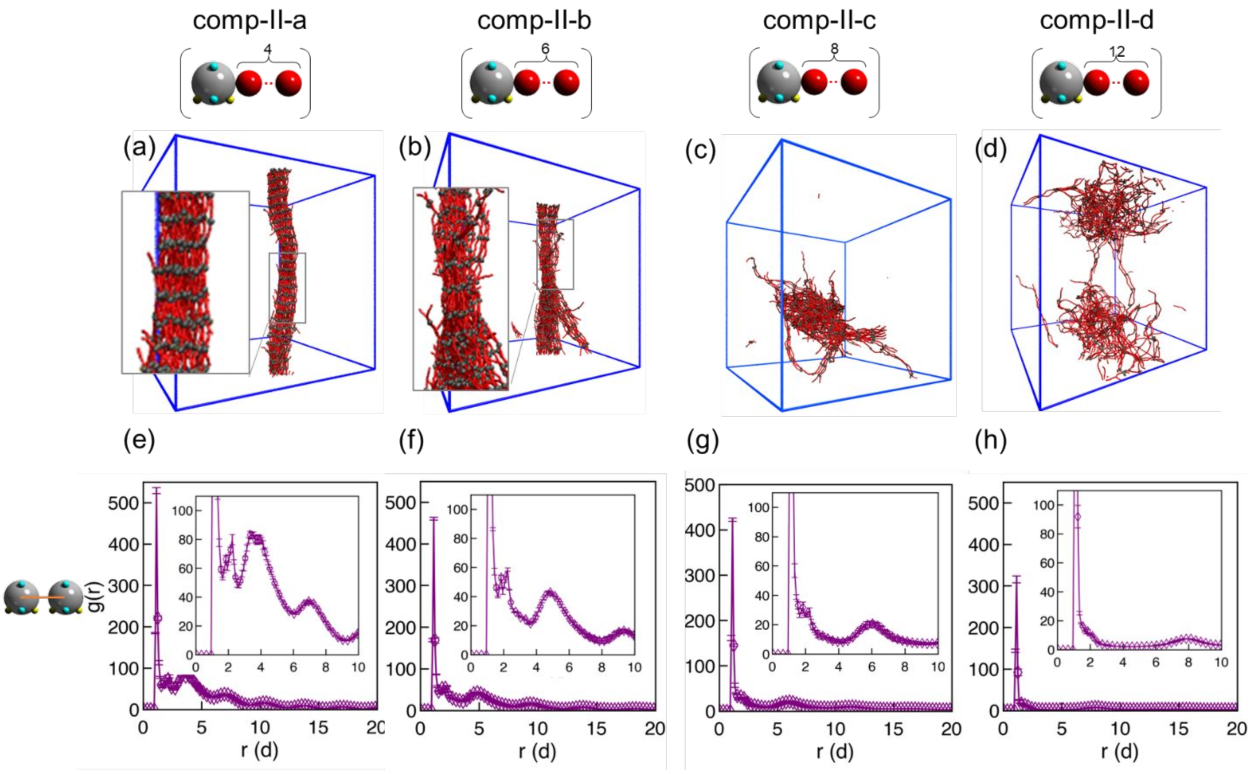

Figure S6. *Additional results for chemistries in group comp-II. (a-d) CG simulation configurations at* ϵ_{HB} *= 12 kT. Sulfamide beads are represented in grey and repeating units in red and blue. (e-h) sulfamide bead-sulfamide bead radial distribution function at* ϵ_{HB} *= 12 kT. Error bars indicate standard deviation between 9 configurations from three independent simulation trials.*

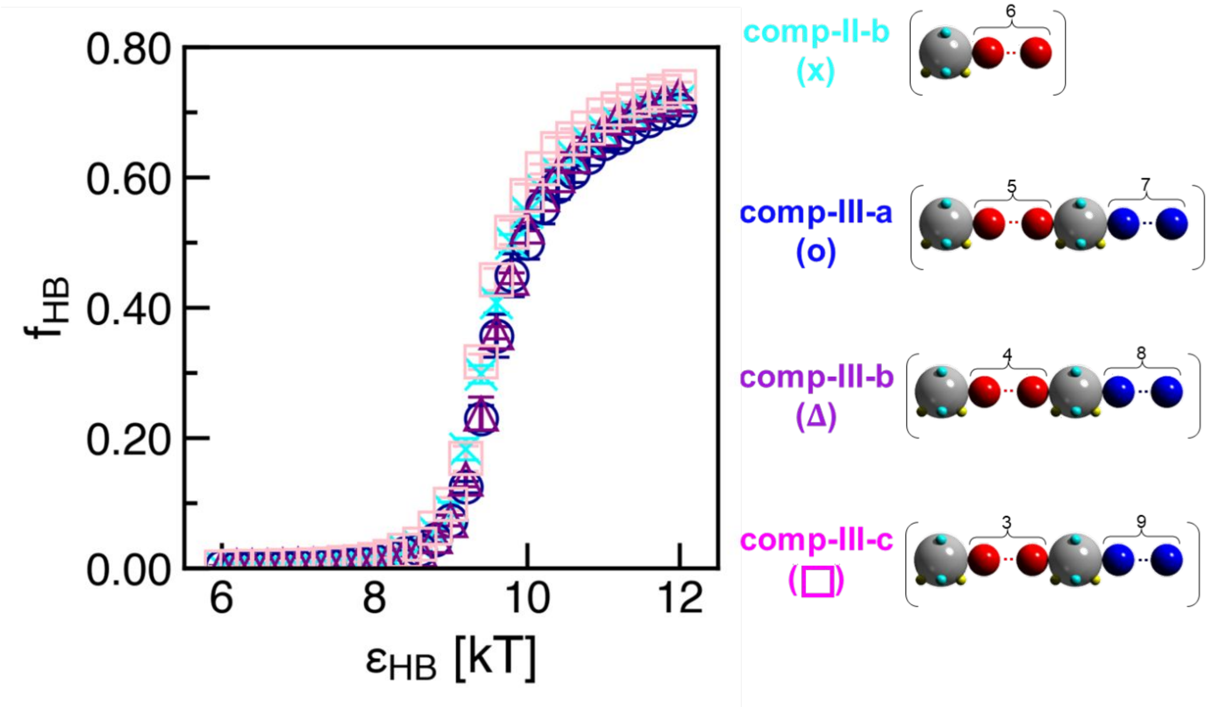

Figure S7. *Hydrogen bonding propensity (f_{HB}) for chemistries in group comp-III at ϵ_{HB} = 6 kT – 12 kT. Error bars indicate standard deviation between 9 configurations from three independent simulation trials.*

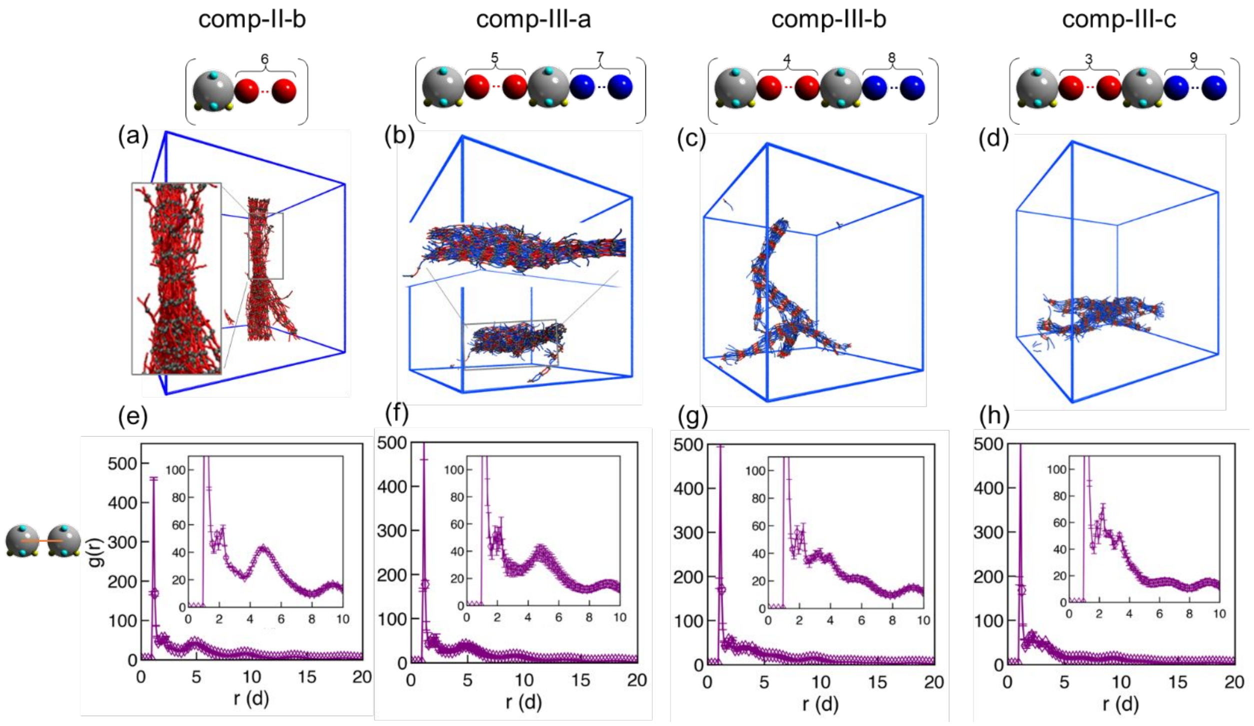

Figure S8. *Additional results for group comp-III. (a-d) CG simulation configurations at ϵ_{HB} = 12 kT. Sulfamide beads are represented in grey and repeating units in red and blue. (e-h) sulfamide bead-sulfamide bead radial distribution function at ϵ_{HB} = 12 kT. Error bars indicate standard deviation between 9 configurations from three independent simulation trials.*

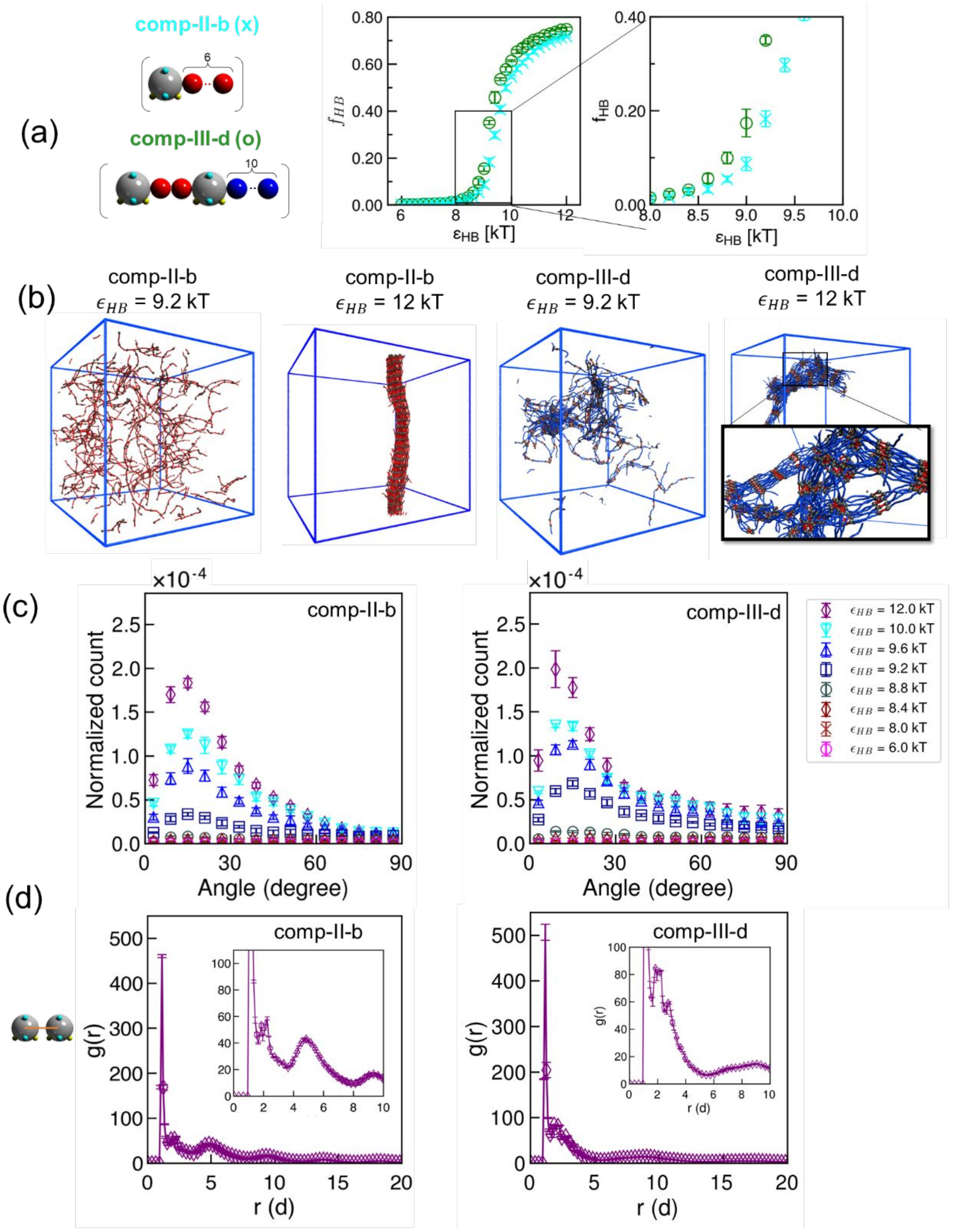

Figure S9. *Additional results comparing Group-II-b against Group-III-d, an additional chemistry not shown in main manuscript. (a) Hydrogen bonding propensity (f_{HB}) at ϵ_{HB} = 6 kT – 12 kT. (b) CG simulation configurations at ϵ_{HB} = 9.2 kT and 12 kT. Sulfamide beads are represented in grey and repeating units in red and blue. (c) Distribution of intersegment angle between H-bonded segments (α_{HB}) at ϵ_{HB} = 6 - 12 kT. (d) sulfamide bead-sulfamide bead radial distribution function at ϵ_{HB} = 12 kT. Error bars indicate standard deviation between 9 configurations from three independent trials.*

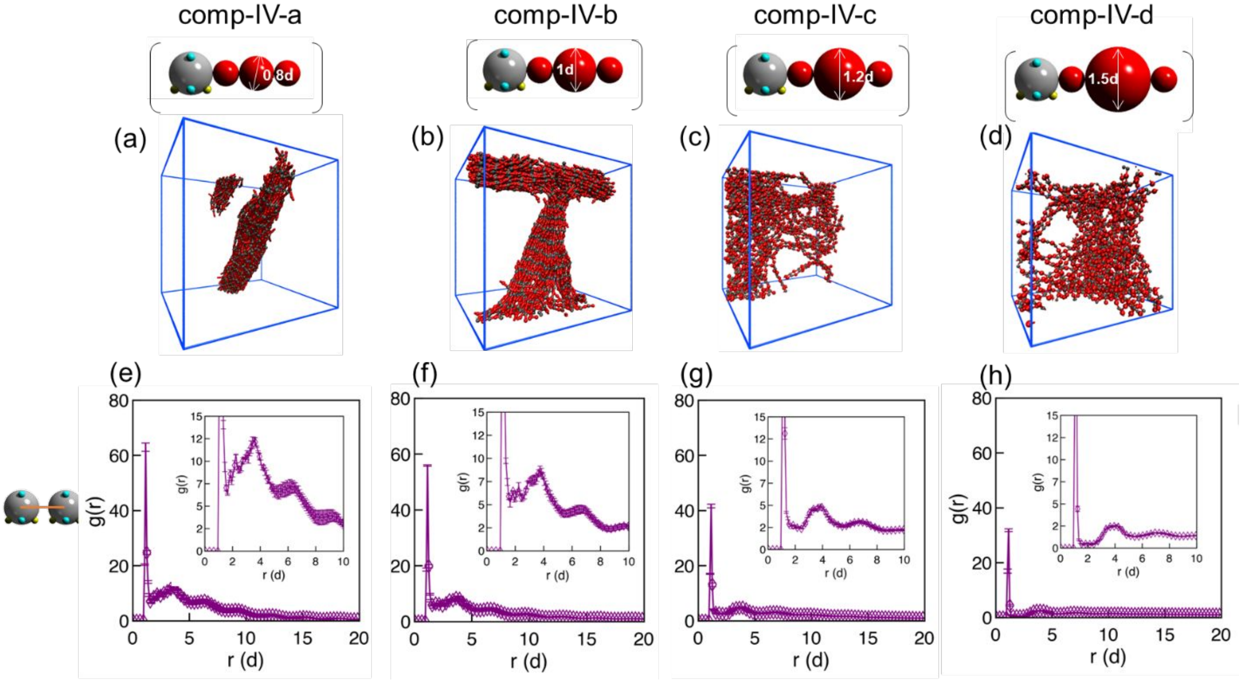

Figure S10. *Additional results for group comp-IV. (a-d) CG simulation configurations at* ϵ_{HB} = *12 kT. Sulfamide beads are represented in grey and repeating units in red and blue. (e-h) sulfamide bead-sulfamide bead radial distribution function at* ϵ_{HB} = *12 kT. Error bars indicate standard deviation between 9 configurations from three independent simulation trials.*

Section S.V. Spectroscopical data and Thermal Behavior of Polysulfamides

FTIR analysis of polysulfamides

Full spectra (4000–500 cm^{-1}) and zoomed-in spectra (1500–500 cm^{-1}) are depicted for each polysulfamide.

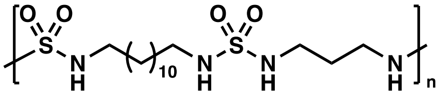

poly-I-a

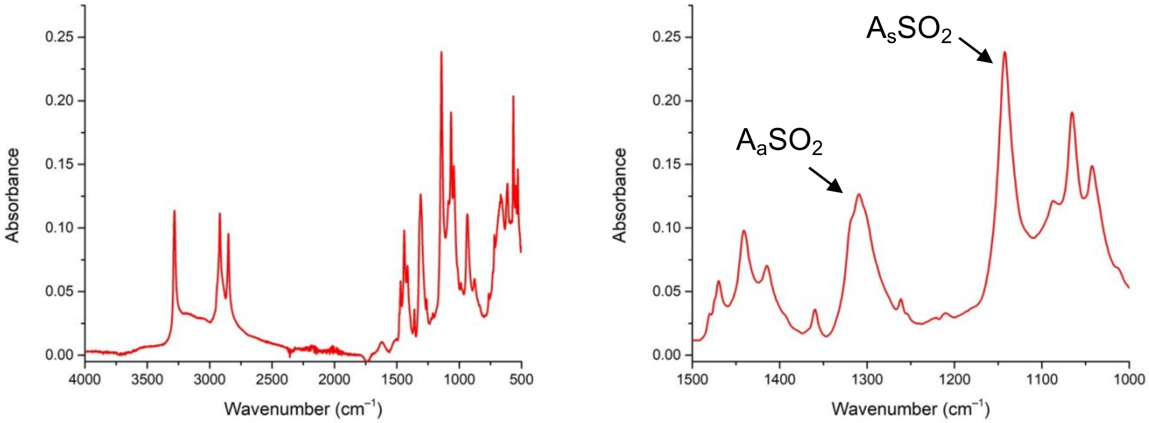

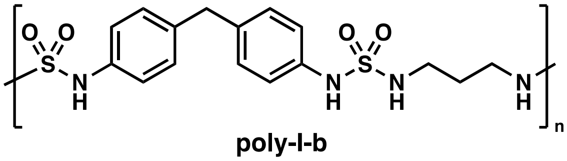

poly-I-b

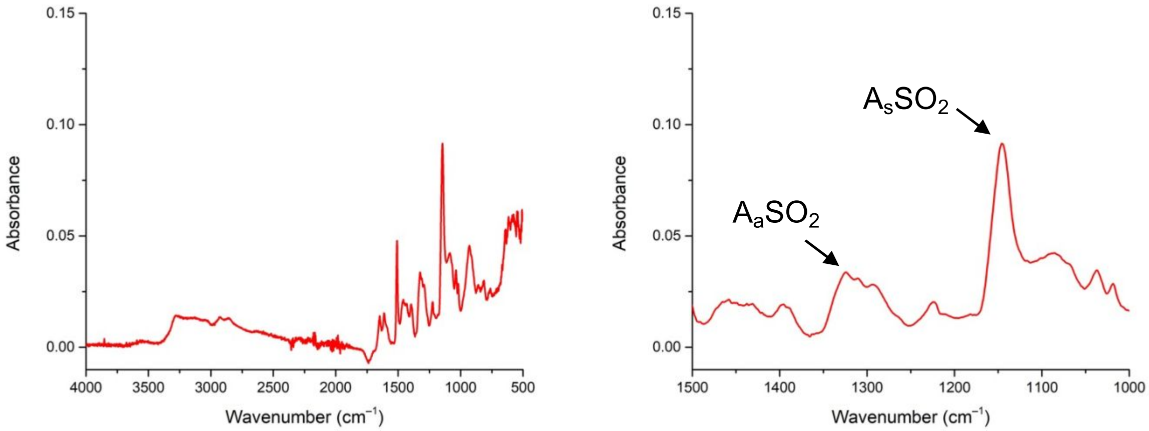

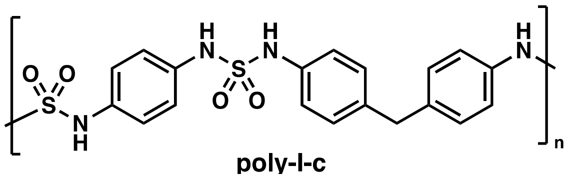

poly-I-c

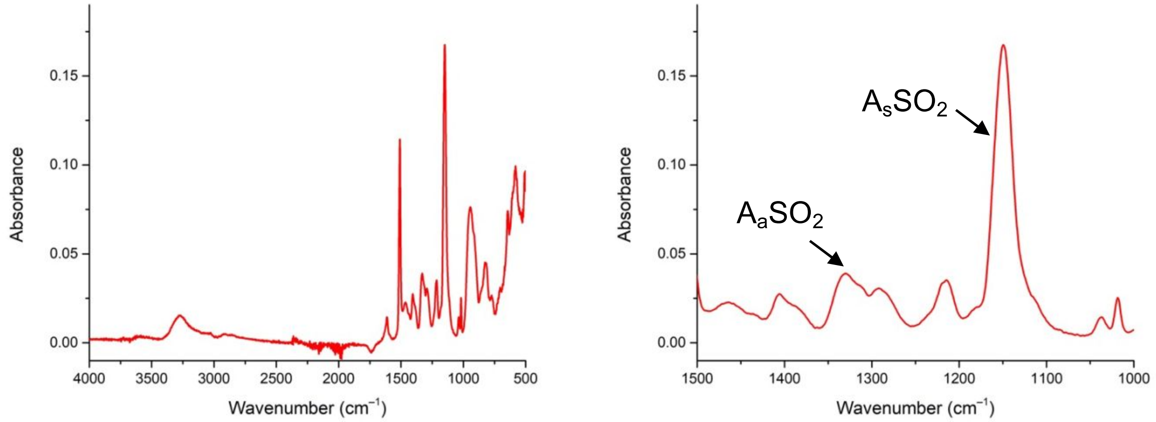

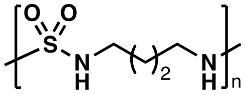

poly-II-a

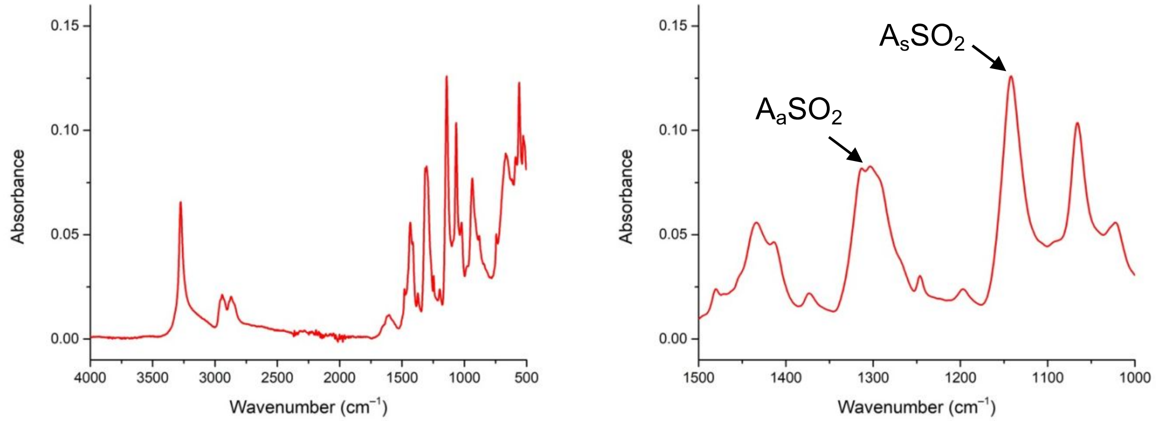

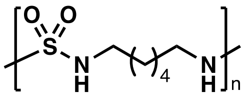

poly-II-b

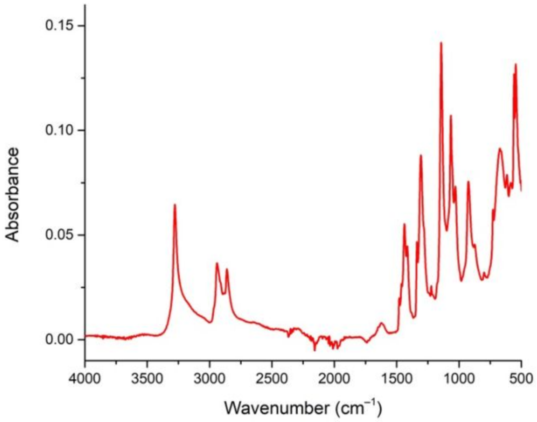

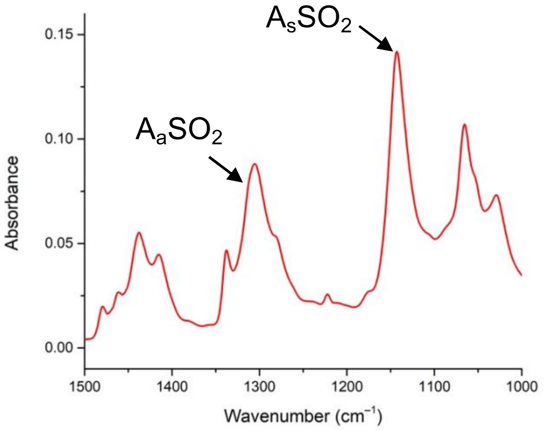

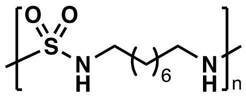

poly-II-c

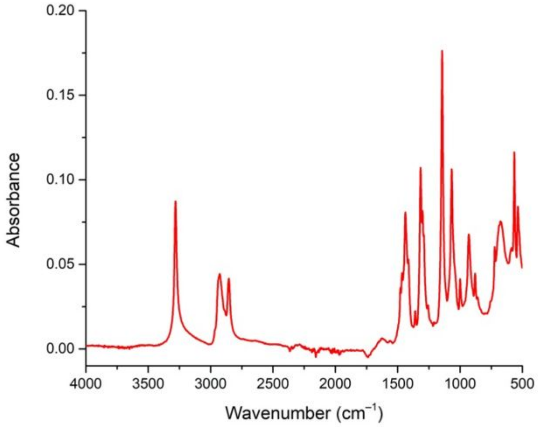

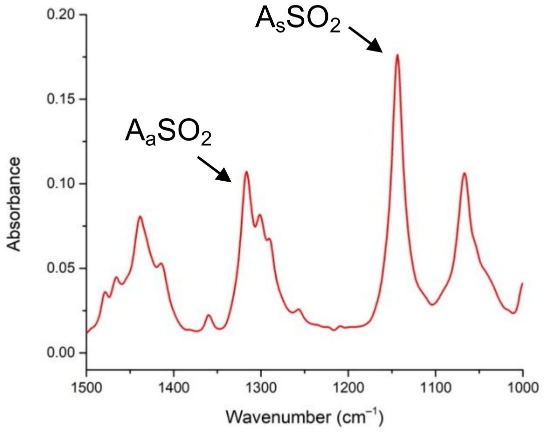

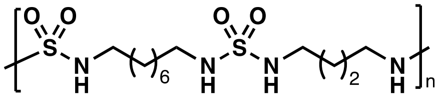

poly-III-a

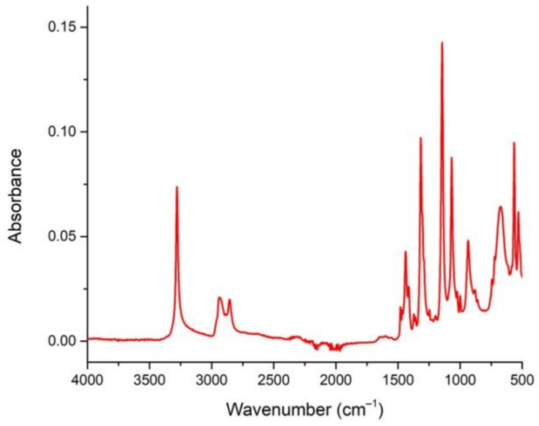

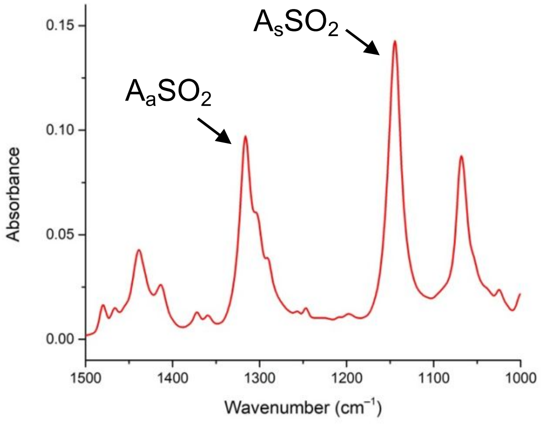

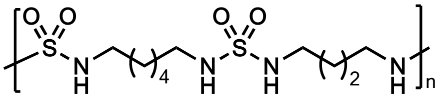

poly-III-b

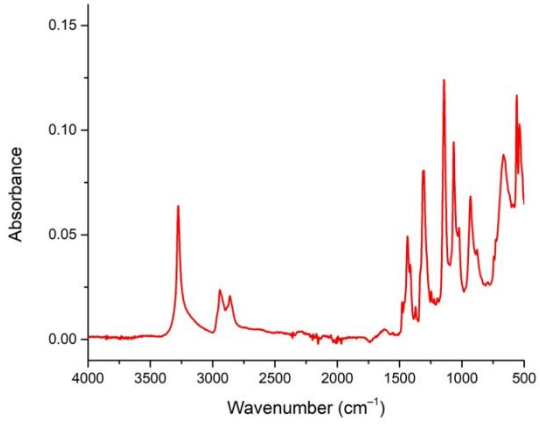

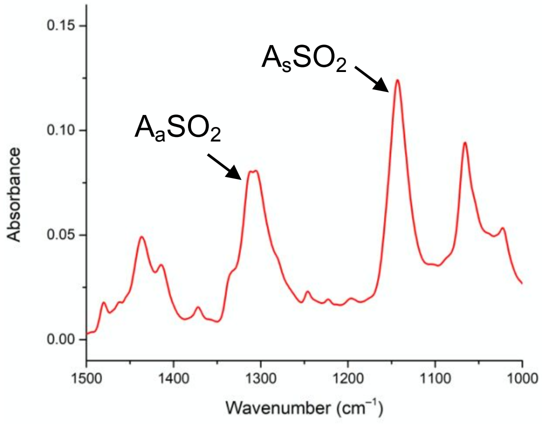

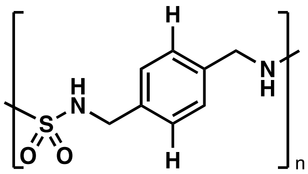

poly-IV-a

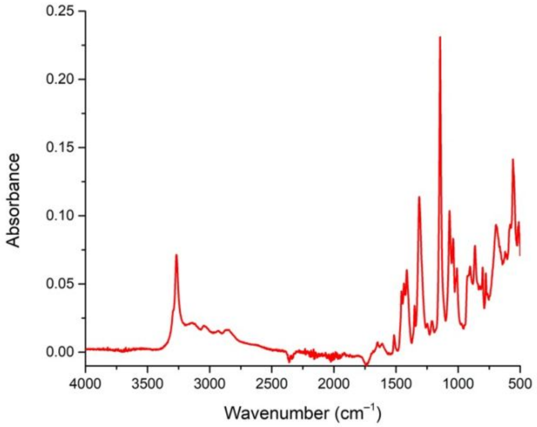

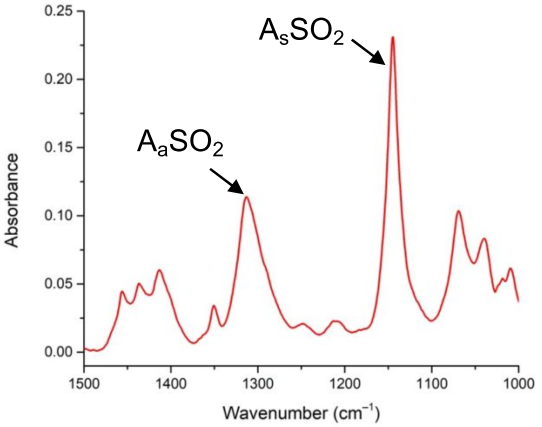

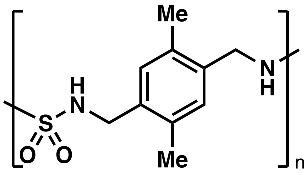

poly-IV-b

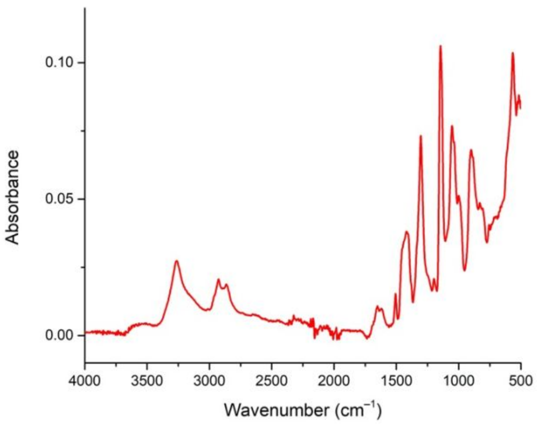

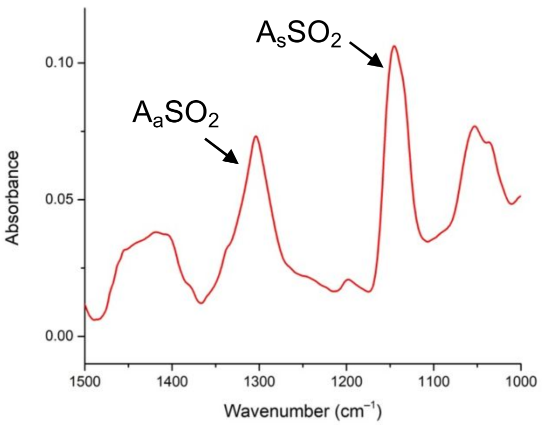

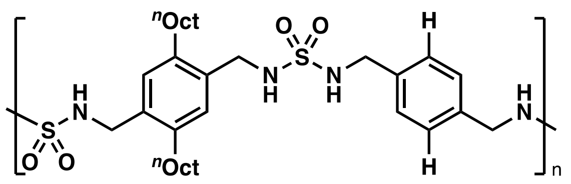
ⁿOct
O
O
S
H
N
N
H
N
H
H
S
O
O
ⁿOct
H
H
N
n

poly-IV-c

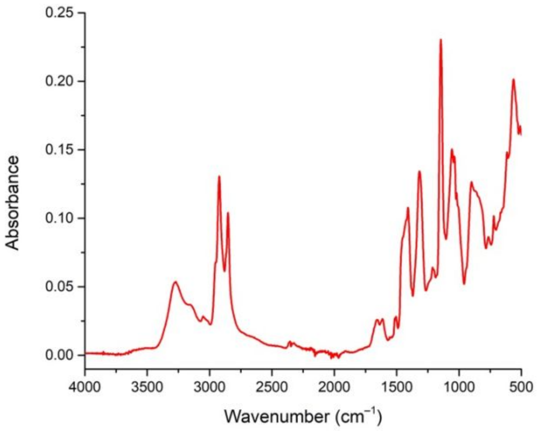
Absorbance
Wavenumber (cm⁻¹)
0.25
0.20
0.15
0.10
0.05
0.00
4000
3500
3000
2500
2000
1500
1000
500

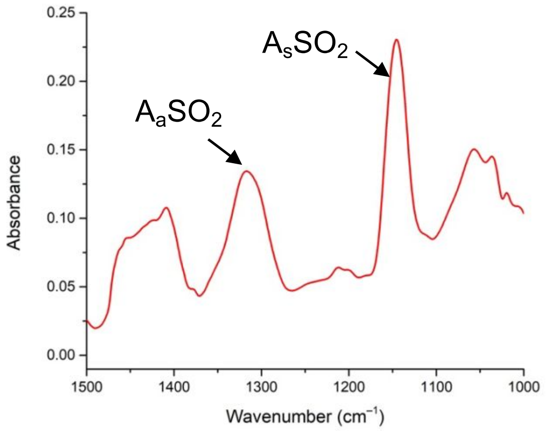
AsSO2
AaSO2
Absorbance
Wavenumber (cm⁻¹)
0.25
0.20
0.15
0.10
0.05
0.00
1500
1400
1300
1200
1100
1000

FTIR analysis of *N,N'*-disubstituted sulfamides (Sulf-2 **and** Sulf-3**):**

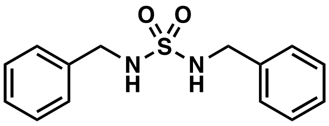

Sulf-2

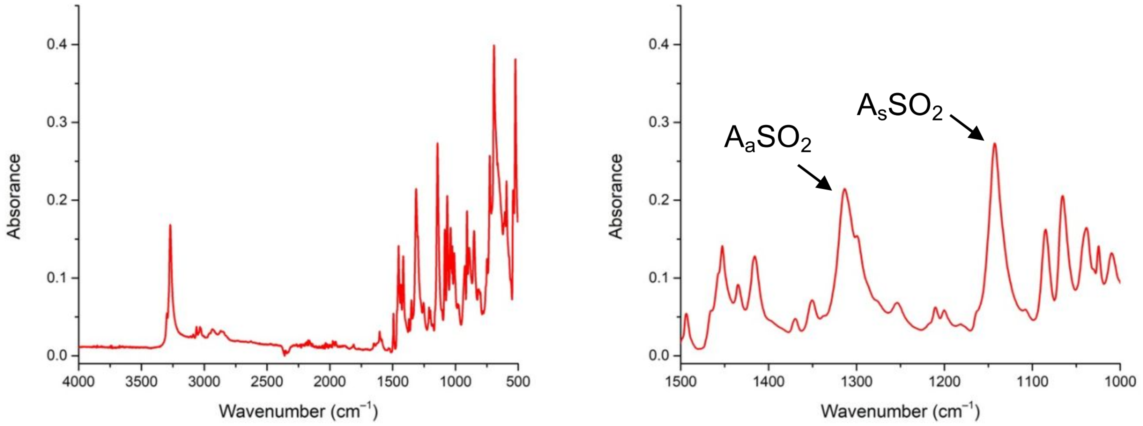

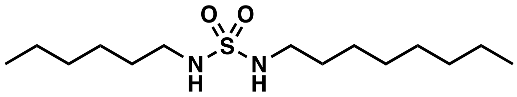

Sulf-3

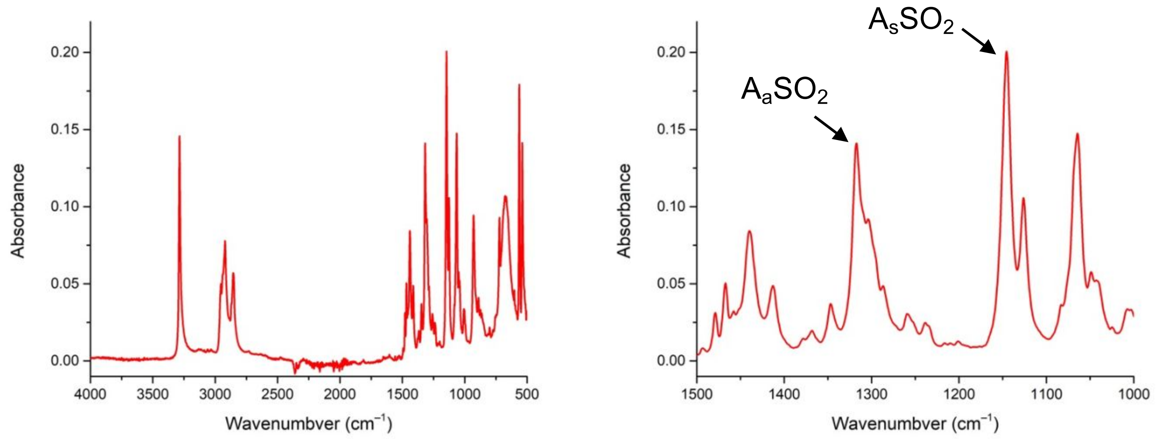

SEC, TGA, and DSC plots data for all polysulfamides:

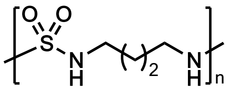

poly-II-a

(a) SEC trace; **(b)** TGA curve; **(c)** DSC curves;

(a)

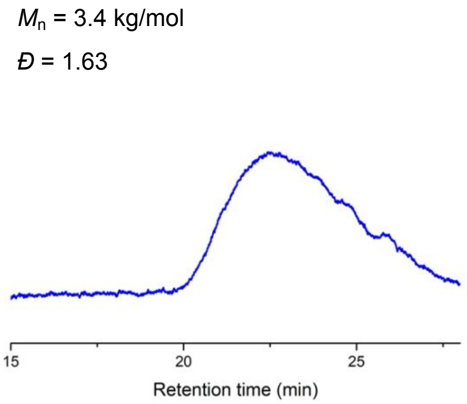

(b)

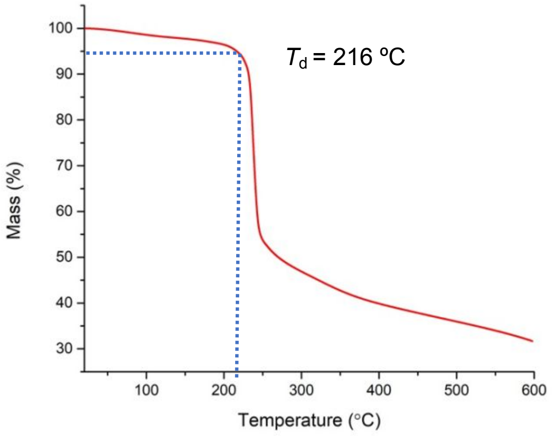

(c)

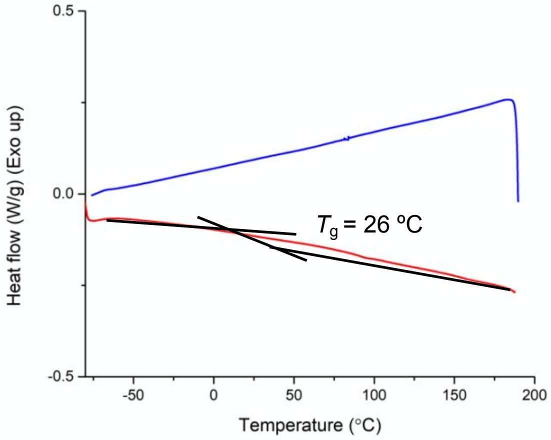

(a)

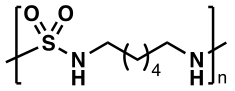

poly-II-b

(a) SEC trace; **(b)** TGA curve; **(c)** DSC curves;

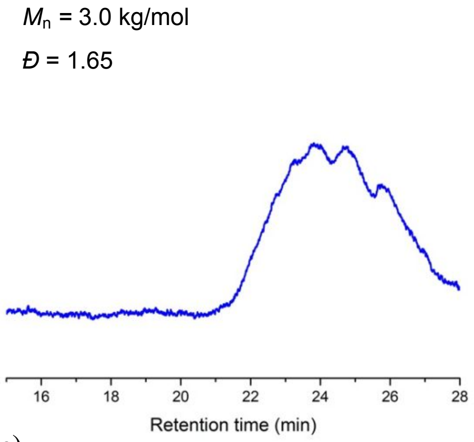

(b)

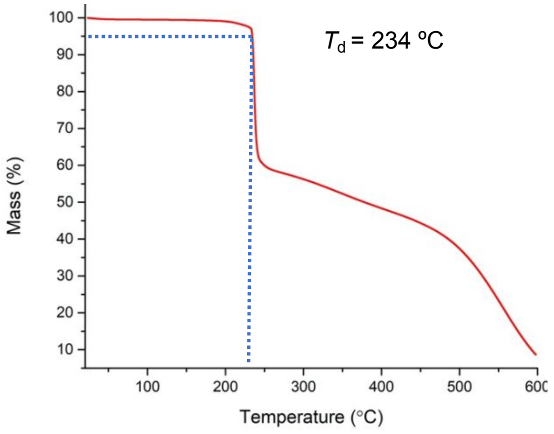

(c)

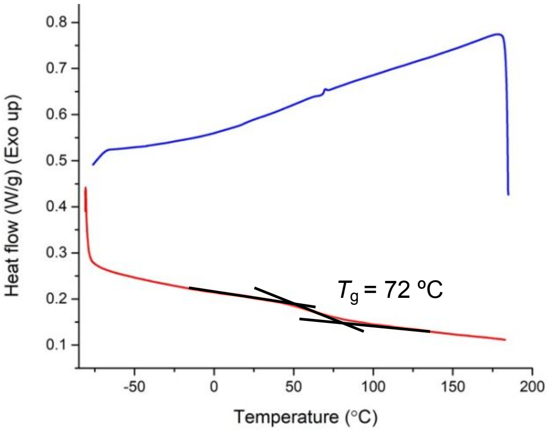

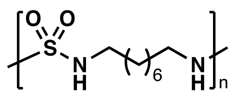

poly-II-c

(a) SEC trace; **(b)** TGA curve; **(c)** DSC curves;

(a)

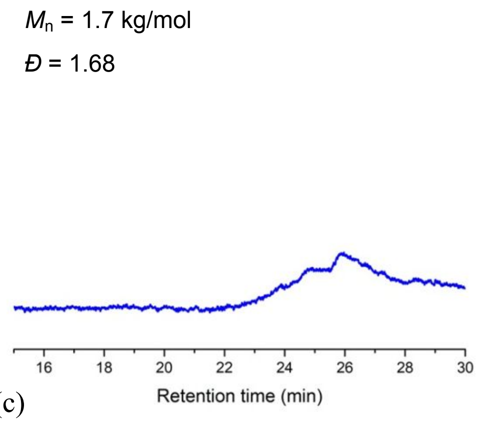

(b)

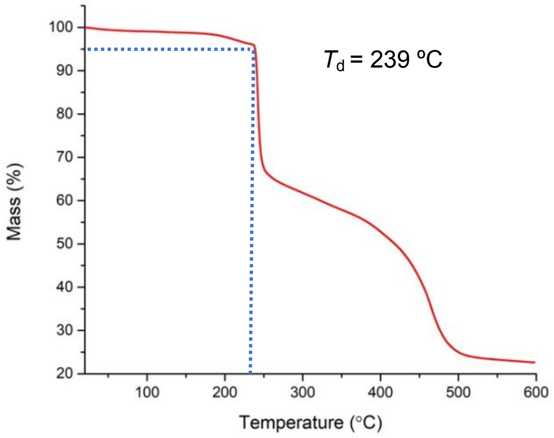

(c)

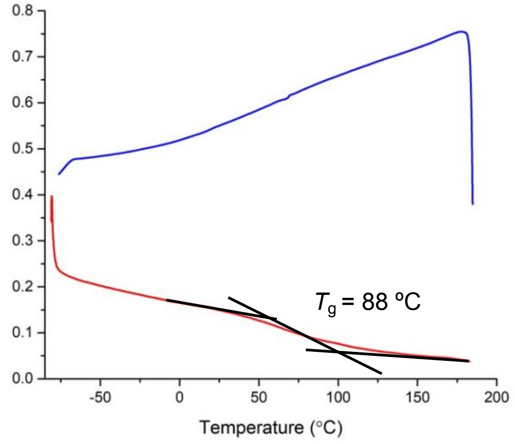

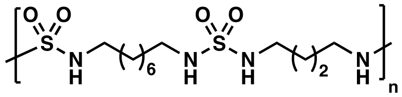

poly-III-a

(a) SEC trace; **(b)** TGA curve; **(c)** DSC curves;

(a)

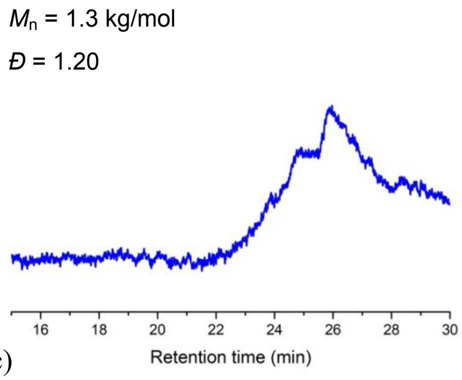

(b)

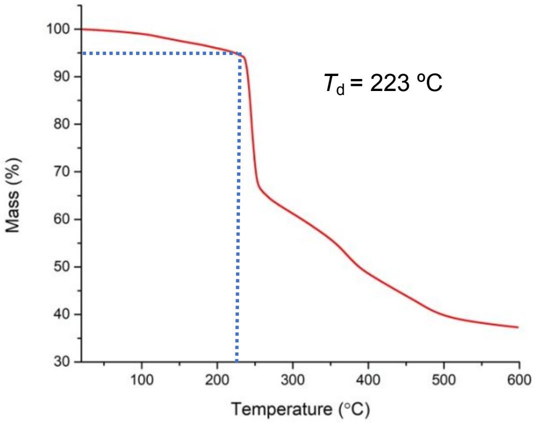

(c)

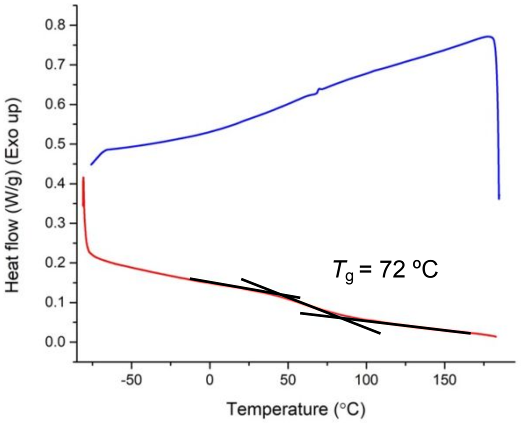

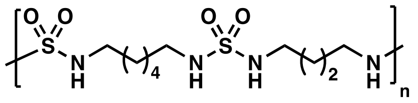

poly-III-b

(a) SEC trace; **(b)** TGA curve; **(c)** DSC curves;

(a)

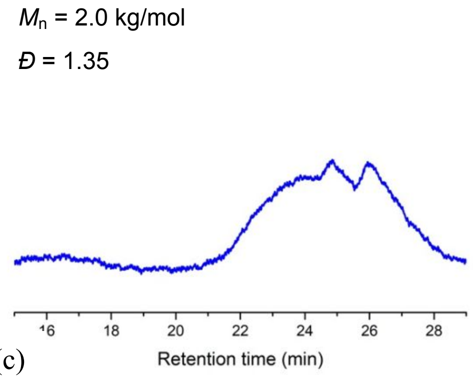

(b)

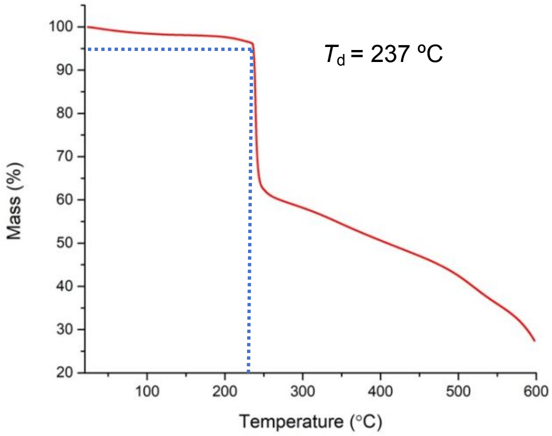

(c)

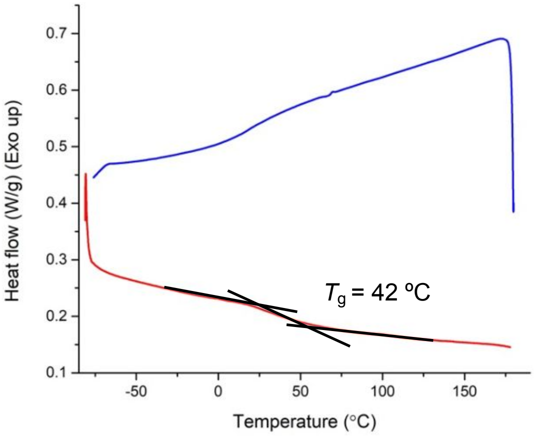

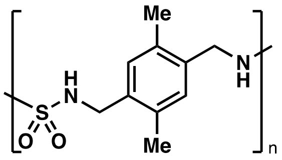

poly-IV-b

(a) SEC trace; **(b)** TGA curve; **(c)** DSC curves;

(a)

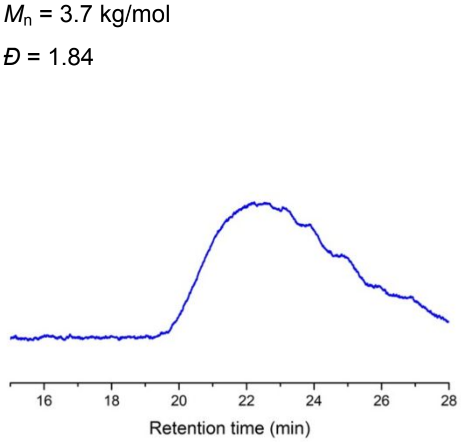

(b)

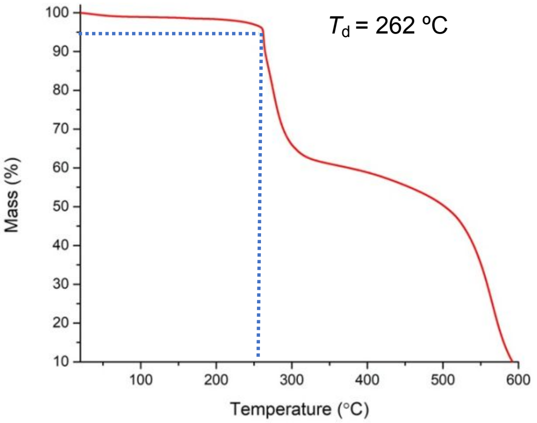

(c)

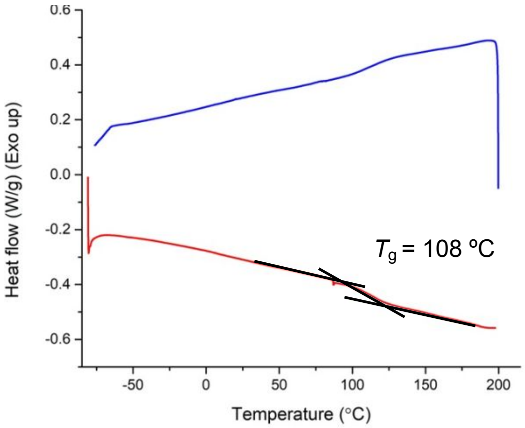

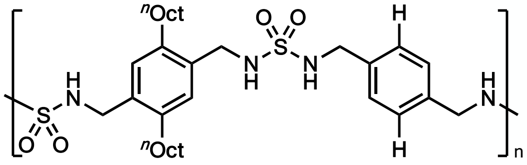

poly-IV-c

(a) SEC trace; **(b)** TGA curve; **(c)** DSC curves;

(a)

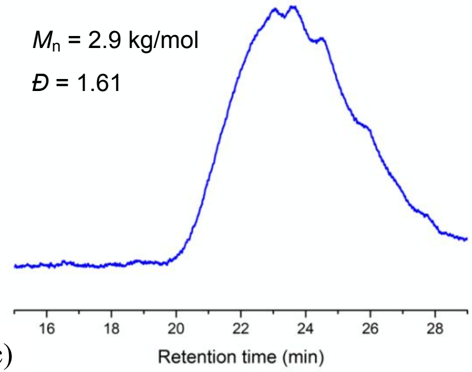

(b)

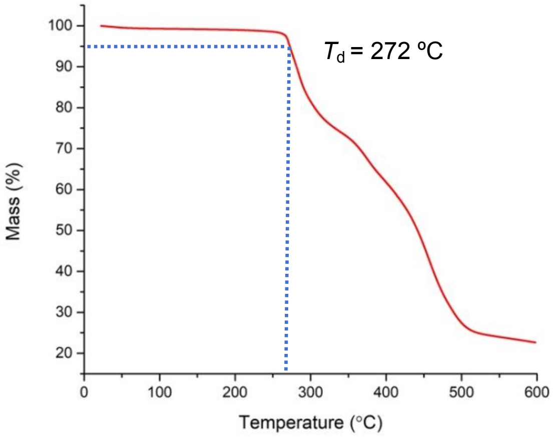

(c)

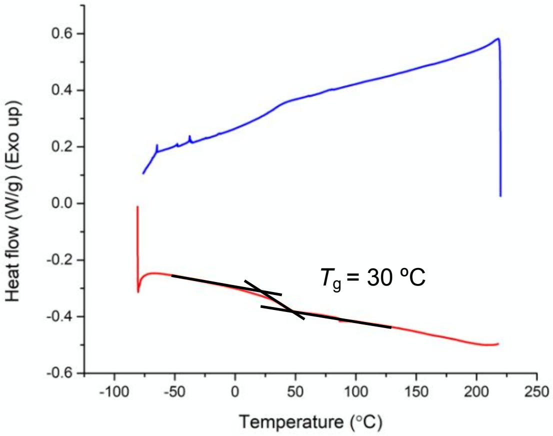

NMR spectra of all synthesized compounds

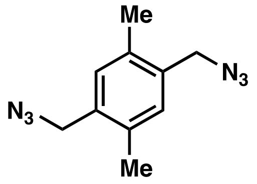

S9

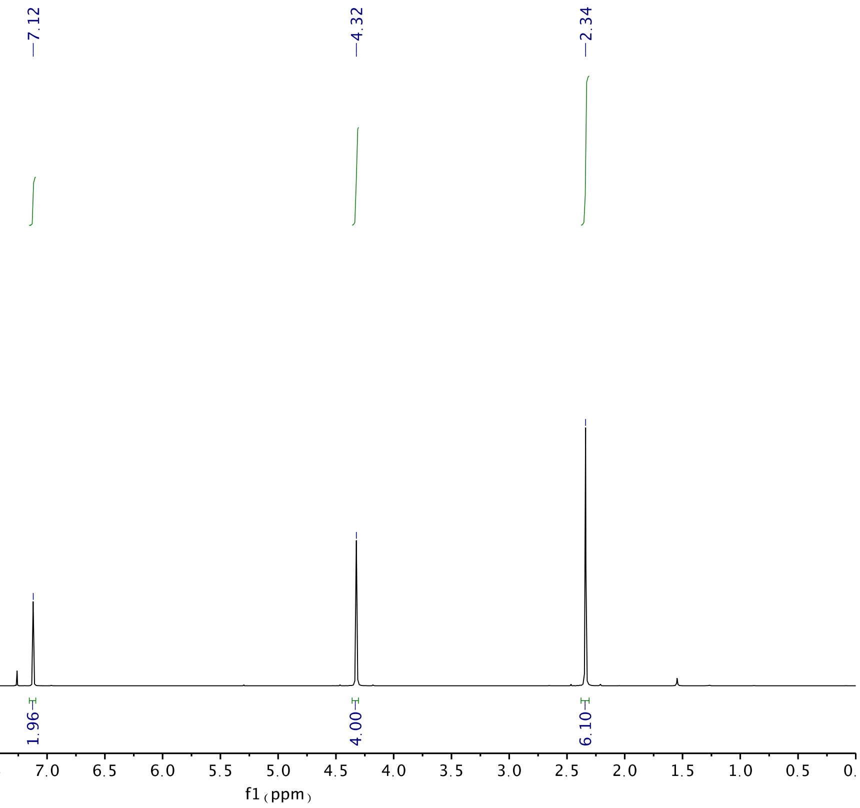

(^{1}H, 500 MHz, $CDCl_3$)

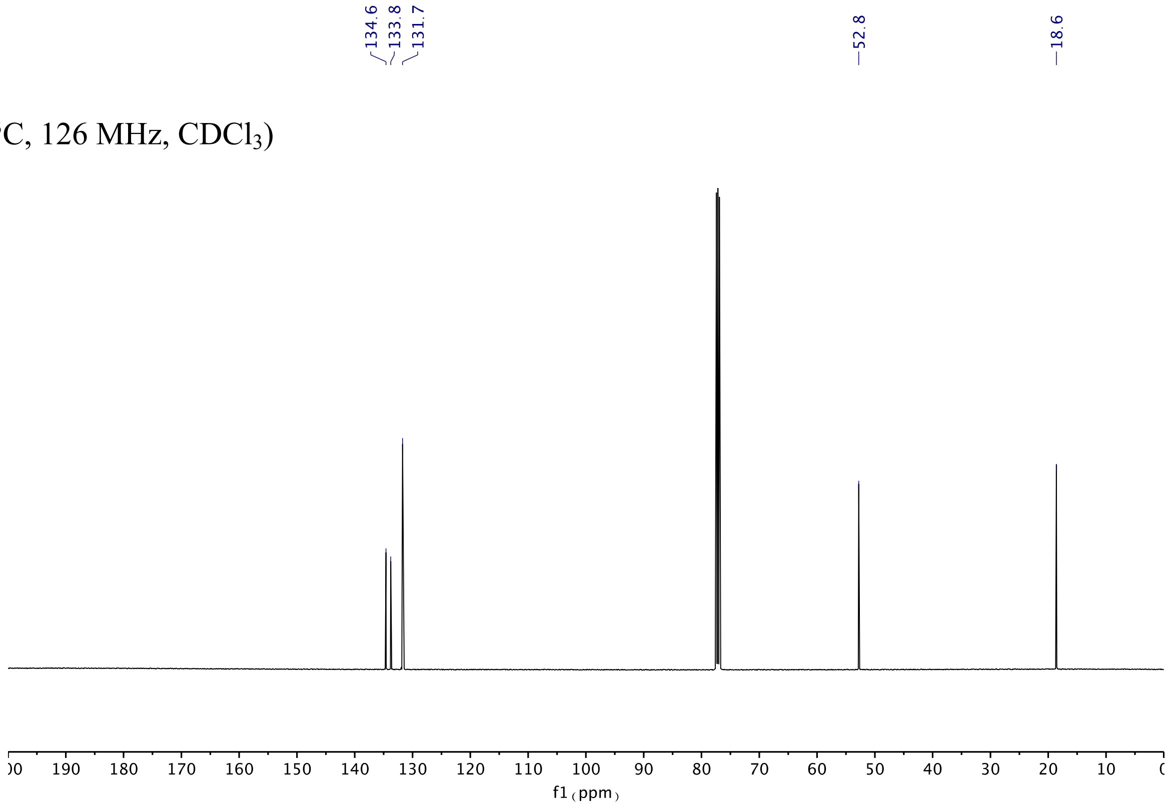

(^{13}C, 126 MHz, $CDCl_3$)

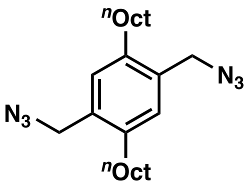

S10

(^{1}H, 500 MHz, $CDCl_3$)

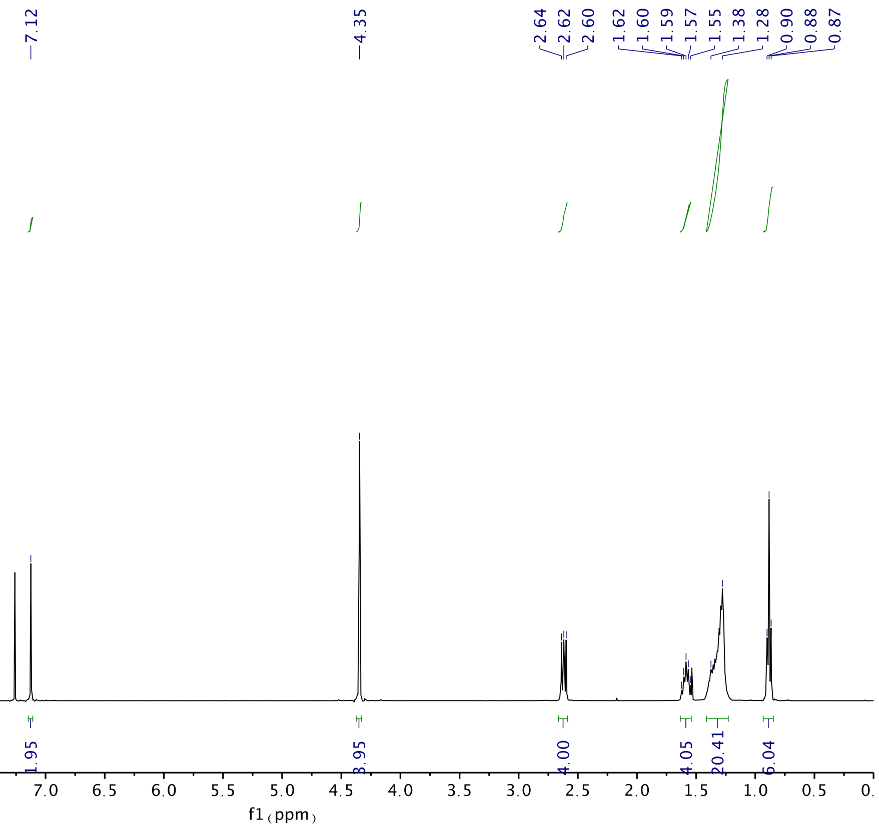

(^{13}C, 126 MHz, $CDCl_3$)

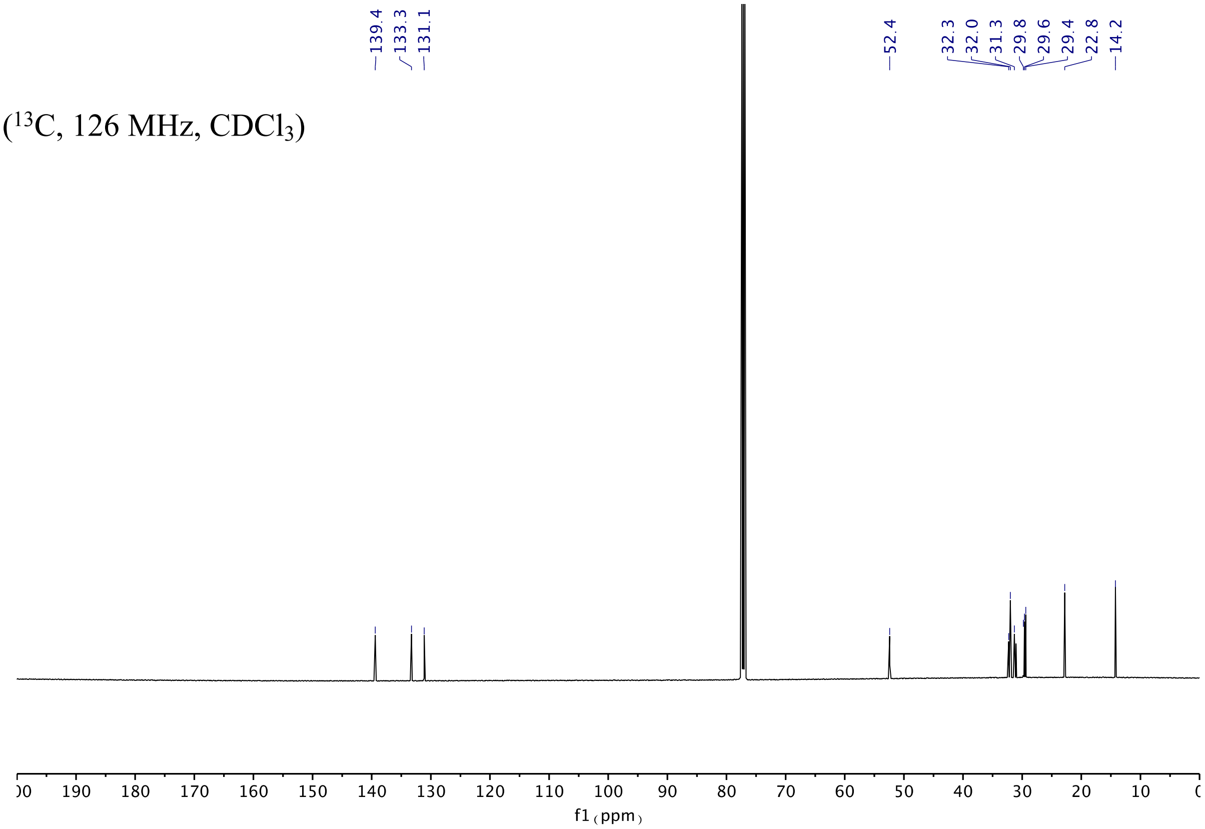

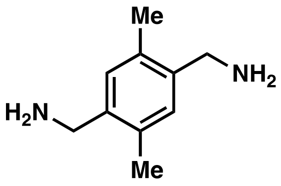

S5

(^{1}H, 500 MHz, $CDCl_3$)

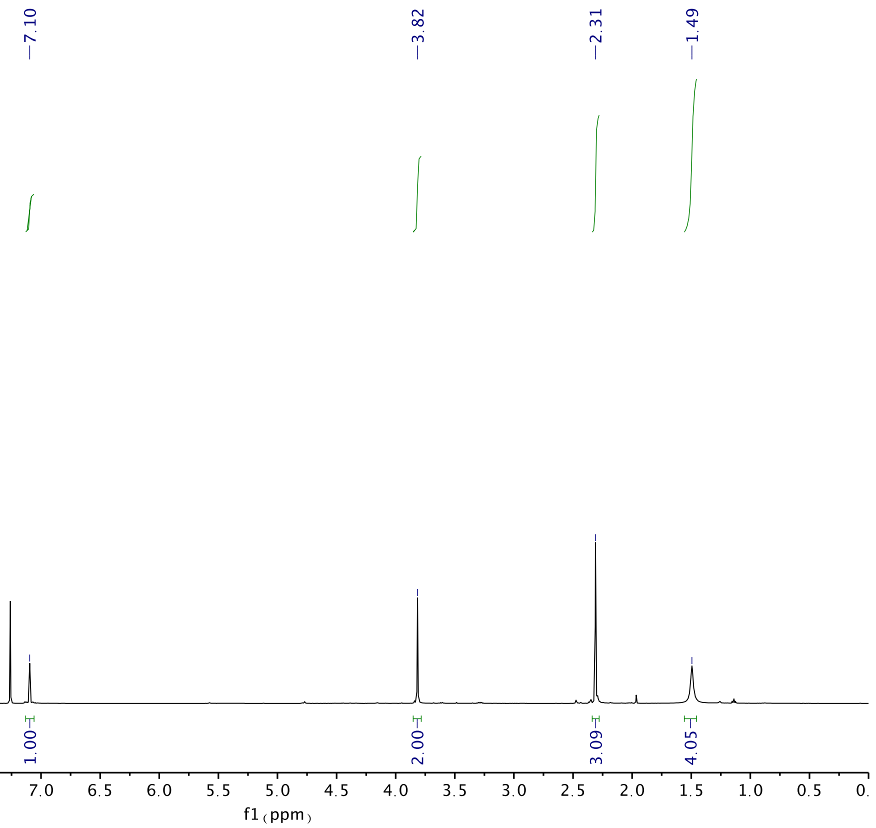

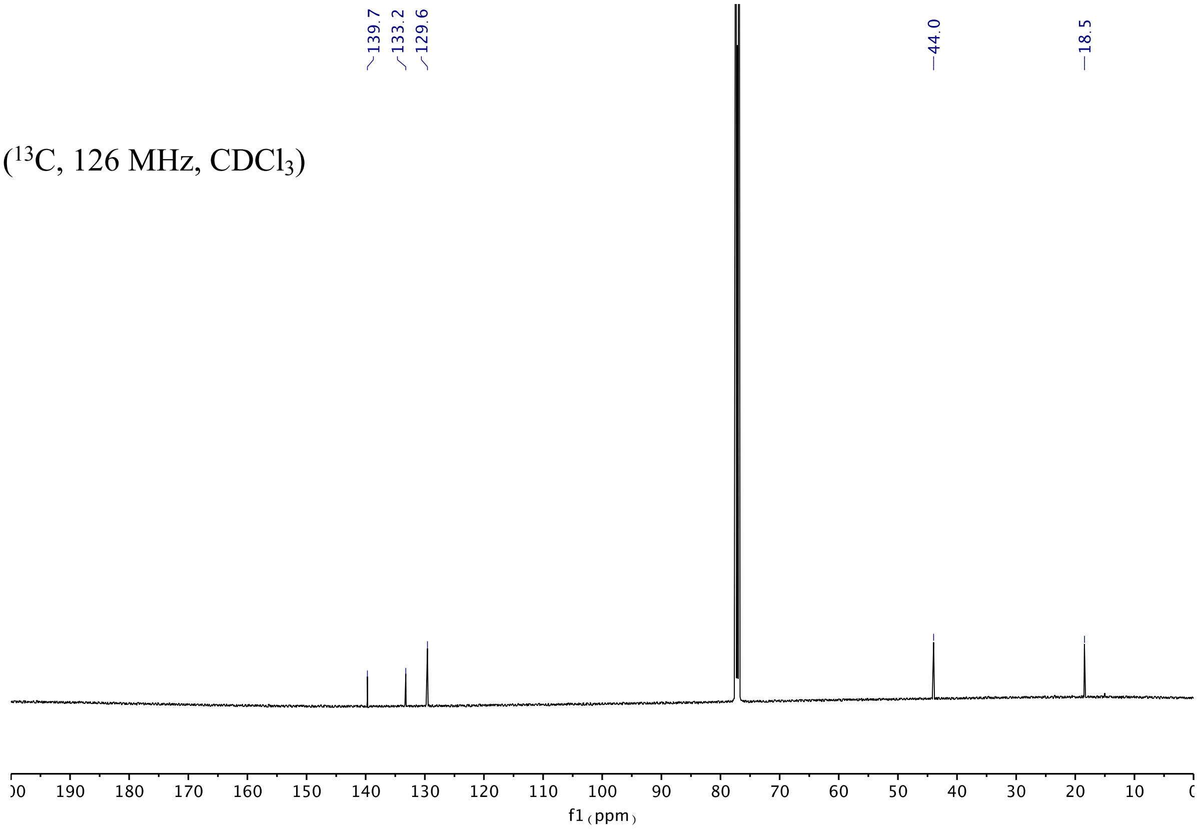

(^{13}C, 126 MHz, $CDCl_3$)

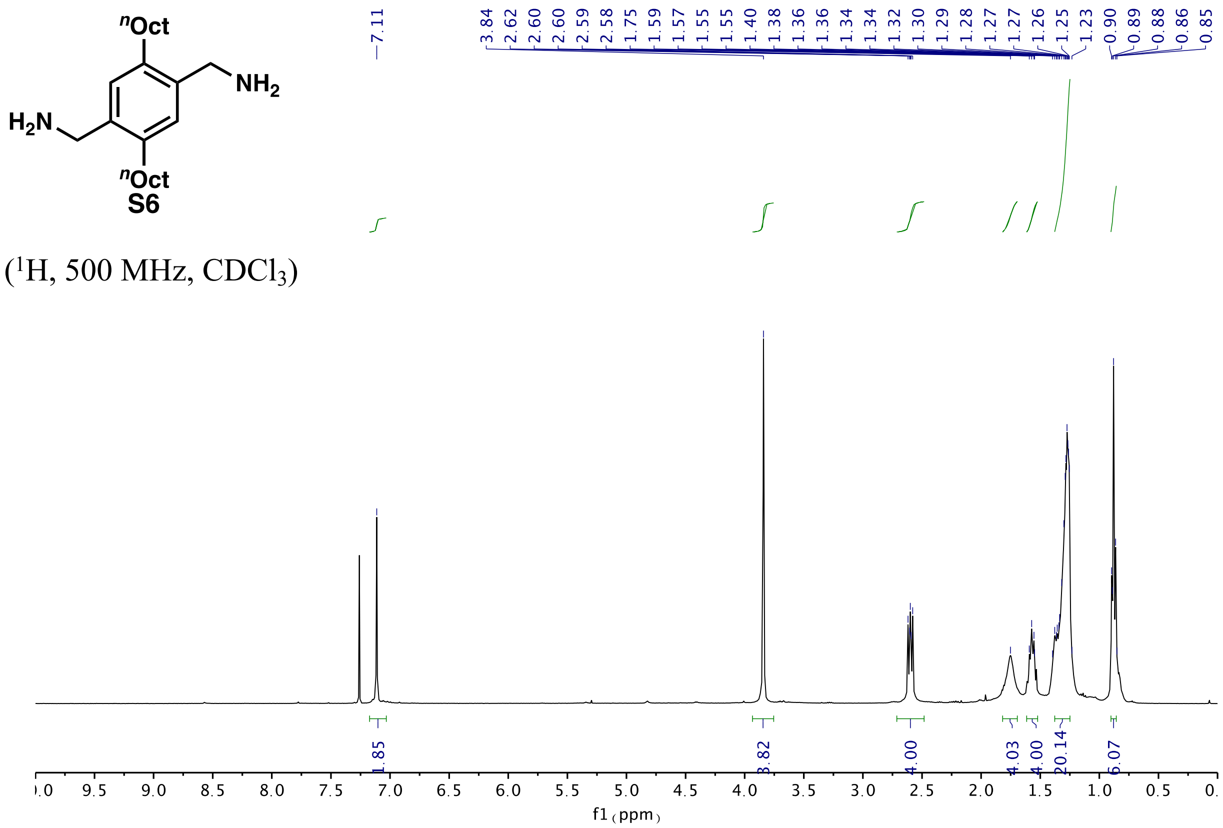

S6

(^{1}H, 500 MHz, $CDCl_3$)

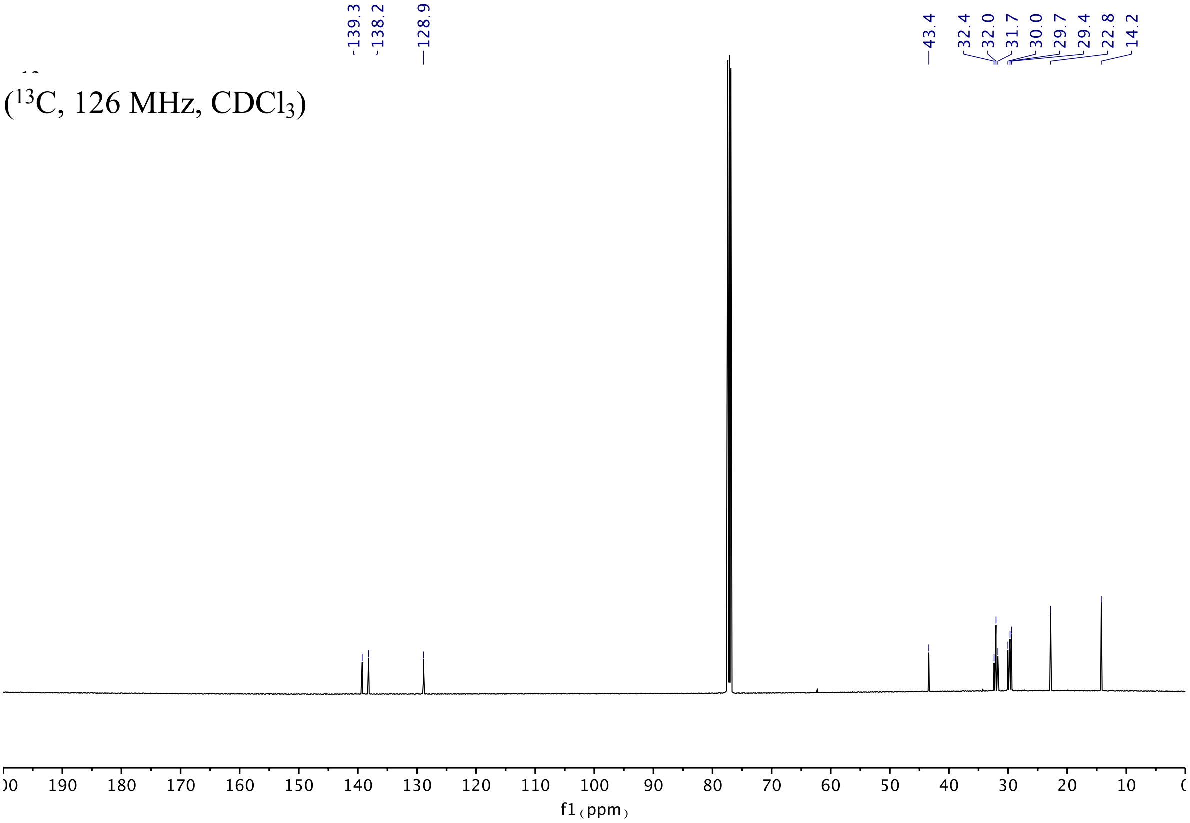

(^{13}C, 126 MHz, $CDCl_3$)

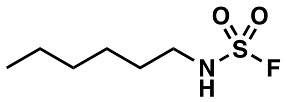

S12

(^{1}H, 500 MHz, $CDCl_3$)

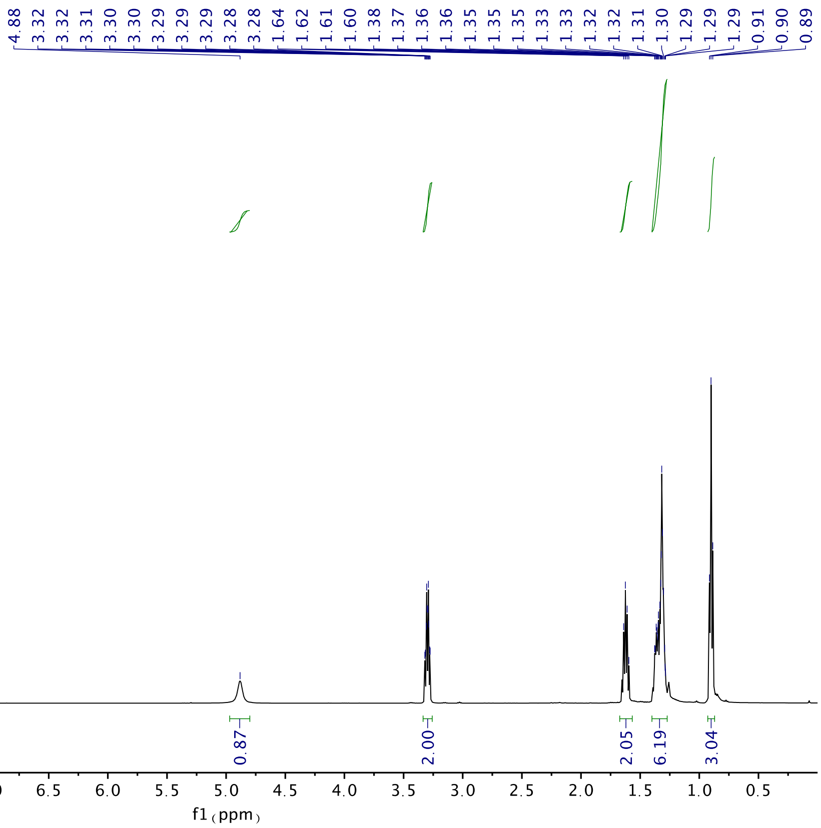

(^{13}C, 126 MHz, $CDCl_3$)

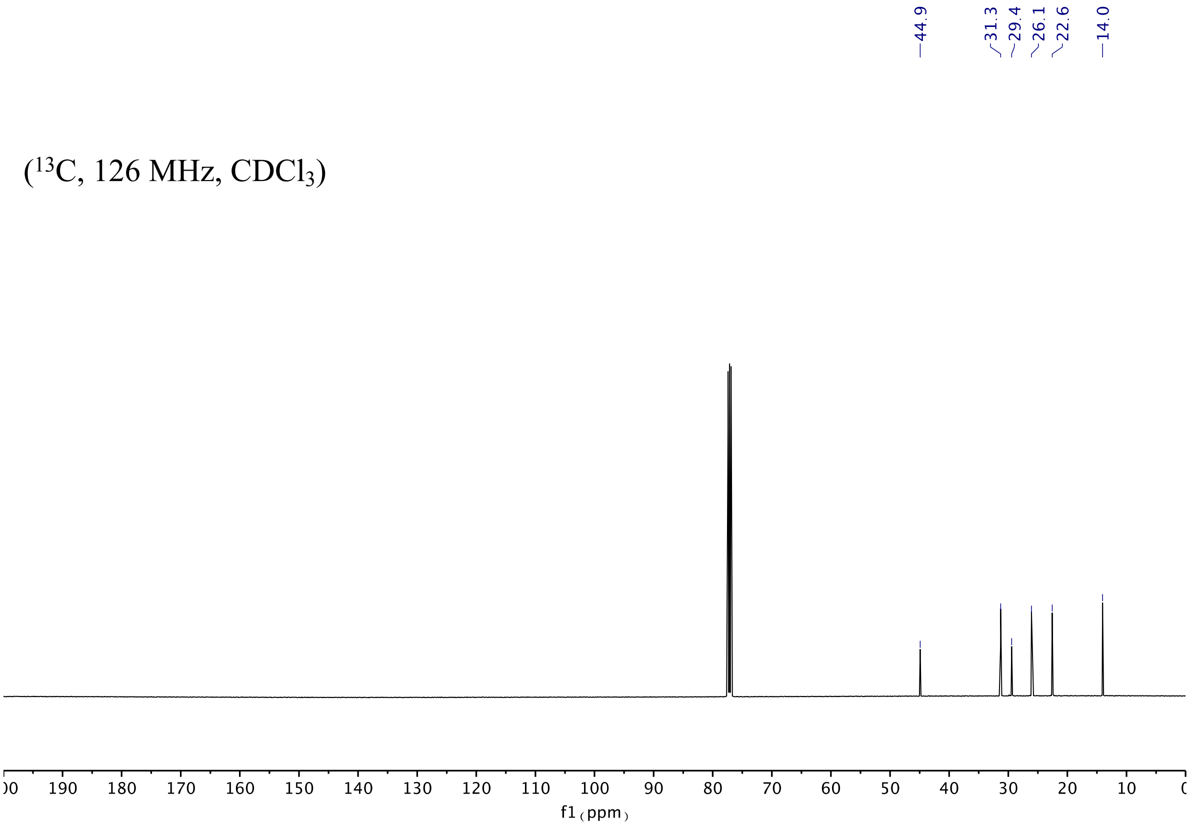

(^{19}F, 470 MHz, $CDCl_3$)

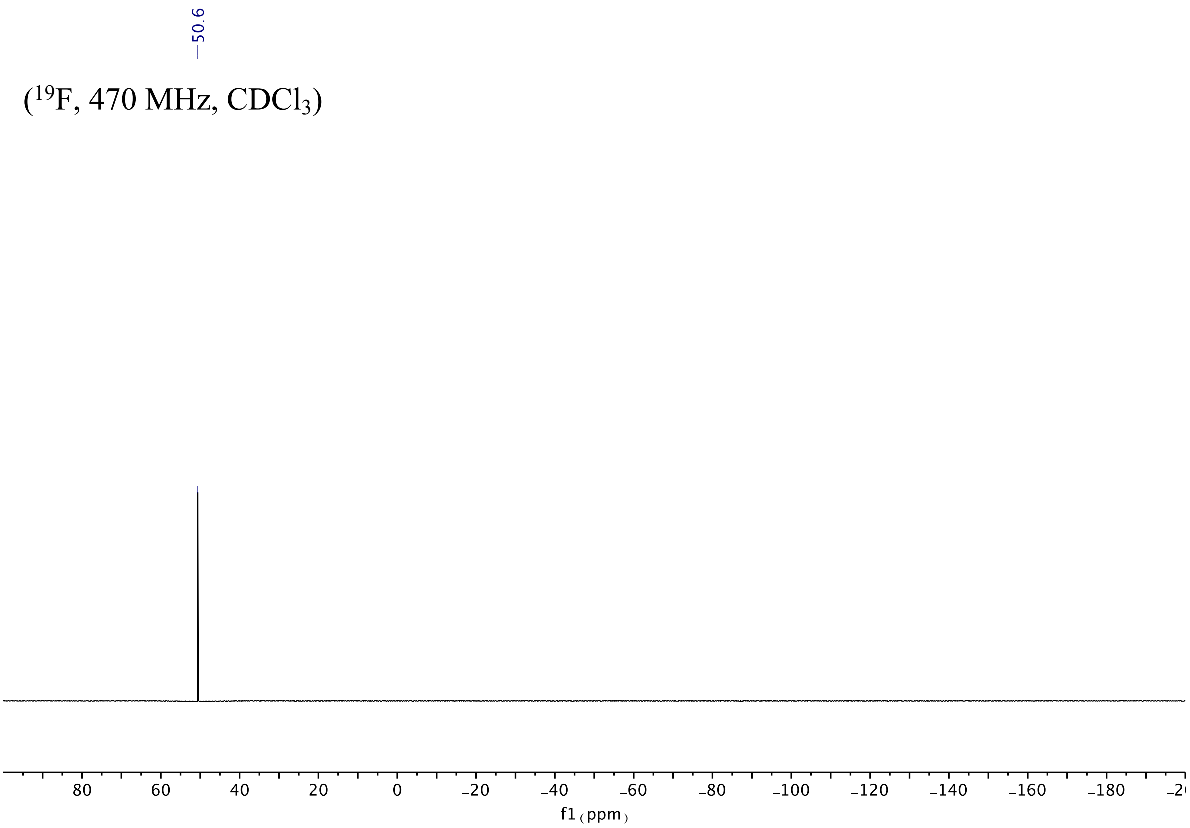

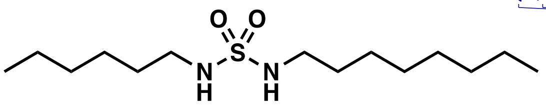

Sulf-3

(^{1}H, 500 MHz, $CDCl_3$)

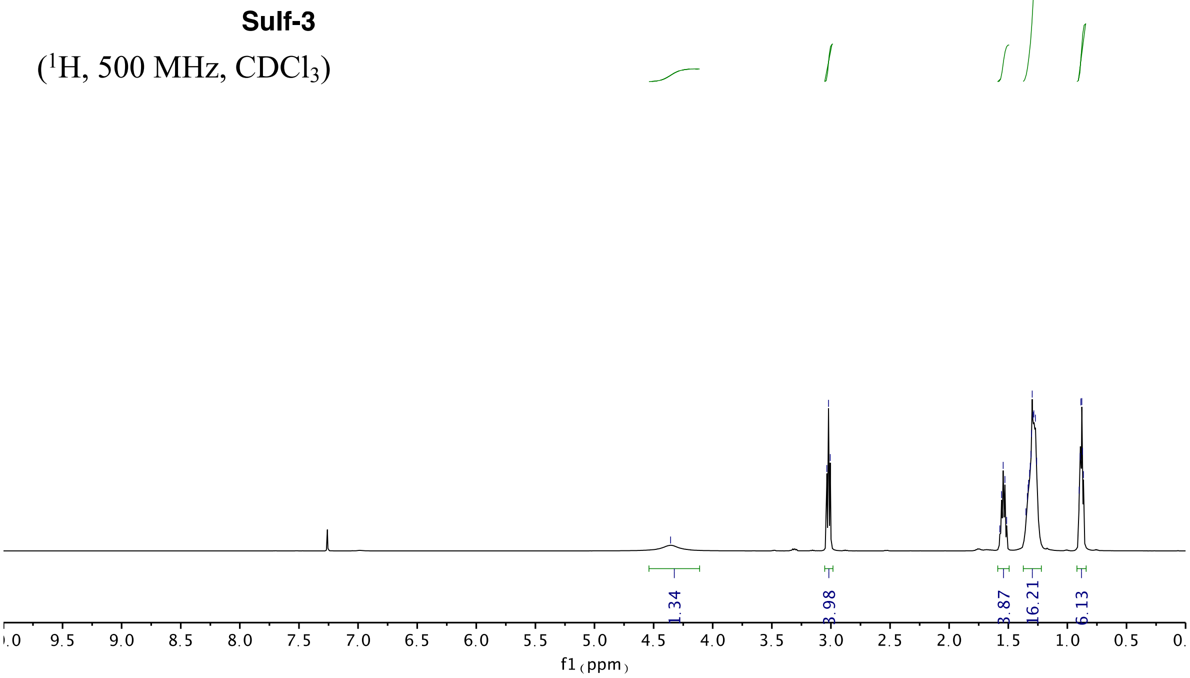

(^{13}C, 126 MHz, $CDCl_3$)

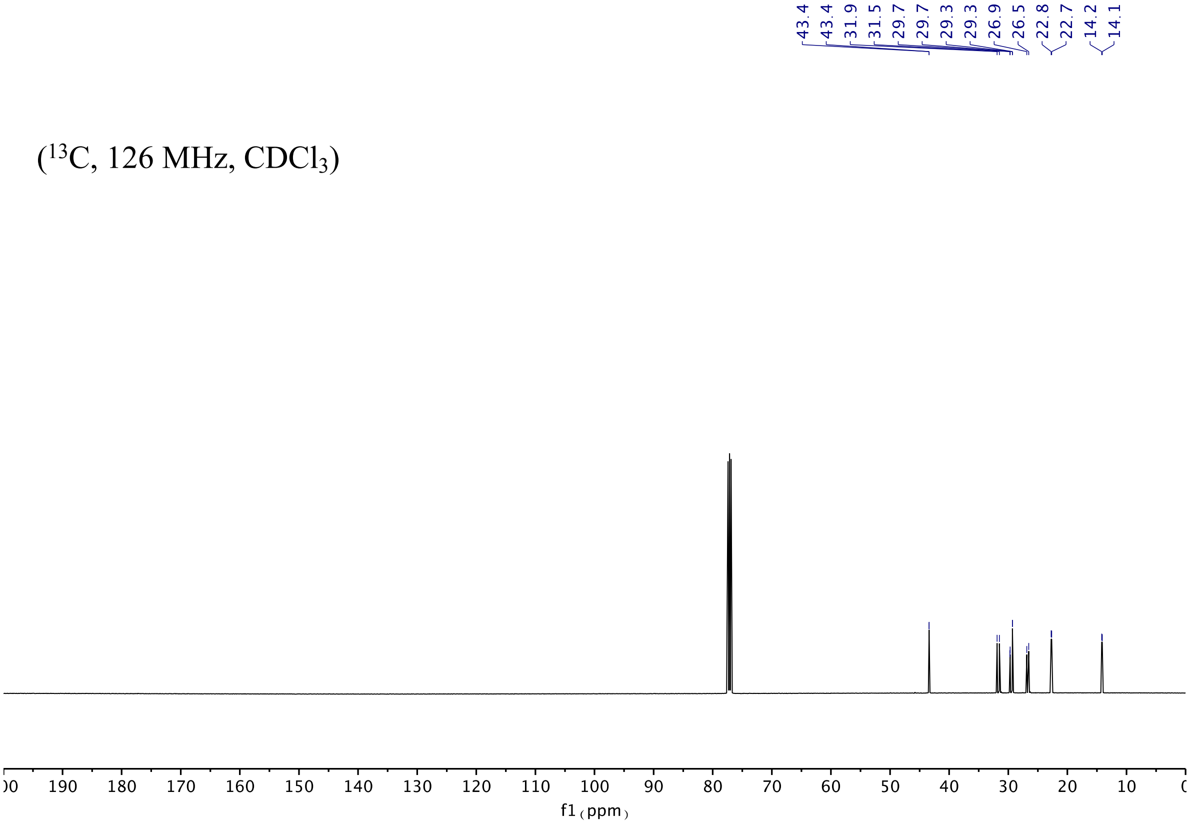

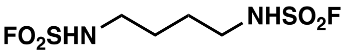

S1$_F$

(^{1}H, 500 MHz, CDCl$_3$)

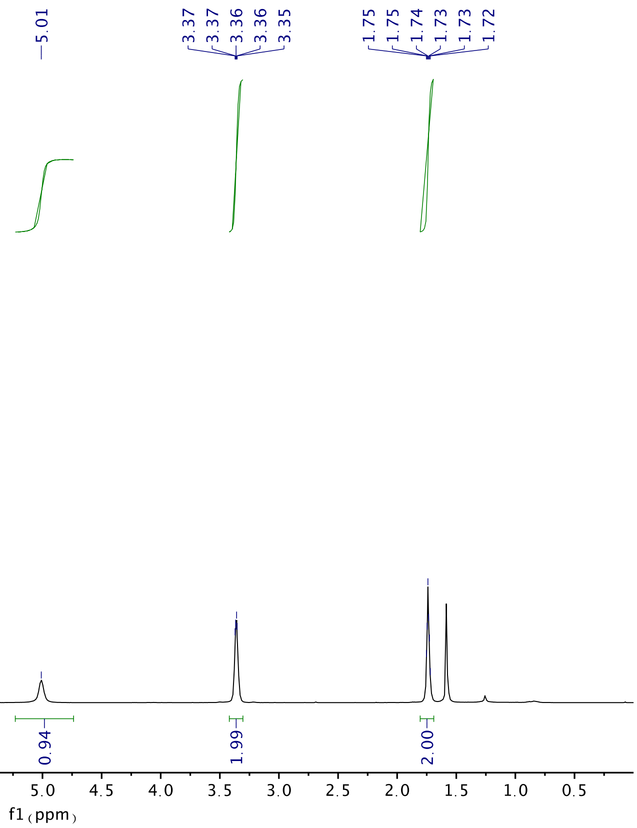

(^{13}C, 126 MHz, CDCl$_3$)

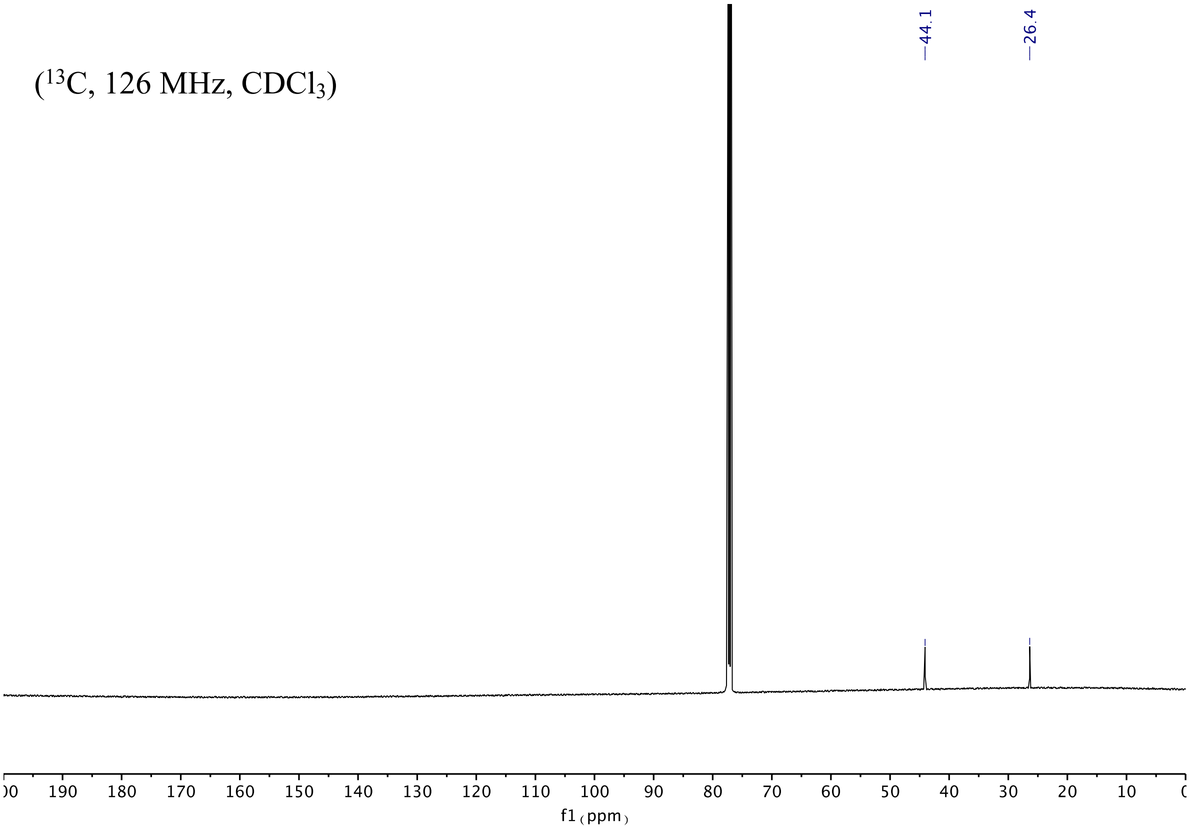

(${}^{19}F$, 470 MHz, $CDCl_3$)

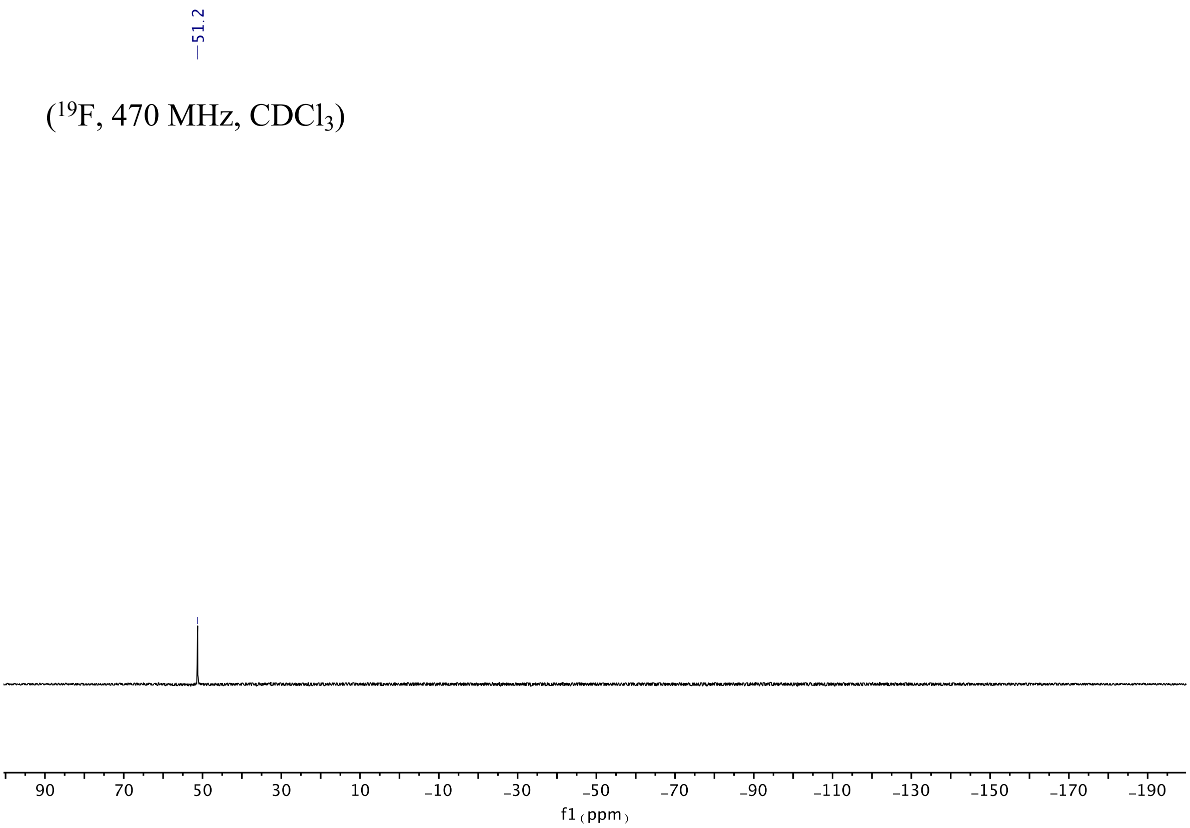

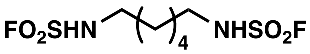

$S2_F$

(^1H, 500 MHz, $CDCl_3$)

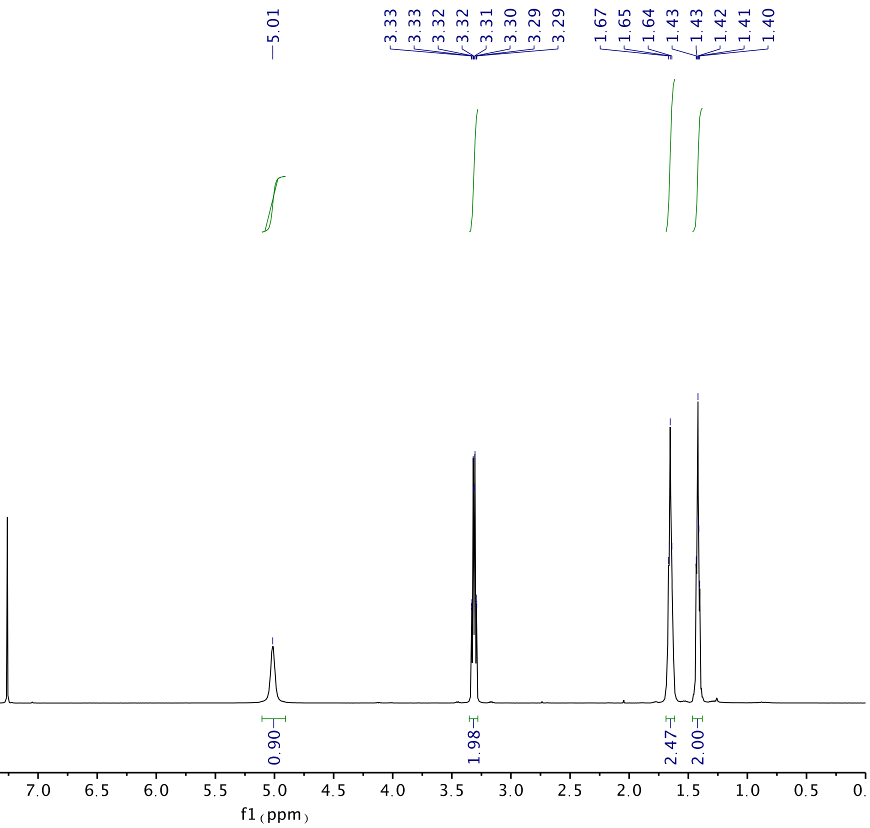

(^{13}C, 126 MHz, $CDCl_3$)

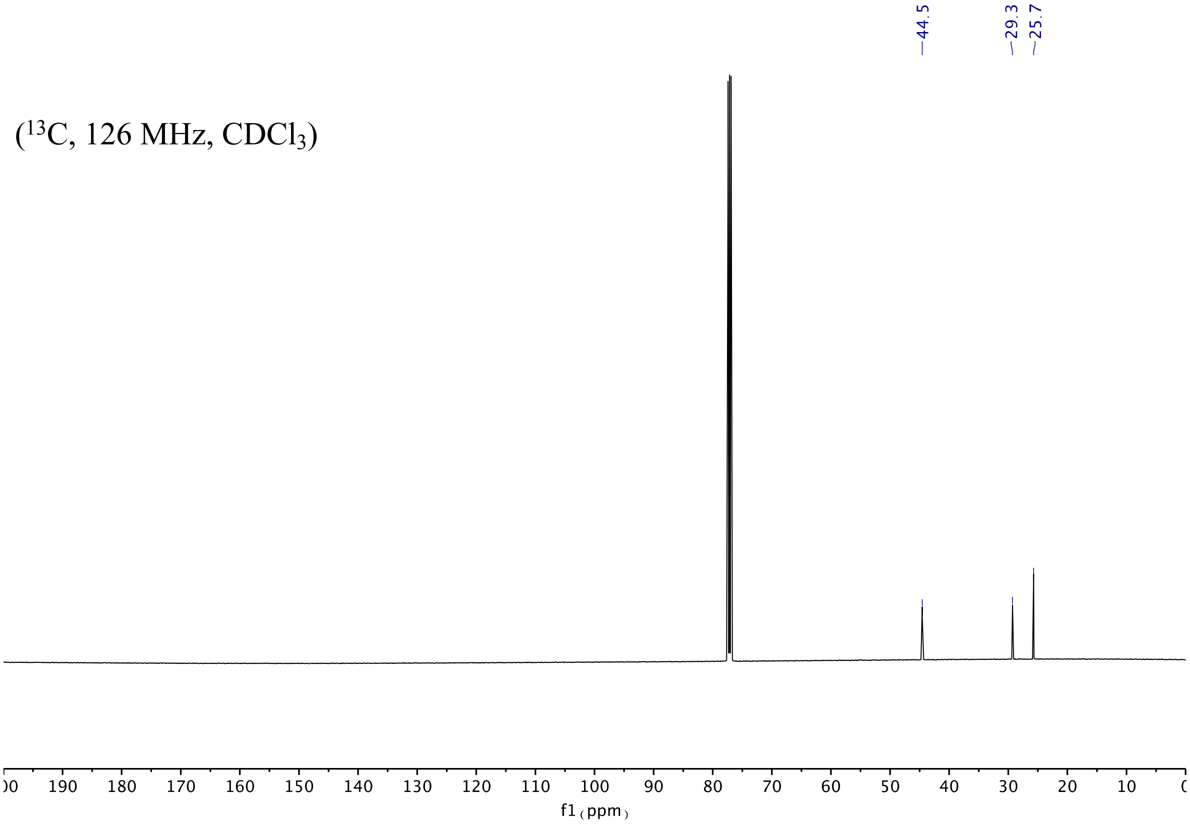

(^{19}F, 470 MHz, $CDCl_3$)

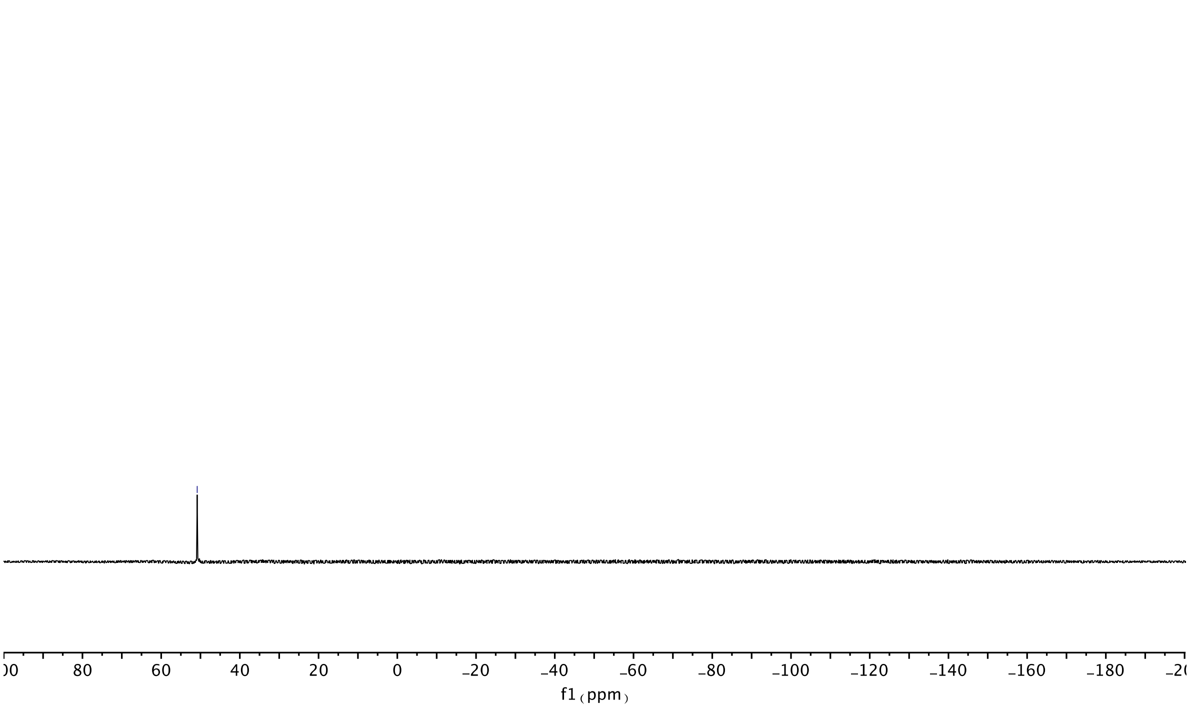

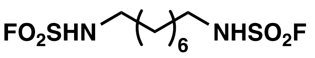

S3$_F$

(^{1}H, 500 MHz, $CDCl_3$)

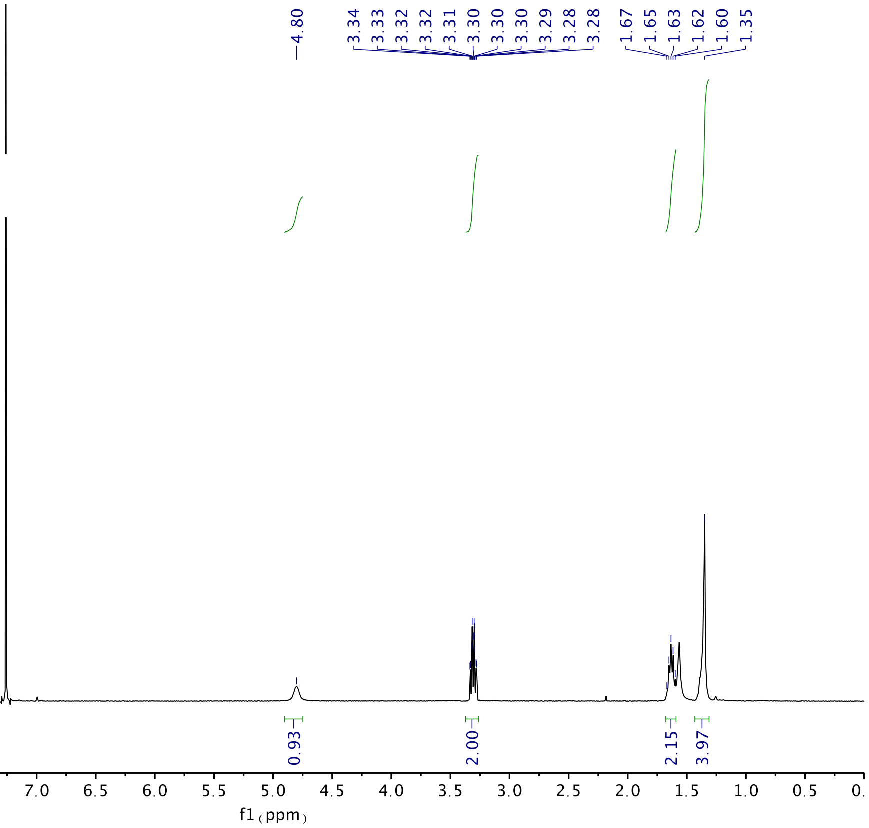

(^{13}C, 126 MHz, $CDCl_3$)

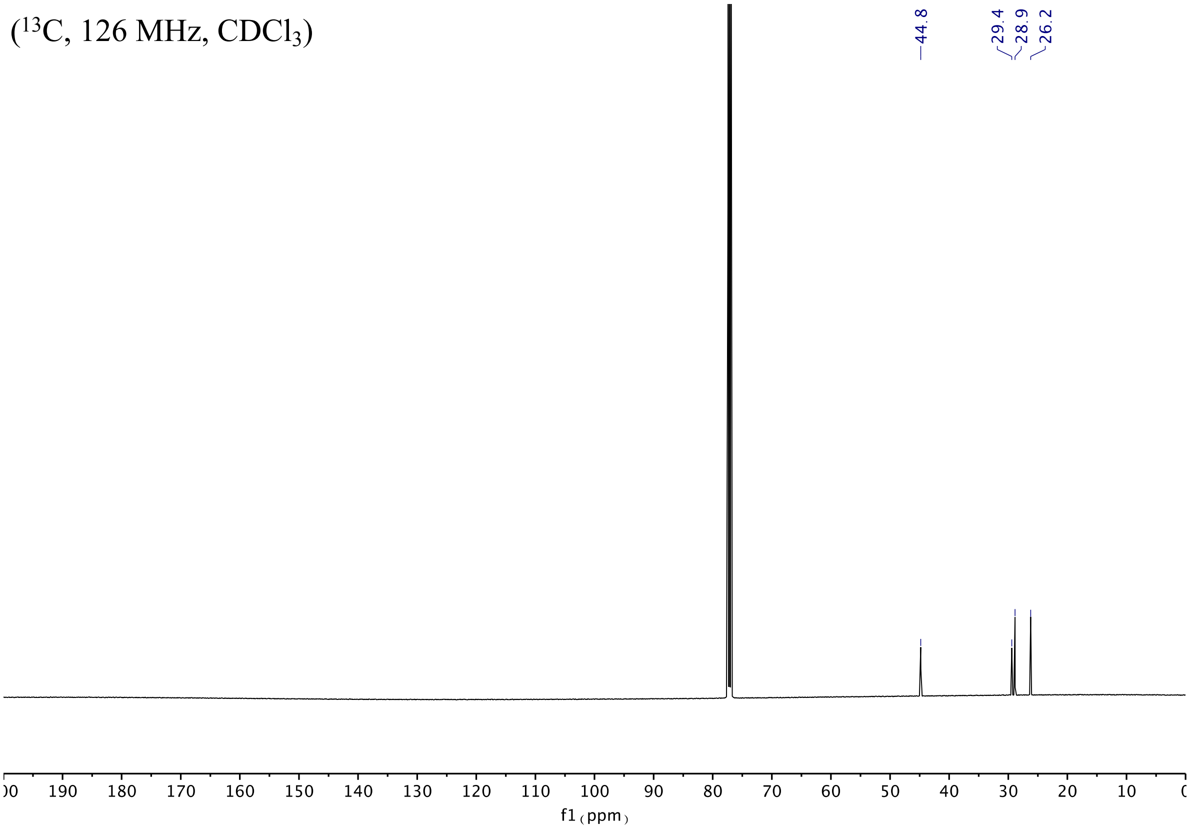

(^{19}F, 470 MHz, $CDCl_3$)

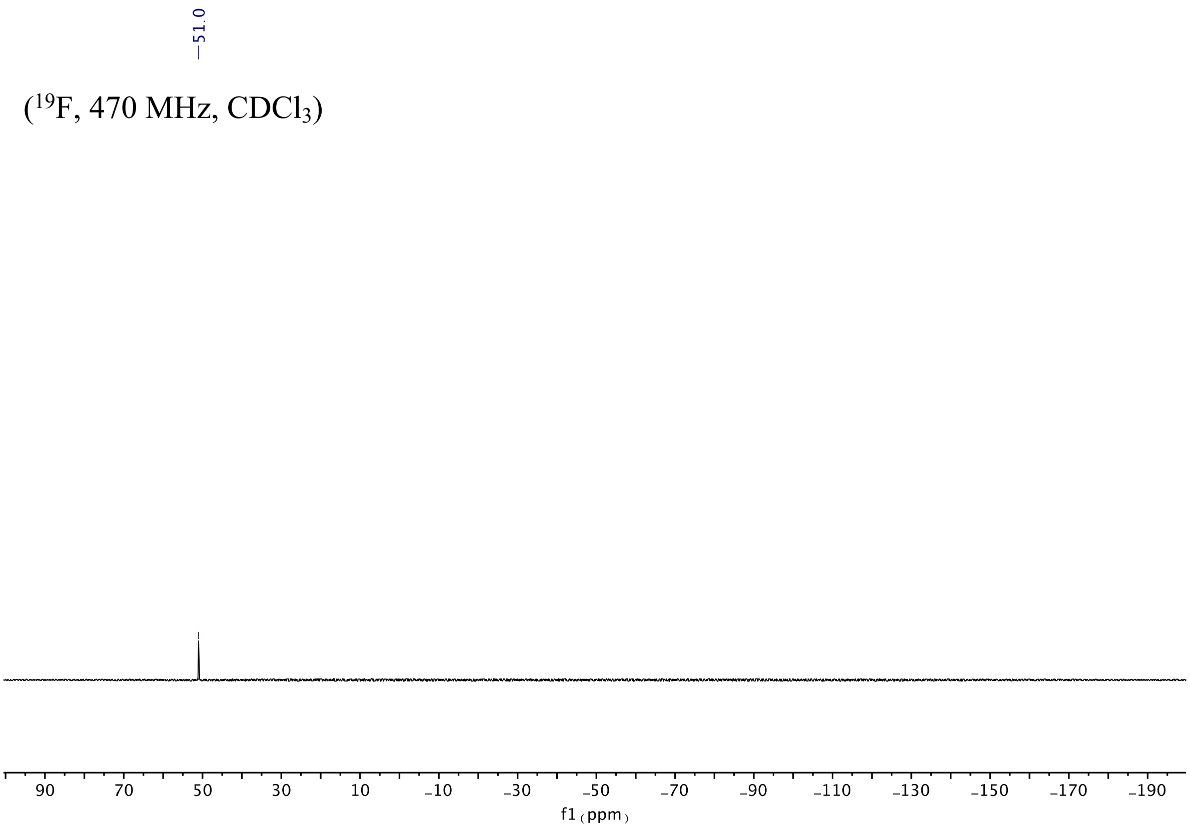

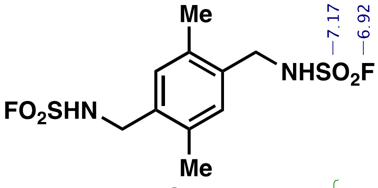

S5F

(^{1}H, 500 MHz, d_3-MeCN)

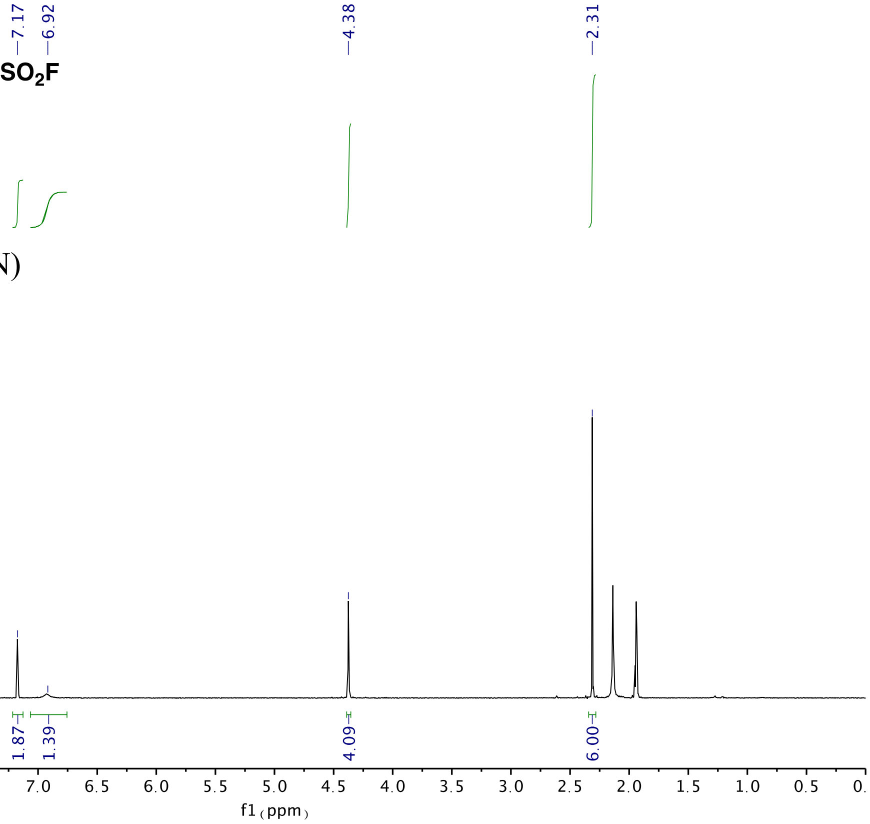

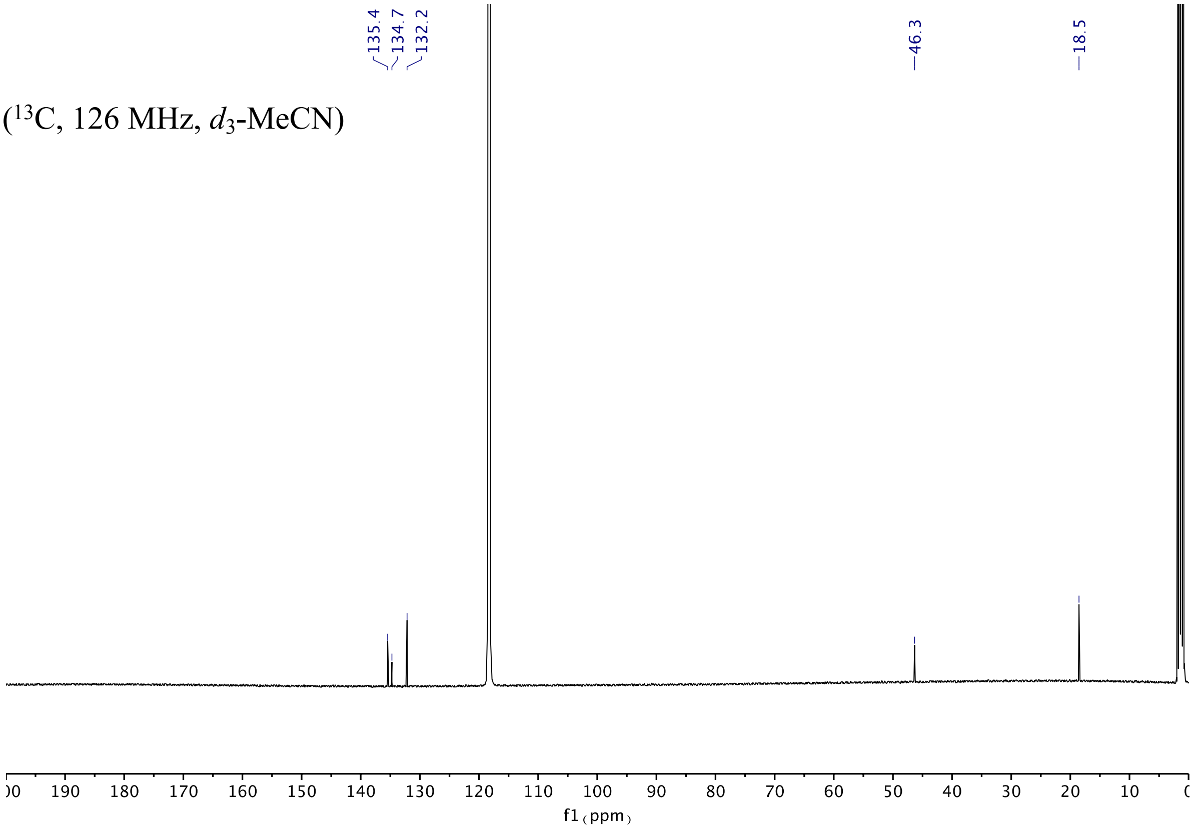

(^{13}C, 126 MHz, d_3-MeCN)

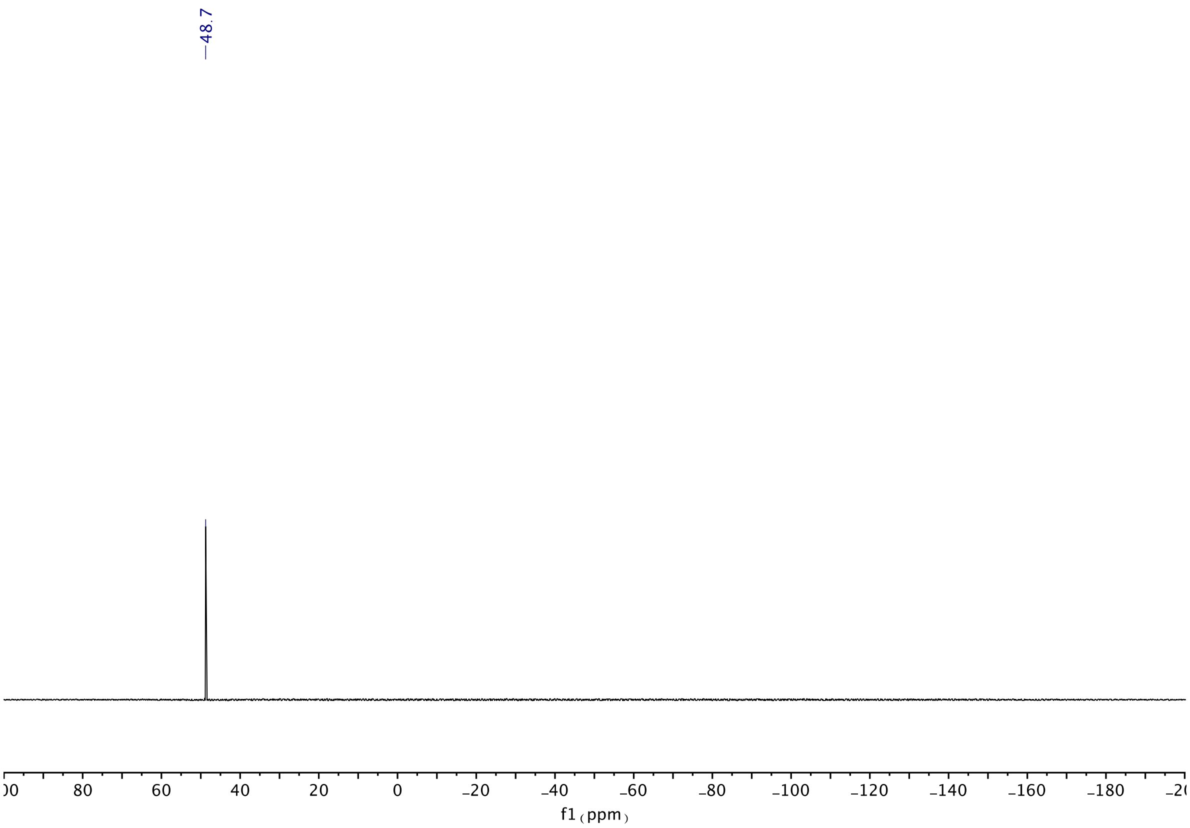

—48.7
00
80
60
40
20
0
-20
-40
-60
-80
-100
-120
-140
-160
-180
-2(
f1 (ppm)

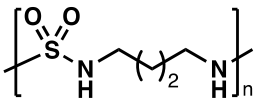

poly-II-a

(^{1}H, 500 MHz, d_6-DMSO)

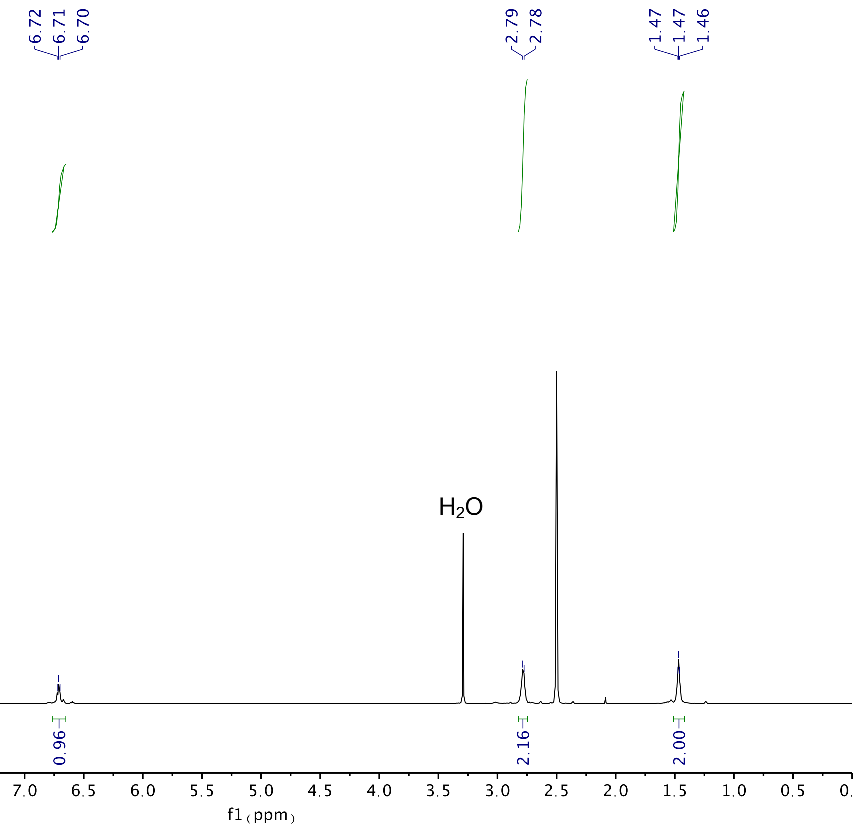

(^{13}C, 126 MHz, d_6-DMSO)

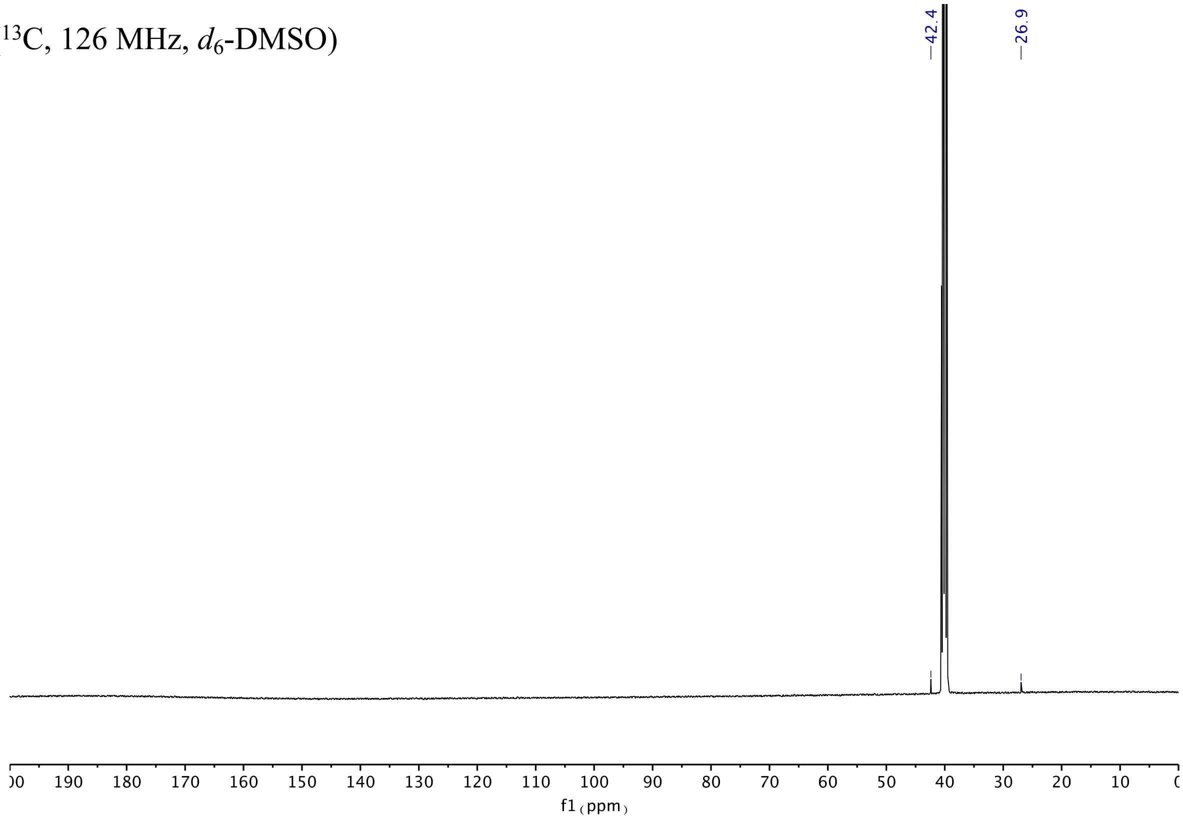

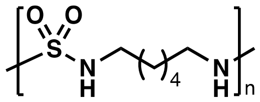

poly-II-b

(^{1}H, 500 MHz, d_6-DMSO)

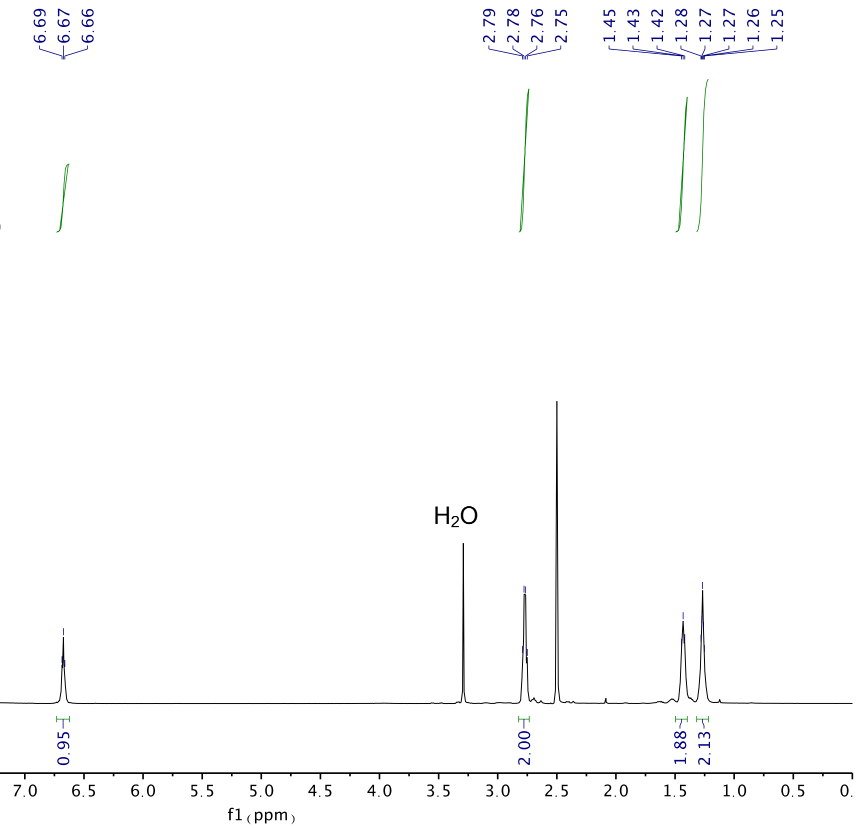

(^{13}C, 126 MHz, d_6-DMSO)

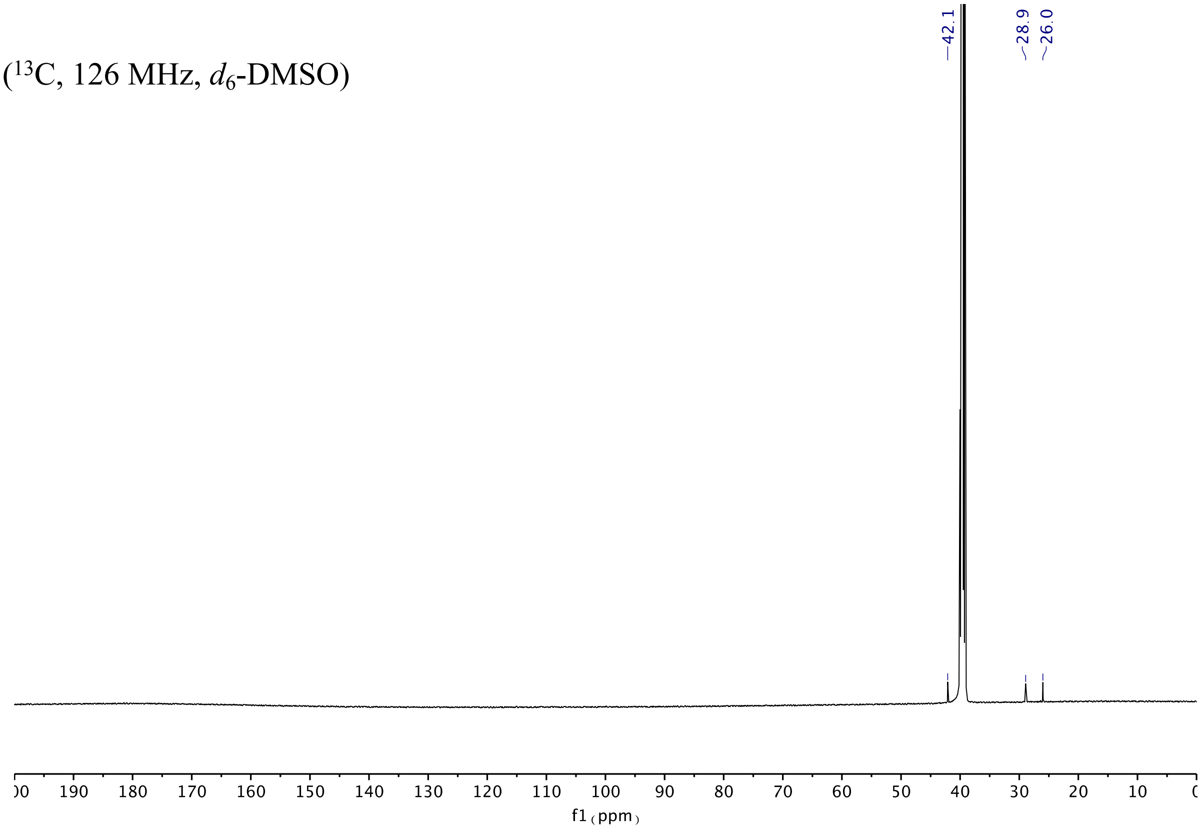

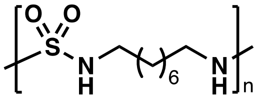

poly-II-c

(^{1}H, 500 MHz, d_6-DMSO)

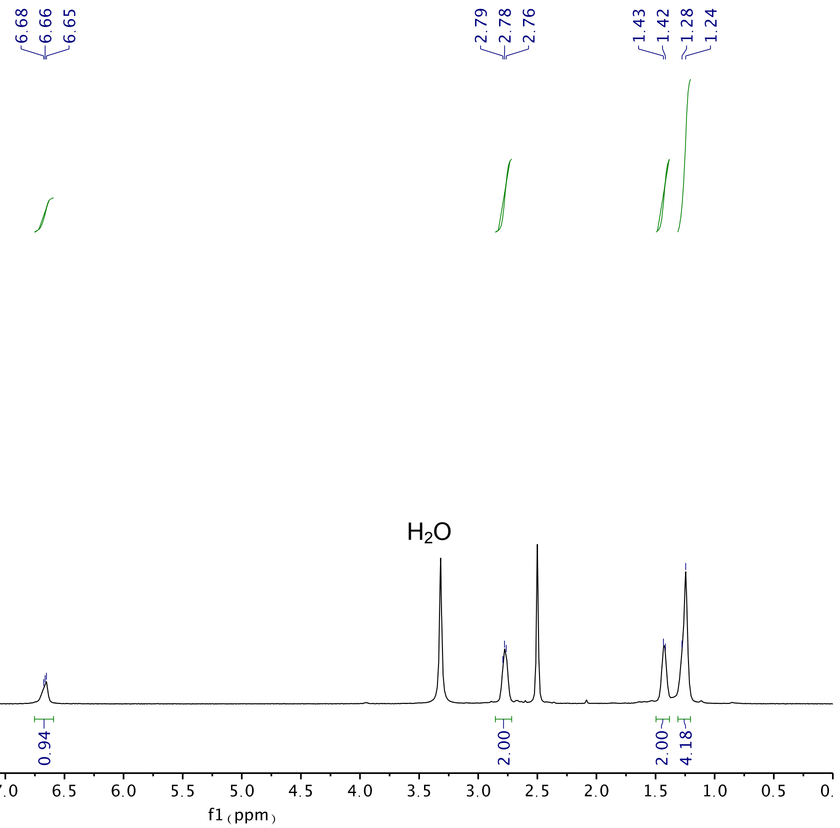

(^{13}C, 126 MHz, d_6-DMSO)

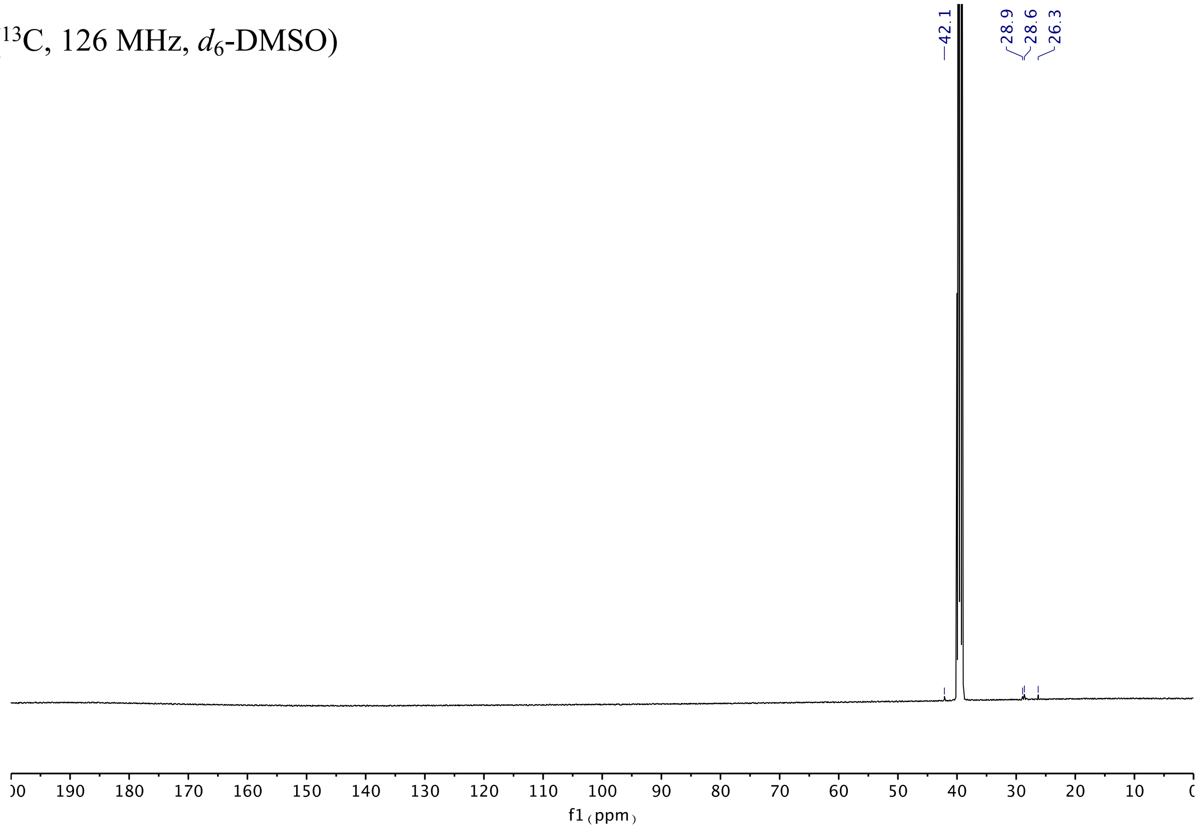

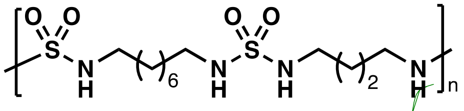

poly-III-a

(^{1}H, 500 MHz, d_6-DMSO)

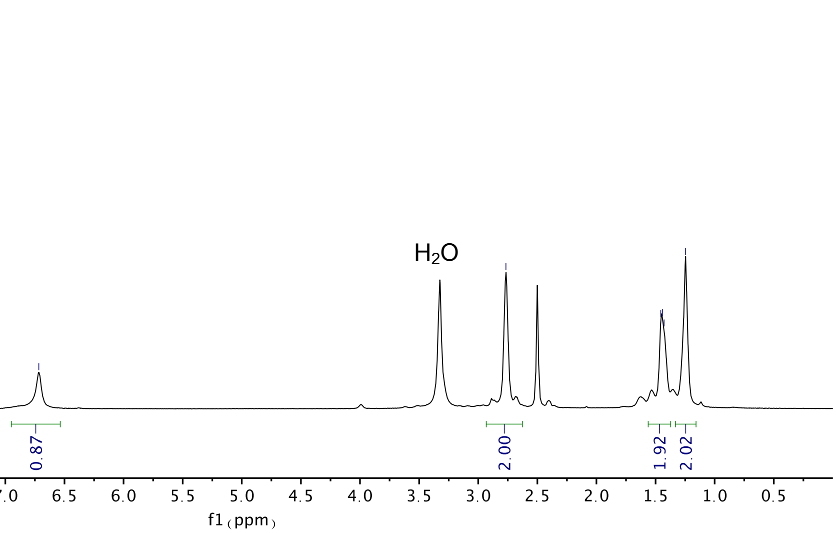

(^{13}C, 126 MHz, d_6-DMSO)

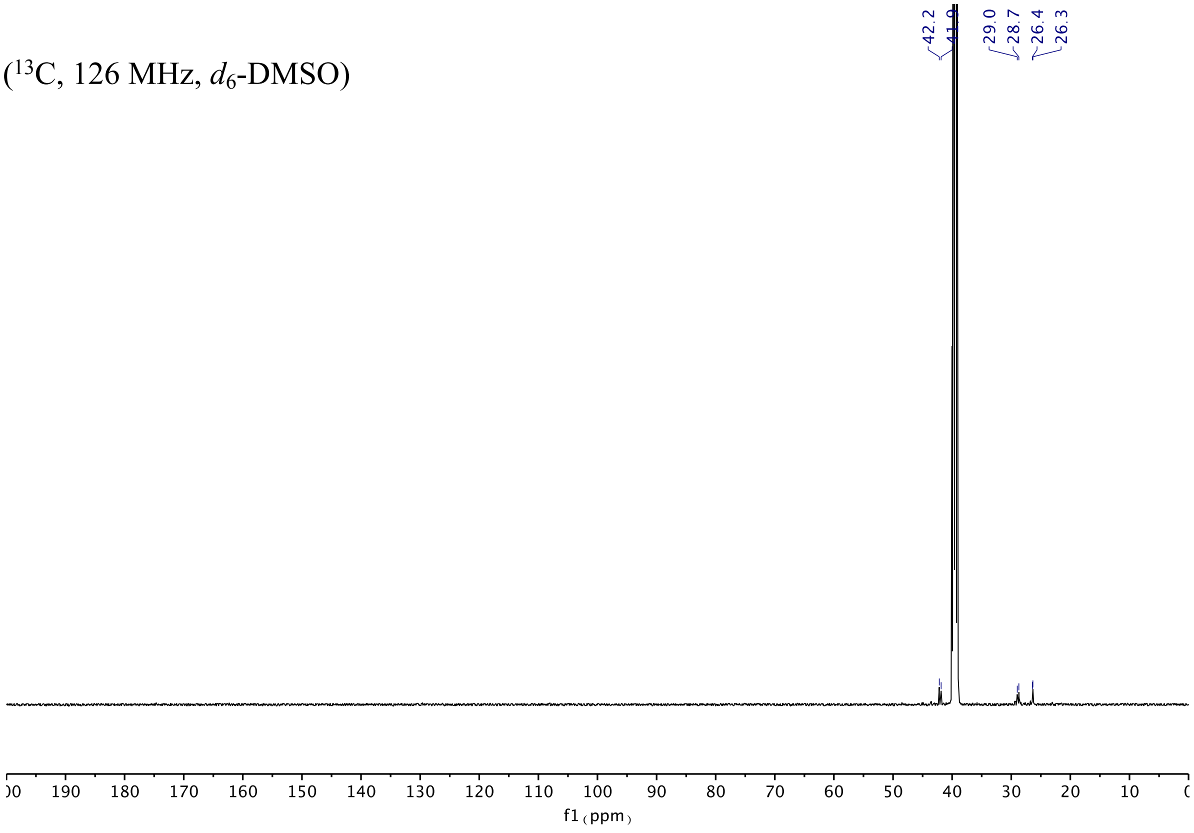

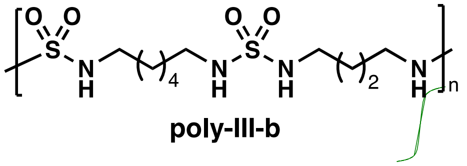

poly-III-b

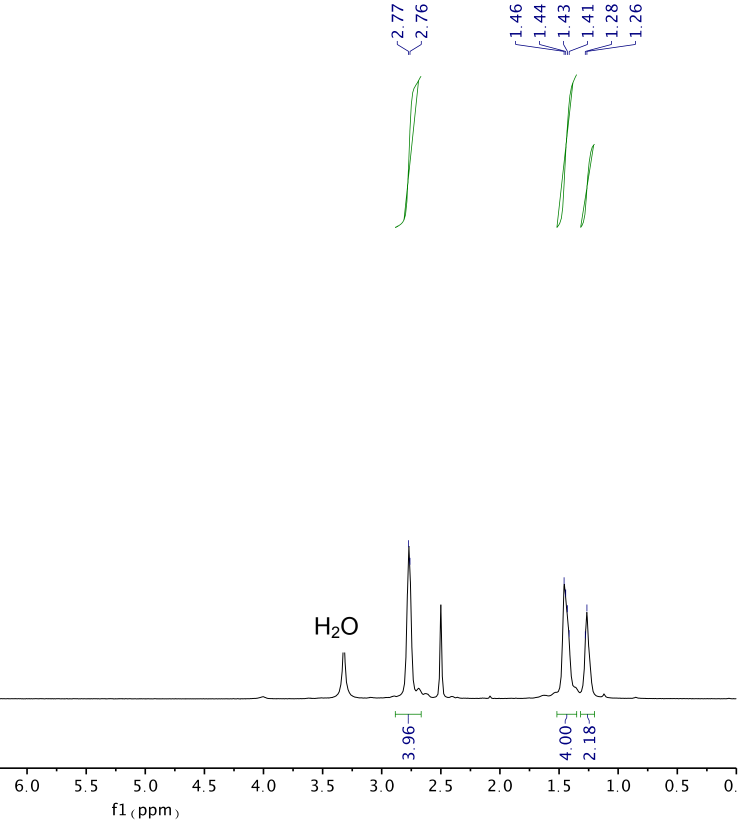

(^{1}H, 500 MHz, d_6-DMSO)

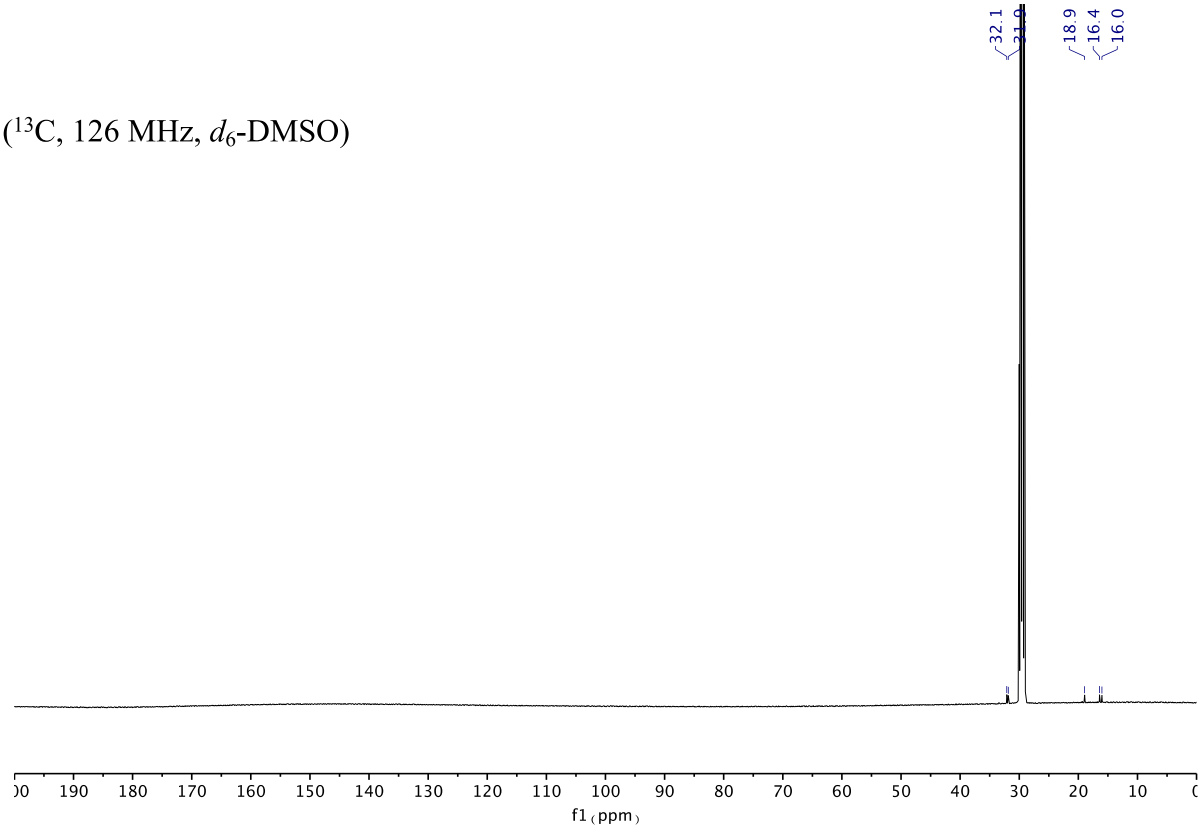

(^{13}C, 126 MHz, d_6-DMSO)

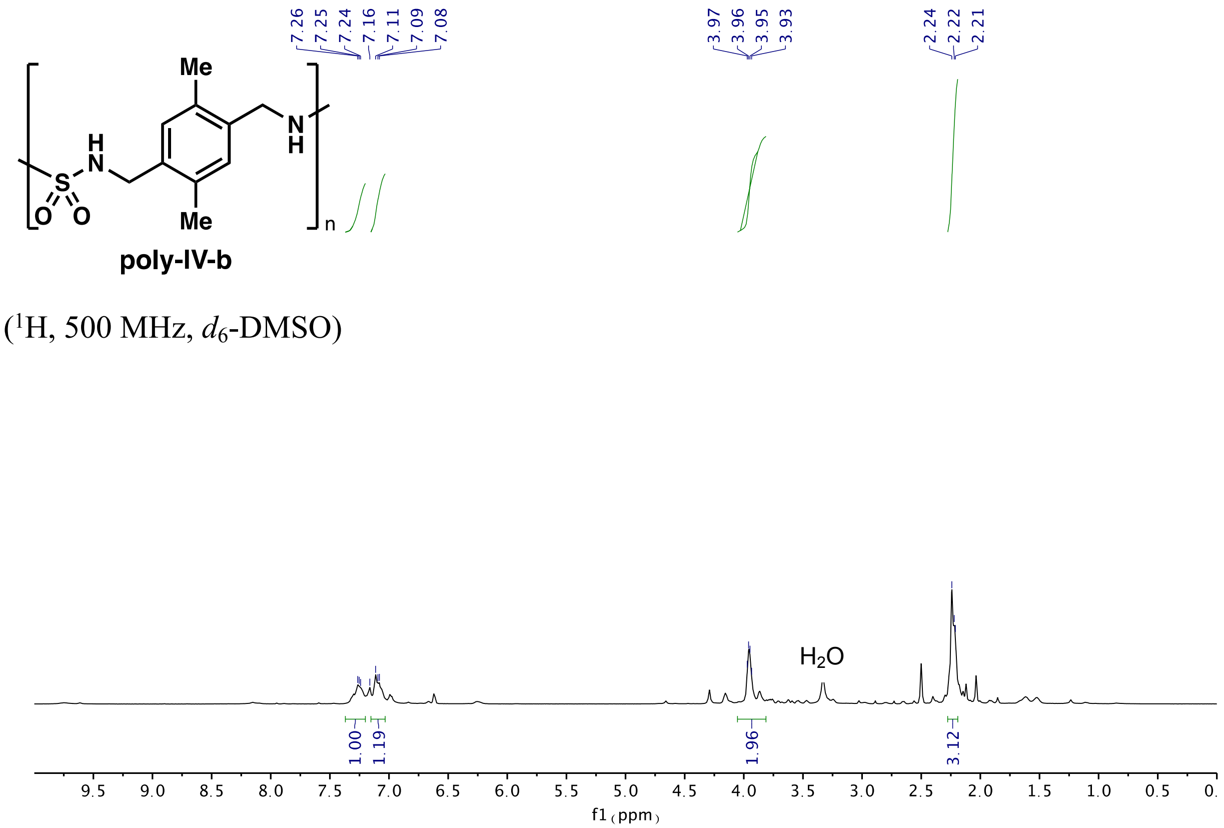

(^{1}H, 500 MHz, d_6-DMSO)

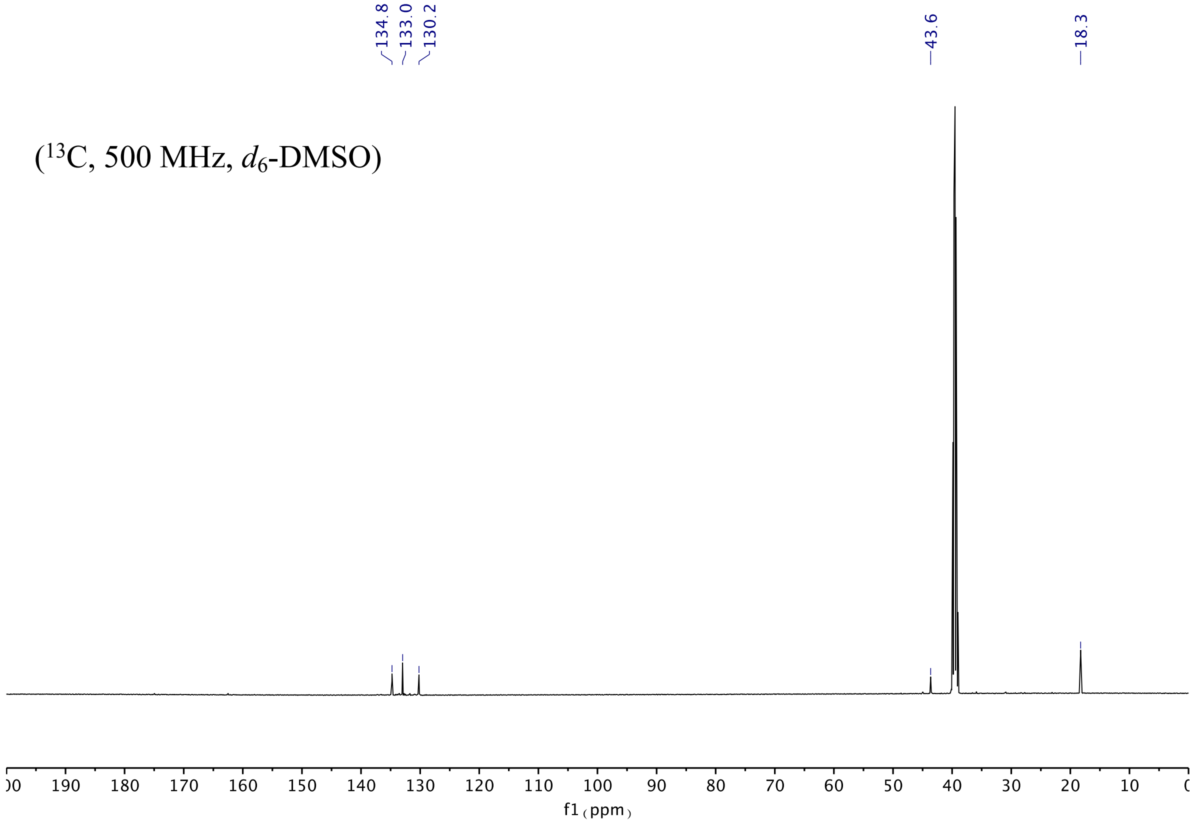

(^{13}C, 500 MHz, d_6-DMSO)

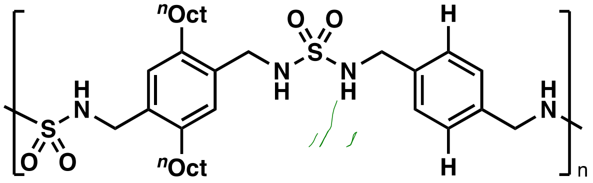

poly-IV-c

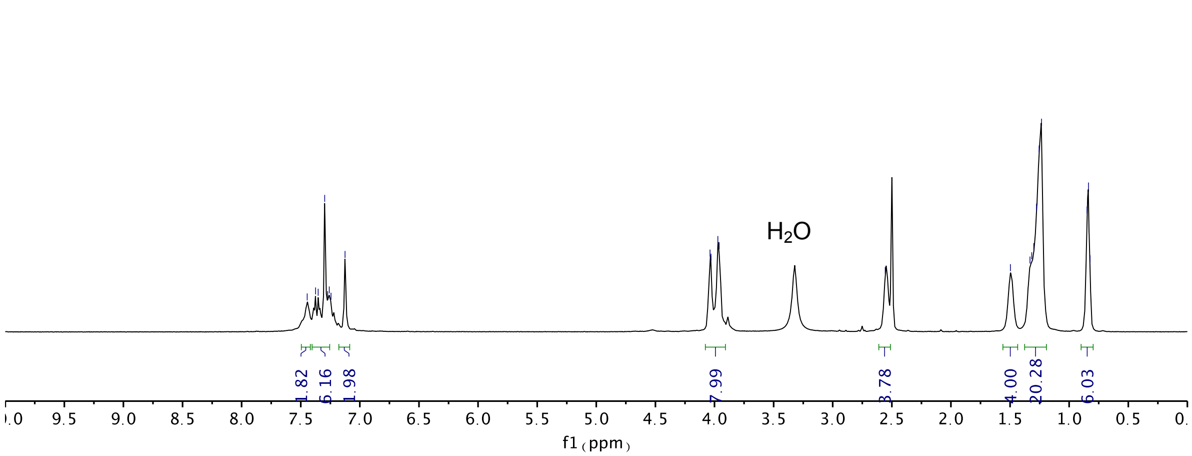

(^{1}H, 500 MHz, d_6-DMSO)

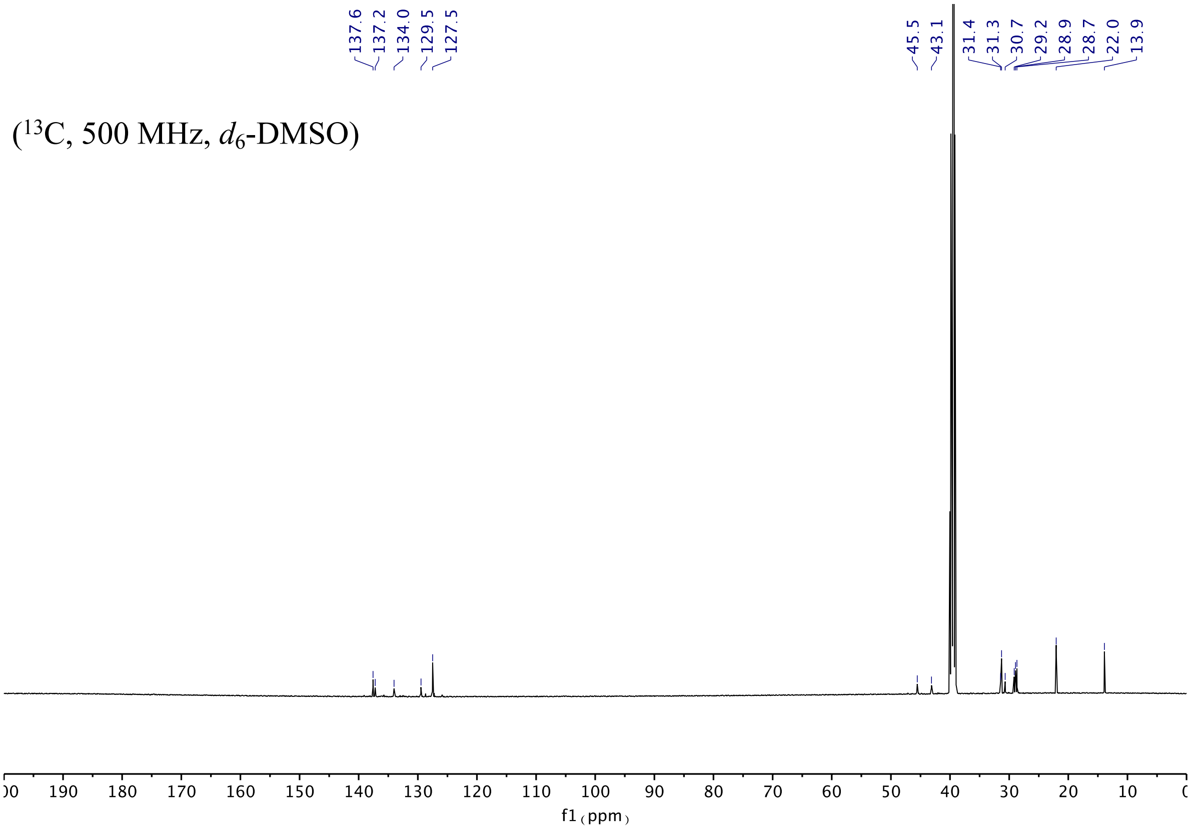

(^{13}C, 500 MHz, d_6-DMSO)

References

1. Kulow, R. W.; Wu, J. W.; Kim, C.; Michaudel, Q., Synthesis of unsymmetrical sulfamides and polysulfamides via SuFEx click chemistry. *Chem. Sci* **2020**, *11*, 7807–7812.
